# Discovery of potent BET bromodomain 1 stereoselective inhibitors using DNA-encoded chemical library selections

**DOI:** 10.1073/pnas.2122506119

**Published:** 2022-05-27

**Authors:** Ram K. Modukuri, Zhifeng Yu, Zhi Tan, Hai Minh Ta, Melek Nihan Ucisik, Zhuang Jin, Justin L. Anglin, Kiran L. Sharma, Pranavanand Nyshadham, Feng Li, Kevin Riehle, John C. Faver, Kevin Duong, Sureshbabu Nagarajan, Nicholas Simmons, Stephen S. Palmer, Mingxing Teng, Damian W. Young, Joanna S. Yi, Choel Kim, Martin M. Matzuk

**Affiliations:** ^a^Center for Drug Discovery, Department of Pathology & Immunology, Baylor College of Medicine, Houston, TX 77030;; ^b^Department of Pharmacology and Chemical Biology, Baylor College of Medicine, Houston, TX 77030;; ^c^Department of Pediatrics, Texas Children’s Hospital, Baylor College of Medicine, Houston, TX 77030

**Keywords:** BET bromodomains, BRDT, DNA-encoded chemistry technology, small-molecule inhibitors

## Abstract

BRDT, BRD2, BRD3, and BRD4 comprise the bromodomain and extraterminal (BET) subfamily which contain two similar tandem bromodomains (BD1 and BD2). Selective BD1 inhibition phenocopies effects of tandem BET BD inhibition both in cancer models and, as we and others have reported of BRDT, in the testes. To find novel BET BD1 binders, we screened >4.5 billion molecules from our DNA-encoded chemical libraries with BRDT-BD1 or BRDT-BD2 proteins in parallel. A compound series enriched only by BRDT-BD1 was resynthesized off-DNA, uncovering a potent chiral compound, CDD-724, with >2,000-fold selectivity for inhibiting BRDT-BD1 over BRDT-BD2. CDD-724 stereoisomers exhibited remarkable differences in inhibiting BRDT-BD1, with the *R*-enantiomer (CDD-787) being 50-fold more potent than the *S*-enantiomer (CDD-786). From structure–activity relationship studies, we produced CDD-956, which maintained picomolar BET BD1 binding potency and high selectivity over BET BD2 proteins and had improved stability in human liver microsomes over CDD-787. BROMO*scan* profiling confirmed the excellent pan-BET BD1 affinity and selectivity of CDD-787 and CDD-956 on BD1 versus BD2 and all other BD-containing proteins. A cocrystal structure of BRDT-BD1 bound with CDD-956 was determined at 1.82 Å and revealed BRDT-BD1–specific contacts with the αZ and αC helices that explain the high affinity and selectivity for BET BD1 versus BD2. CDD-787 and CDD-956 maintain cellular BD1-selectivity in NanoBRET assays and show potent antileukemic activity in acute myeloid leukemia cell lines. These BET BD1-specific and highly potent compounds are structurally unique and provide insight into the importance of chirality to achieve BET specificity.

## Significance

BET bromodomain inhibition is therapeutic in multiple diseases; however, pan-BET inhibitors have induced significant myelosuppression and gastrointestinal toxicity, perhaps due to inhibition of both tandem bromodomains (BD) of all BET family members. However, selective inhibition of just the first BD (BD1) phenocopies pan-BET inhibitor activity in preclinical models of cancer, other diseases, and, for BRDT, in the testes for a contraceptive effect. Here, we leveraged our multibillion-molecule collection of DNA-encoded chemical libraries (DECLs) to identify BET BD1-selective inhibitors of specific chirality with high potency, stability, and good cellular activity. Our findings highlight the robustness and efficiency of the DECL platform to identify specific, potent protein binders that have promise as potential anticancer and anti-inflammatory agents and as male contraceptives.

BRDT, a testis-specific bromodomain-containing protein, along with BRD2, BRD3, and BRD4, are members of the bromodomain and extraterminal (BET) family, and each protein contains two tandem bromodomains (1). Bromodomain-containing proteins are implicated in cancer (2), inflammation (3), infectious diseases (4, 5), and metabolic disorders (6). We and others have also shown that BRDT is a validated germ-cell target for nonhormonal contraceptive development (7). First-generation pan-BET inhibitors such as **JQ1** (8) have proven useful as tool compounds in these studies, but the short half-life and rapid metabolism of **JQ1** (9) have motivated researchers to develop other pan-BET inhibitors. Some of these inhibitors have progressed or are currently progressing through phase I or II clinical trials for oncology indications (10). However, some of the more promising initial agents (including OTX-015 and IBET-151) demonstrated limited single-agent efficacy, halting further clinical development (11). Furthermore, dose-limiting thrombocytopenia appears to be a common side effect among all pan-BET inhibitors, which may be due to poor selectivity among BET family members and between BD1 and BD2 (10). Thus, the challenge of overcoming off-target tissue toxicity while maintaining antitumor efficacy remains for all pan-BET small-molecule inhibitors. Recent evidence indicates small-molecule inhibition of the bromodomain 1 (BD1) of BRD4 has similar effects to inhibition of both bromodomains (12–15), while inhibition of BD2 may have more selective effects (12, 16). In analogous fashion, it is the first bromodomain of BRDT (BRDT-BD1) that has been shown to be specifically essential for fertility in mice (17–19). Therefore, BD1-specific BET inhibitors have been sought as both disease-fighting therapeutics and also for eventual translation as nonhormonal contraceptives.

BD1 and BD2 share a high degree of sequence homology between BET family members. However, more significant structural differences exist between BD1 and BD2 themselves that can be exploited for selective ligand development to either domain (20). The compound RVX-208 was an early BD2-specific compound; however, it exhibited only moderate BD2 potency (>100 nM, IC_50_ [the concentration that inhibits response by 50%] = 2 µM in our BRDT-BD2 AlphaScreen assay) and selectivity over BD1 domains (∼10-fold) (21). More recent efforts from other laboratories and ours have reported the synthesis of pan-BD2 inhibitors with significantly enhanced potency and selectivity [e.g., GSK046 (12), ABBV-744 (22), and our **CDD-1302** compound (23)].

Comparably, extensive efforts have been directed toward the discovery of BET BD1-specific inhibitors, starting from the early identification of less-potent pan-BD1-biased small molecules (13, 14, 24–30). Recently, LT052 was reported to be the first potent inhibitor with 138-fold selectivity for BRD4-BD1 over BRD4-BD2 as confirmed in BROMO*scan* assays. In addition, LT052 shows 88 nM (IC_50_) in a BRD4-BD1 AlphaScreen assay but weak binding to BRD2-BD1 (IC_50_ = 9 µM) (31). In a gout arthritis rat model, LT052 possesses potent anti-inflammatory activity by mediating the BRD4/NF-κB/NLRP3 signaling pathway. Shortly thereafter, two GSK compounds (GSK778 and GSK789) were reported as more potent or more selective pan-BD1 inhibitors, both developed using a structure-based medicinal chemistry design from pan-BET bromodomain inhibitors. GSK778, an analog of I-BET151, has increased potency on BRD4-BD1 and similar selectivity over BRD4-BD2 compared to LT052 [IC_50_ = 41 nM, 143-fold selectivity by time-resolved fluorescence energy transfer, and *K*_d_ (dissociation constant) = 19 nM, 131-fold selectivity by SPR (surface plasmon resonance) (12)]. BROMO*scan* analysis of GSK778 indicated the binding of this compound only to BET bromodomains with strong binding to BET BD1 domains while weakly binding to BET BD2 domains. GSK789 was derived from a series of naphthyridone ATAD2 inhibitors. While GSK789 was less selective (TAF1-BD2 *K*_d_ = 50 nM and TAF1L-BD2 *K*_d_ = 398 nM), it exhibits improved BET BD1 selectivity over GSK778 (*K*_d_ values being 16 to 20 nM on BET BD1s, selectivity being 1,000- to 6,000-fold over BET BD2s by BROMO*scan* profile) (20).

To identify novel BRDT-BD1 inhibitors, we embarked on a screening campaign using DNA-encoded chemical libraries (DECLs), which are becoming an established lead-generation platform for various therapeutic targets. By employing DNA-encoded chemistry technology (DEC-Tec), we previously reported BRDT-BD2–specific binders (23) which have both strong potency and improved selectivity compared to **JQ1** (22, 23). Given that BRDT-BD1 is indispensable for fertility in mice and is a testis-specific contraceptive target (17–19), we performed a parallel screen of BRDT-BD1 and BRDT-BD2 on our DECLs. Here, we demonstrate the discovery of a DEC-Tec inhibitor series that is both potent and highly selective for BET BD1 subfamily members versus BD2. Confirmed by BROMO*scan*, subnanomolar *K*_d_ values were determined for all BET BD1 domains, and >5,000-fold selectivity was identified over BET BD2 domains by *bromoKd*ELECT for BRD3, BRD4, and BRDT, while BRD2 shows 42-fold BD1 selectivity versus BD2. BRDT-BD1 small-molecule cocrystal studies confirmed that the stereochemistry of the compounds drives the selectivity of BD1 versus BD2. BD1-specificity was maintained in cell lines, and marked antileukemic activity was observed. In summary, our work reinforces the importance of chirality to achieve BD1 selectivity, and these highly potent BD1 inhibitors will be useful tools both in the study of spermatogenesis and also for translational studies in other disease models.

## Results

### DEC-Tec Affinity Selection with Bromodomain Proteins.

**Fig. 1.**
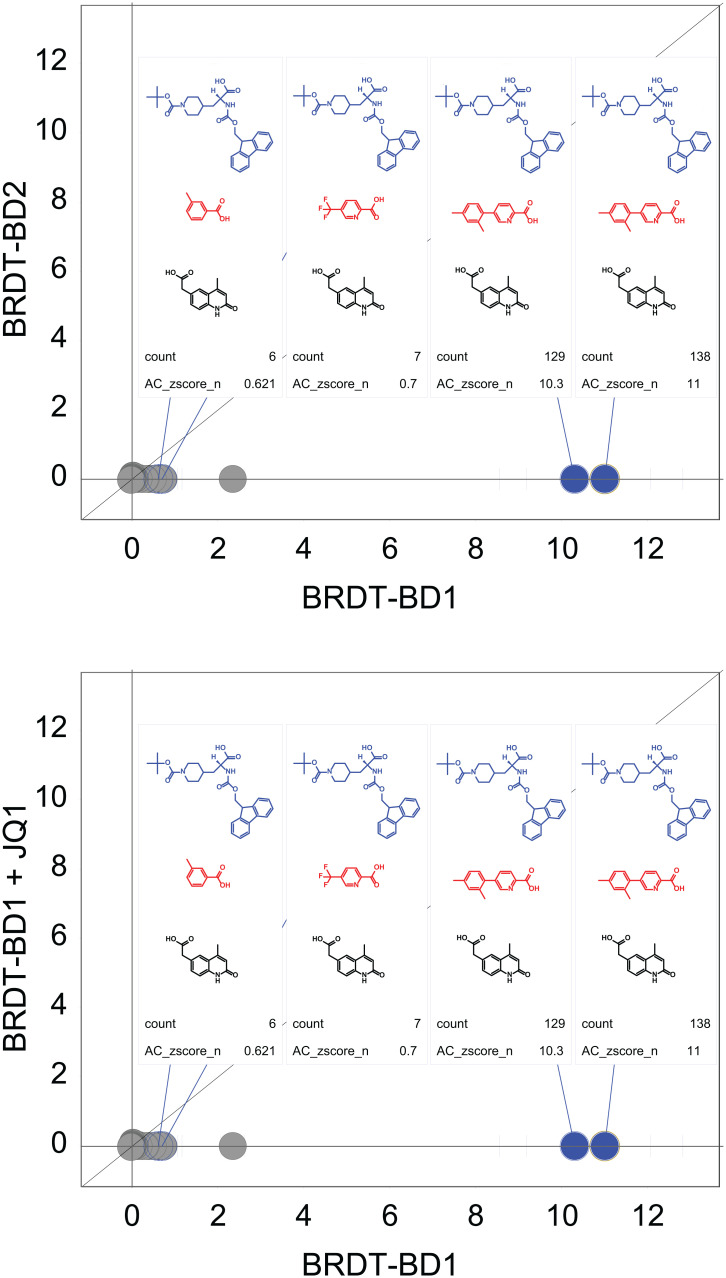
DEC-Tec normalized enrichment results for BRDT-BD1 versus BRDT-BD2 (*Top*) or BRDT-BD1 + JQ1 (*Bottom*). Trisynthons with the best AC_zscore_n and counts are highlighted. These hits show strong enrichment for BRDT-BD1, high selectivity versus BRDT-BD2 (*Top*), and competition with **JQ1** (*Bottom*). For each highlighted trisynthon, the building blocks for cycles 1, 2, and 3 are presented in blue, red, and black, respectively.

To identify small molecules that bind specifically to BRDT-BD1, 50 unique DECLs cumulatively containing >4.5 billion compounds were pooled together for parallel screening of both BRDT-BD1 versus BRDT-BD2. Each library was first quantified by qPCR, and library pooling was then conducted to have ∼1 million copies of each compound present in the pool. BRDT-BD1 and BRDT-BD2 proteins were individually subjected to three rounds of affinity selection at a protein concentration of 0.3 μM. Two control affinity selections were performed in parallel: 1) without protein as a no-target control to identify any nonspecific bead binders and 2) with the addition of 100 μM of the pan-bromodomain inhibitor **JQ1** to identify competitive BRDT bromodomain binders. Illumina next-generation sequencing of the amplified, eluted binders of our targets identified a chemical series consistently enriched with excellent structure–enrichment relationships (SER) from one of our DEC-Tec libraries (Fig. 1). As shown in Fig. 1, strong multiple enrichments were identified with different DNA linkers; these hits had an excellent, normalized Z-score (AC_zscore_n) metric for BRDT-BD1 but not BRDT-BD2 (31). These hits also demonstrated competition with our reference compound **JQ1**. The observed chemical series contained propanoic acids in cycle 1, substituted pyridines or phenyls as cycle 2, and 2-(2-oxo-1,2-dihydroquinolin-6-yl) acetic acid in cycle 3. From SER studies, different substitutions on pyridine cycle 2 resulted in different Z-scores. Altogether, the strong enrichment and reasonable SER suggested a promising three-cycle chemical series and inspired us to further investigate these compounds by synthesizing molecules off-DNA.

### Validation and Synthesis of BD1 DEC-Tec Selection Hits.

**Fig. 2.**
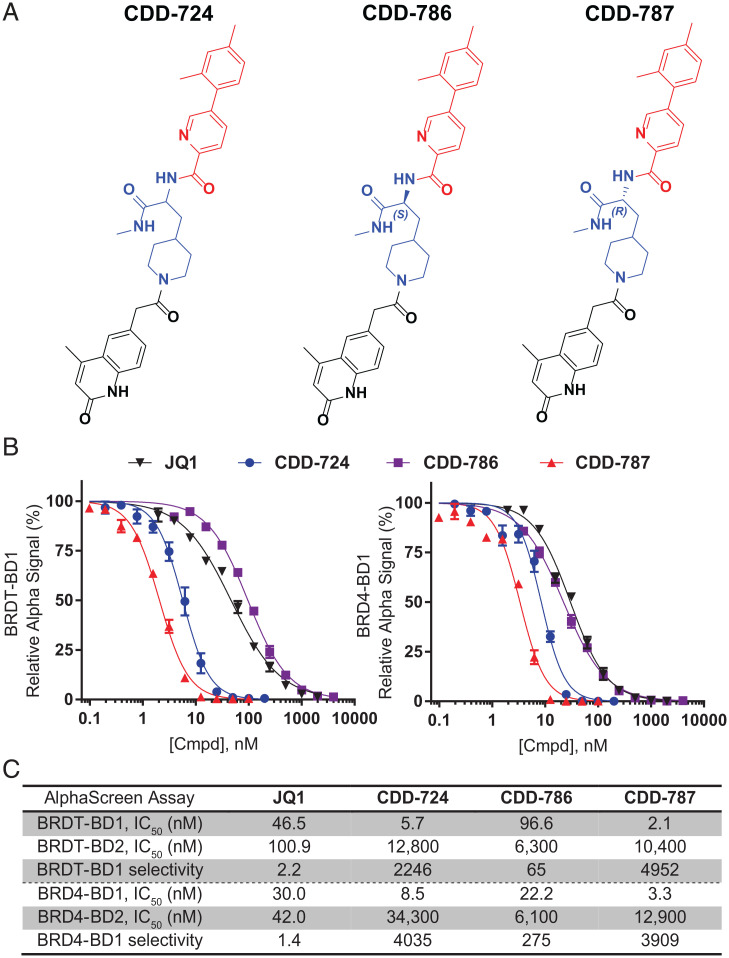
BD1-specific compounds from DECLs. (*A*) Structures of compounds **CDD-724** (racemic compound **6a**), **CDD-786** (compound **6b**), and **CDD-787** (compound **6c**). (*B*) BRDT-BD1 and BRD4-BD1 inhibition of lead compounds compared to **JQ1** (black) as measured by AlphaScreen. (*C*) Comparative potencies of each compound against the first and second bromodomains of BRDT and BRD4, with BD1, fold selectivity immediately below. GraphPad Prism software was used to generate inhibition fitting curves and to determine IC_50_ values.

**Scheme 1.**
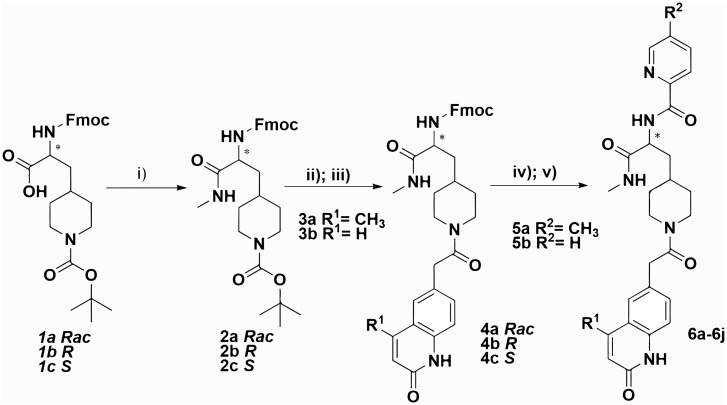
Syntheses of DEC-Tec selection hits and tier 1 SAR **6a**-**6j**. Reagents and conditions: i) Methylamine HCl, *O*-(7-azabenzotriazol-1-yl)-*N*,*N*,*N′*,*N′*-tetramethyluronium hexafluorophosphate (HATU), *N*,*N*-diisopropylethylamine (DIEA), *N*,*N*-dimethylformamide (DMF), room temperature, 1 h; ii) 4 M HCl/dioxane, 1 h; iii) Substituted 2-(2-oxo-1,2-dihydroquinolin-6-yl) acetic acid, HATU, DIEA, DMF, 12 h; iv) 10% Piperidine, DMF, 1 h; v) Different substituted picolinic acids, HATU, DIEA, DMF, 12 h.

Candidate hit molecules were synthesized by truncating the DNA barcode linkage down to a methyl amide (Table 1). The prioritization of compounds to synthesize off-DNA for hit confirmation was based on sequence counts and structural features that were common among the enriched sequences. Accordingly, we selected the three-cycle library hit **CDD-787** (*R*) (138 counts), its antipode **CDD-786** (*S*), and the racemic version **CDD-724** (267 counts, Fig. 2*A*) for initial synthesis. The synthetic route of the DEC-Tec hits is illustrated in Scheme 1 and *SI Appendix*, Scheme S1. Substituted piperidine propanoic acids (**1a–c**) underwent sequential *O*-(7-aza benzotriazole-1-yl)-*N*,*N*,*N*,*N′*-tetramethyluronium hexafluorophosphate (HATU)-mediated amide coupling to produce **2a–c**. Deprotection of the tert-butoxycarbonyl group followed by another HATU-mediated amide coupling using different substituted 2-(2-oxo-1,2-dihydroquinolin-6-yl)acetic acids provided intermediates **4a–c**. Subsequent deprotection of fluorenylmethyloxycarbonyl group followed by a final amide coupling reaction furnished the desired products (**6a**–**6j**).

**Table 1.**
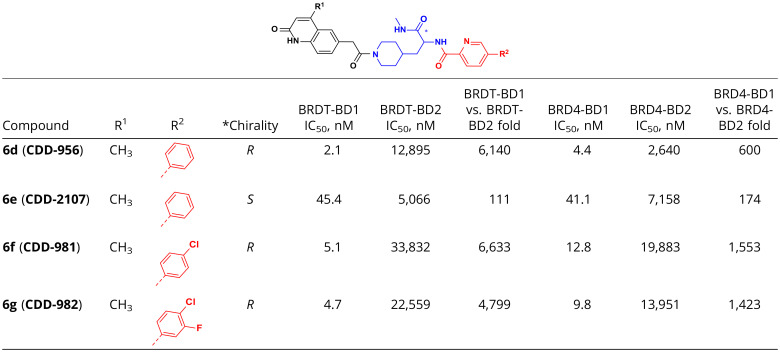
Structures and activities of analogs 6d to 6g

**CDD-724** and its two enantiomers **CDD-786** and **CDD-787** were functionally screened for BRDT-BD1 activity using an AlphaScreen competition assay with biotinylated **JQ1** as the ligand. All three compounds demonstrated potent BRDT-BD1 inhibitory activities with nanomolar IC_50_ values (Fig. 2*B*, *Left* and Fig. 2*C*). These compounds were improved in their selectivity of BRDT-BD1 over BRDT-BD2 (∼65- to 4,952-fold) compared to the twofold BD1 selectivity of the pan-BET inhibitor (+)-**JQ1** (Fig. 2*C*; Table 1). Using a similar AlphaScreen assay, we also tested the compounds for their BRD4 activity and found similar BD1 preference (Fig. 2*B*, *Right*). We noted that **CDD-786** has a fourfold greater affinity to BRD4-BD1 over BRDT-BD1. From the initial AlphaScreen assay, **CDD-787**, with the *R* stereochemical configuration, emerged as the most selective BRDT-BD1 inhibitor with single-digit nanomolar activity (IC_50_ 2.1 nM) and ∼5,000-fold selectivity over BRDT-BD2. Thus, **CDD-787** is 22-fold more potent and ∼2,200 fold more selective than (+)-**JQ1** (Fig. 2) for BRDT-BD1. The *S* enantiomer (**CDD-786**) and the racemate (**CDD-724**) gave IC_50_ values of 96.6 nM and 5.7 nM, respectively, to BRDT-BD1 and displayed 65-fold and 2,246-fold selectivity, respectively, over BRDT-BD2.

### Exploration of Structure–Activity Relationships (SAR).

Based on the promising inhibition of the compounds that were most enriched by the selection, we pursued SAR around the pyridine and 1,2-dihydroquinolinone rings (Table 1). Our goal was to identify compounds that struck an optimal balance between potency and selectivity for BET BD1 domains versus BET BD2 domains. We first explored whether the 3,5-dimethyl phenyl substitution on the pyridine ring could be replaced with a simple phenyl ring. Removal of the methyl groups on the phenyl ring of the *R* isomer provided **CDD-956**, which exhibited superior potency (IC_50_ 2.1 nM and 4.4 nM to BRDT-BD1 and BRD4-BD1, respectively). However, the similar deletion of the methyl groups on the *S* isomer (**CDD-2107**) and the racemate (**CDD-1146**) resulted in a 3.6-fold and 21-fold drop in potency to BRDT-BD1 compared to the parent **CDD-787**. For the *R* isomeric series, monohalogenation (**CDD-981**) and dihalogenation (**CDD-982**) on the phenyl ring maintained excellent potency to BRDT-BD1 (IC_50_ 5.1 nM and 4.7 nM, respectively), but their BRDT-BD1:BRDT-BD2 selectivity was decreased compared to **CDD-787**. Compound **CDD-784**, which contained no phenyl substitution on the pyridine ring, sustained BD1 potency but underwent a fivefold loss of selectivity to BRDT-BD2 compared to the **CDD-787**. We next focused on the 1,2-dihydroquinoline moiety (*SI Appendix*, Table S1). Removal of the methyl group on 1,2-dihydroquinolinone **CDD-986** (*R*) provided a single-digit nanomolar potency for both BRDT-BD1 and BRD4-BD1 but gave a 1.6-fold loss of selectivity over BRD4-BD2 (*SI Appendix*, Table S1).

We conducted a second round of SAR focused on racemates and truncation of the amide on the propenamide linker and replacement of 5-substituted pyridines with diverse quinolines (*SI Appendix*, Table S2). To achieve truncated amides, we followed Route A and Route B (*SI Appendix*, Scheme S1). In Route A, the piperidine end was protected, whereas in Route B the propenamide end was protected with a tert-butyloxycarbonyl group. In the case of compound **CDD-906**, replacing methyl instead of amide linker exhibited reduced potency (IC_50_ 31 nM) and 6.5-fold loss of selectivity on BRDT-BD1. Further modifications on phenyl pyridines, such as in **CDD-813**, **CDD-767,** and **CDD-779** containing truncated amides, demonstrated dramatic drop of activities. Introduction of quinoline (**CDD-855**), iso-quinolines (**CDD-854** and **CDD-853**), and piperazine (**CDD-778**) did not improve either selectivity or potency. These results demonstrated that both the amide and the phenyl pyridine functionalities are crucial for maintaining activity. Overall, it was concluded from these SAR studies that compounds in the (*R*) isomeric series, such as **CDD-956**, **CDD-981**, **CDD-982**, and **CDD-986**, gave excellent BRDT-BD1 potency with superior selectivity over BRDT-BD2.

### Mosher Method to Determine Stereochemistry.

Because our AlphaScreen findings revealed the importance of chirality for BD1 selectivity, we next sought to determine the absolute stereochemistry of our lead compounds. For this purpose, we utilized the Mosher method, which is a reliable method for assigning the absolute stereochemistry of a stereogenic center by NMR (32). This method usually employs a secondary stereogenic carbon-bearing alcohol or amine that is converted to an ester or amide, respectively, by coupling α-methoxy-α-trifluoromethylphenyl acetic acid (MTPA) (33). With liquid chromatography–mass spectrometry chiral analysis and purity presented in the supporting information (*SI Appendix*, Scheme S2 and Table S3), our most potent compounds, **CDD-787** and **CDD-956**, were confirmed to be *R* isomers.

### CDD-956 Is More Metabolically Stable.

Given these encouraging findings, we followed up these results by determining metabolic stabilities of **CDD-787** and **CDD-956** in mouse and human liver microsomes. In mouse liver microsomes, **CDD-787** is labile, like **JQ1**, while the stability of **CDD-956** is improved, as evidenced by their half-life data. In human liver microsomes, the half-life of **CDD-956** is 139 min, while **CDD-787** is 33 min, and **JQ1** remains poor (Table 2).

**Table 2.** Metabolic stability of CDD-956 and CDD-787 in MLM and HLM

	Assay (half-life), min*	CDD-787	CDD-956	JQ1	Alprazolam
1	MLM t_1/2_	13	35	13	302
2	HLM t_1/2_	33	139	10.3	864

Final concentrations: liver microsomes: 0.5 mg protein/mL, compound concentration: 2.0 μM, NADPH concentration: 1.0 mM; **JQ1**: short half-life control, Alprazolam: long half-life control. In duplicate at 0, 5, 10, 20, 40, and 60 min. HLM/MLM, human and mouse liver microsomes.

*The half-life less than 30 min in MLM and HLM was generally considered as unstable.

### CDD-787 and CDD-956 are Picomolar BET BD1 Inhibitors.

**Fig. 3.**
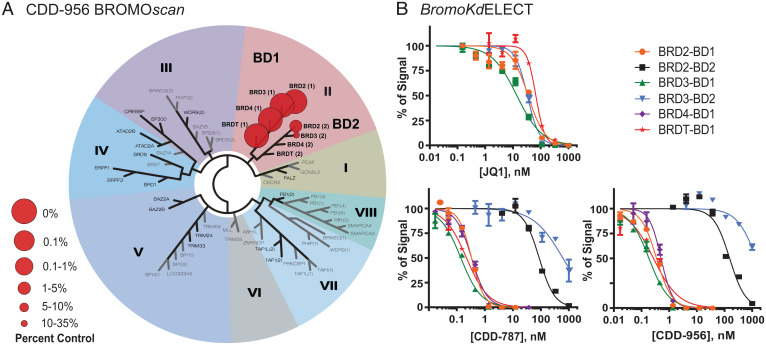
Selectivity of CDD compounds for BD1 of the BET family. (*A*) BROMO*scan* profiling of all available human bromodomains with **CDD-956** was performed at 1,000 nM. Phylogenetic tree of bromodomains demonstrating preferential binding of **CDD-956** for the BET subfamily BD1 domains using the BROMO*scan* bromodomain competition binding assay. Binding to all bromodomains is presented in *SI Appendix*, Table S5. Percent Control: **CDD-956** was screened at 1,000 nM, and results for binding interactions are reported as percent control, where lower percentages indicate stronger hits in the matrix. Detailed percent control calculation and protocol description in *SI Appendix*, section 9. (*B*) *BromoKd*ELECT dose–response curves confirmed that **CDD-787** and **CDD-956** are strong binders to the BET subfamily BD1 domains, with the highest affinity for BRDT-BD1 (see also Table 3).

To assess the selectivity of **CDD-956**, we performed BROMO*scan* profiling and identified high selectivity for the BET family, and primarily BD1 of all BET family members (Fig. 3*A*). **CDD-956** also had some binding to BD2 of BRD2 and BRD3, while exhibiting no binding in the BROMO*scan* profiling to BRD4-BD2 nor BRDT-BD2. We followed this up by comparing the binding affinity in the *bromoKd*ELECT assay (Fig. 3*B* and Table 3). Here, we observed both **CDD-787** and **CDD-956** exhibited picomolar inhibition against BRD2-BDT1 (IC_50_ 260 pM and 310 pM), BRD3-BD1 (IC_50_ 130 pM and 180 pM), BRD4-BD1 (IC_50_ 290 pM and 440 pM), and BRDT-BD1 (IC_50_ 220 pM and 330 pM), respectively; this is outstanding compared with our reference **JQ1**, whose IC_50_ ranged from 39 nM to 65 nM. In the case of the BRD2-BD1 selectivity, both CDD inhibitors showed superior selectivity (420-fold for **CDD-787** and 480-fold **CDD-956**) over BRD2-BD2, whereas in the case of BRD3-BD1 selectivity **CDD-787** exhibited more selectivity than **CDD-956** and showed 5,920-fold selectivity over BRD3-BD2.

**Table 3.** Compound binding constants (*K*_d_) determined by *BromoKd*ELECT assay

*K*_d_, nM	JQ1	CDD-787	CDD-956
BRD2-BD1	39	0.26	0.31
BRD2-BD2		110	150
BRD2-BD1 selectivity		420	480
BRD3-BD1	16	0.13	0.18
BRD3-BD2		770	1,000
BRD3-BD1 selectivity		5,920	5,550
BRD4-BD1	30	0.29	0.44
BRDT-BD1	65	0.22	0.33

A detailed protocol description is given in *SI Appendix*, section 9.

### A Cocrystal Structure Validates the Importance of *R* Stereochemistry in BD1 Selectivity.

**Fig. 4.**
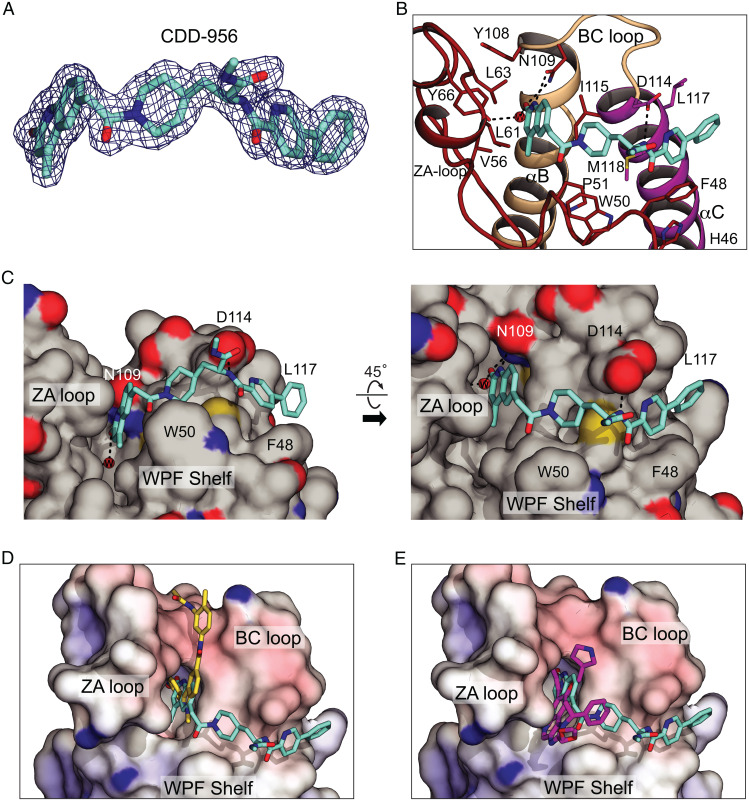
Detailed interactions between BRDT-BD1 and **CDD-956**. (*A*) *2Fo-Fc* map of **CDD-956** in complex with BRDT-BD1 contoured at 1*σ*. (*B*) Detailed interaction between BRDT-BD1 and **CDD-956**. Carbon atoms of **CDD-956** is colored in cyan and an ordered water molecule that mediates the Y66:methylquinoline interaction in a red sphere. The ZA loop is colored in red, the αC helix in magenta, and the rest in tan. Key interacting residues shown in sticks. (*C*) Views of the **CDD-956** binding surface. The surface is colored in gray except for side-chain noncarbon atoms; oxygen atoms are colored in red, nitrogens in blue, and sulfurs in yellow. (*Right*) The view is the same as in *B* and is retained in *D* and *E*. (*D*) The BRDT-BD2/**CDD-1302** complex (PDB ID: 7L99) is superimposed with the BRDT-BD1/**CDD-956** complex; the electrostatic potential surface belonging to the BRDT-BD1/**CDD-956** complex is shown. **CDD-1302** yellow is colored yellow, **CDD-956** is colored cyan. They align with an RMSD value of 0.56 Å between shared 100 CA atoms. Both inhibitors occupy the KAc pocket but they access different adjacent grooves. (*E*) The BRD4-BD1/iBET-BD1 complex (PDB ID: 6SWN) is superimposed with the BRDT-BD1/**CDD-956** complex. iBET-BD1 is colored magenta; **CDD-956** is colored cyan. They align with an RMSD value of 0.95 Å between shared 95 CA atoms. Both inhibitors occupy the KAc pocket, but only **CDD-956** extends significantly from this pocket.

To understand the structural basis of the high affinity and selectivity, we determined the crystal structure of BRDT-BD1 bound with **CDD-956** at 1.82-Å resolution (Fig. 4*A* and *SI Appendix*, Table S4) (Protein Data Bank [PDB] ID: 7UBO). The BRDT-BD1/**CDD-956** crystal contained four molecules per asymmetric unit (*SI Appendix*, Fig. S1*A*) that are very similar, showing root-mean-square deviation (RMSD) values of >0.5 Å between shared ∼100 CA atoms (*SI Appendix*, Fig. S1*B*). These four domains all show very similar poses of the **CDD-956** ligand contacting the KAc (acetylated lysine) binding pocket and a neighboring hydrophobic groove. Although all four **CDD-956** molecules participate in crystal-packing interactions, the binding poses of **CDD-956** are nearly identical (*SI Appendix*, Fig. S1*B*), suggesting that these contacts minimally influence the binding mode.

**CDD-956** binds to the KAc binding pocket, the WPF shelf, and a shallow hydrophobic groove formed between αZ and αC helices (Fig. 4 *B* and *C* and *SI Appendix*, Fig. S2). The methyl-quinoline binds to the KAc pocket via hydrogen bonds directly with the conserved asparagine (N109) at the BC loop and indirectly with Y66 at the ZA loop through ordered water. The methyl-quinoline interacts with hydrophobic residues F52, V56, L61, and L63 that line the outer rim of the KAc pocket. Several ordered waters are present in the pocket, including one that mediates the Y66:methyl-quinoline interaction. The piperidine-amide linker interacts with the WPF shelf and the αC helix, and the amide forms a hydrogen bond with D114 at the beginning of the αC helix (Fig. 4*B*). The phenyl-pyridine docks to a shallow hydrophobic groove between the αZ and αC helices. Side chains of F48 (αZ), D114 (αC), L117(αC), and M118 (αC) form the hydrophobic groove (Fig. 4*C*). The pyridine orients perpendicular to the side chains of F48 and D114 on either side and interacts with these residues and the side chain of M118 through van der Waals (vdW) contacts. The plane of the phenyl ring is twisted 38° relative to the pyridine ring and packs against the side chain of L117(αC) (Fig. 4*C*). Comparison of the binding modes of **CDD-956** with BD1, **CDD-1302** with BRDT-BD2 (23), and iBET-BD1 (GSK778) with BRD4-BD1 (12) (Fig. 4 *D* and *E*) shows that our BD1-selective and BD2-selective DECL-derived inhibitors each occupy the same KAc pocket as GSK778 but also access adjacent grooves that differ between the two domain types.

Structural comparisons between this structure and previous BD domains explain the high BD1:BD2 selectivity seen for **CDD-956** based on three key residues that differ between the domains: F48, L117, and D114. Phenylalanine 48 (αZ) is replaced with tyrosine in all BD2s (Y291 in BRDT). The hydrophobic groove to which **CDD-956** binds is missing in BRDT-BD2 because the side chain of Y291 points away from the BC loop and hydrogen bonds with H287 located one helix turn below. L117(αC) is replaced in BD2s with threonine (BRDT) or alanine (BRD2, BRD3, and BRD4), reducing the vdW contact surface with the phenyl group of **CDD-956**. Aspartate 114, which packs with and forms hydrogen bonds to the amide linker, is replaced with glutamate in all BD2s except BRD2. While other BD2s cannot form the critical hydrogen bond with the amide linker seen in our structure, and may clash with the phenyl-pyridine, BRD2-BD2 can presumably make interactions similar to those seen in our complex. The presence of this aspartate explains why BRD2-BD2 binds **CDD-956** tighter than BRD3-BD2 (Fig. 4*C*).

Our structure explains the potency of the DECL library hit and the relative affinities of the *R* and *S* enantiomers of the initial hit and of subsequent analogous. The methyl groups of **CDD-787**, the *R* version of the original hit, can be accommodated in the structure without steric clash. Interestingly, intraligand forces on the ortho-methyl of **CDD-787** would twist the phenyl ring out of the plane with respect to the pyridine by default, as occurs in the BRDT-BD1/**CDD-956** complex even in the absence of such a methyl-pyridine steric repulsion. The *R* enantiomer poses the methyl-amide DNA linker site so that it points away from the protein surface in our structure (Fig. 4*C*) which would facilitate robust interaction in the DECL selection process. The midnanomolar affinities of **CDD-2107** or **CDD-786** (Table 1 and Fig. 2) suggest that the methyl-quinoline and phenyl-pyridine moieties of the *S* species likely interact with BRDT-BD1 like the *R* ligand **CDD-956** in our structure; this would point the methyl-amide of the *S* species toward W50 rather than toward solvent, incurring steric clashes for many of the possible rotamers of this substituent. Like the methyl of **CDD-787**, the halides of **CDD-981** and **CDD-982** (Table 1) can be accommodated without clashes.

### CDD *R* Enantiomers Maintain Their BD1 Selectivity in Cells.

**Fig. 5.**
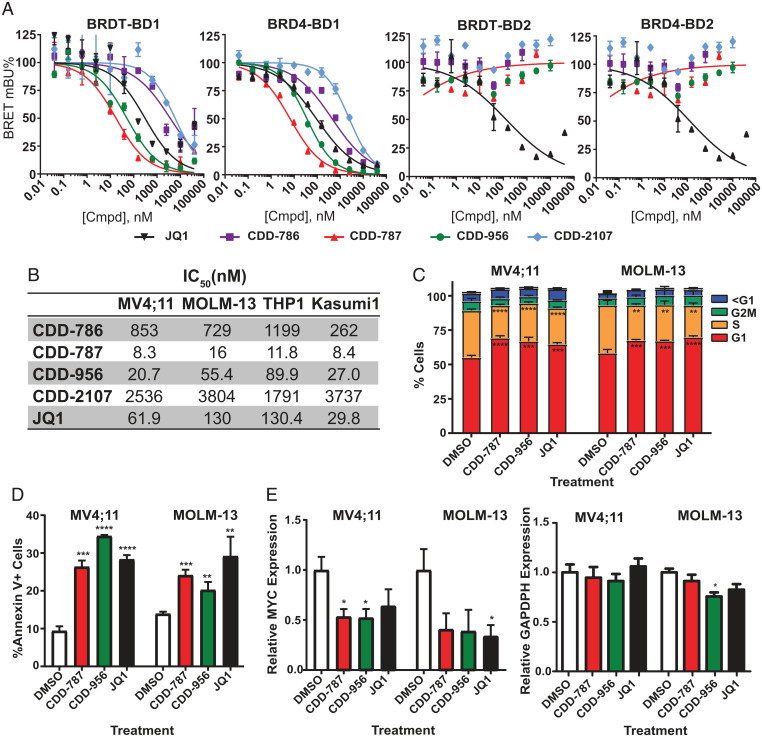
CDD-787 and **CDD-956** maintain BD1 selectivity, potency, and activity in cellular models. (*A*) NanoBRET assays of compounds **CDD-786**, **CDD-787**, **CDD-956**, and **CDD-2107**. (*B*) Viability IC_50_ of CDD compounds on 4 AML cell lines after 72 h of treatment by CellTiterGlo detection. Results were normalized to dimethyl sulfoxide (DMSO)-treated cells for the same length of time. (*C*) Effect of **CDD-787** and **CDD-956** on the cell cycle of MV4;11 and MOLM-13 cells after 24 h of treatment at the IC_50_. (*D*) Detection of Annexin V+ cells after 72 h of treatment with **CDD-787** and **CDD-956**. (*E*) Expression of *MYC* and *GAPDH* by RT-qPCR after 8 h of **CDD-787** and **CDD-956**. For all experiments, **JQ1** is used as a control. *P < 0.05, **0.001 < *P* < 0.01, ***0.0001 < *P* < 0.001, and ****P < 0.0001.

To confirm our compounds were cell-penetrant and active, the cellular binding of **CDD-956**, **CDD-2107**, **CDD-786**, and **CDD-787** to BET bromodomains was correspondingly studied using a NanoBRET target engagement assay that measures compound binding at select target proteins within intact cells. The inhibition of **CDD-956** and **CDD-787** on tracer binding was found to be more potent and selective for NanoLuc-BET BD1 in transiently transfected HEK293 cells (Fig. 5*A*). There was no effect of our compounds against BRDT-BD2 or BRD4-BD2, compared to **JQ1,** which binds both BD1 and BD2 equally.

### CDD-787 and CDD-956 Exhibit Potency in Acute Myeloid Leukemia (AML) Cell Lines.

High levels of BRD4 are observed in multiple cancers including leukemias [DepMap (34)]. To determine the anticancer effects of our BD1-selective compounds, we treated BRD4-dependent AML cell lines with a range of doses to determine the IC_50_s of each agent. Our compounds had cellular potency similar to that we detected in our NanoBRET assays, with **CDD-786** having minimal effect and **CDD-787** and **CDD-956** having similar or slightly improved cellular potency compared with **JQ1** (Fig. 5*B*). **CDD-2107** (the *S*-enantiomer of **CDD-956**) had minimal activity in the AML cell lines in analogous fashion to **CDD-786**. We confirmed that both **CDD-787** and **CDD-956** induced G1 arrest and apoptosis in both MV4;11 and MOLM-13 cells (Fig. 5 *C* and *D*). *MYC* expression was also suppressed after 8 h of treatment with both agents in both cell lines, with no/minimal effects on *GAPDH* (Fig. 5*E*). We compared these findings to those of some of our other SAR compounds from Table 1 and found corresponding cellular potency to their biochemical binding efficacy (*SI Appendix*, Table S6).

## Discussion

Since the report of **JQ1** in 2010 (8), BET bromodomains have emerged as intriguing targets in a variety of diseases, with both tool and clinical compounds reported in the intervening decade. Given the class-wide thrombocytopenia seen with clinical pan-BET inhibitors, recent efforts have focused on BD-specific inhibitors (12–14, 20, 22–30, 35). Our findings of BET BD1-specific inhibitors allow us to dissect the role of this bromodomain specifically in spermatogenesis and in cancer (21). This series of selective and highly potent BET inhibitors were identified in our DEC-Tec screening platform without the need for significant chemical optimization, highlighting the strength of DECL screening to rapidly find potent and specific inhibitors of even structurally different targets. This stands in contrast to conventional high-throughput screening methods that screen <1 million compounds and often only identify modestly potent compounds with no selectivity guaranteed. Our picomolar BET BD1 inhibitors exhibit three orders of magnitude greater selectivity over BET BD2 with an attenuated SAR.

Our discovery of the importance of stereochemistry in achieving BET BD1 selectivity by our SAR approach (and confirmed in the crystal structure) highlights the strength of our DEC-Tec platform. Starting with racemic **CDD-724**, we were able to quickly deduce the importance of the *R* configuration isomers such as **CDD-787** and **CDD-956** for BD1 selectivity. Further SAR on racemates and truncated analogs led to the conclusion that both amide and phenyl pyridine functionalities are crucial, and that the chirality is important for maintaining BD1 specificity. This enantiomeric selectivity illuminates the powerful influence of chirality on molecular recognition, particularly regarding the generation of selective inhibitors to protein subfamily members. From the BROMO*scan* results, our compounds emerged as picomolar inhibitors with far improved selectivity than **JQ1**, which is the standard tool compound for nonselective BET inhibitors. Additionally, our crystal structure of the BRDT-BD1/**CDD-956** complex reveals that **CDD-956** achieves BD1:BD2 selectivity through specific interactions with several unique residues in BD1 including F48, D114, and L117. Moreover, the detailed structure analysis provides insights into the significance of stereochemistry to the BD1 potency, consistent with previous reports. Notably, **CDD-956** occupies a shallow hydrophobic groove formed between the αZ and αC helices, which is not seen in other BD1 inhibitors. Therefore, the binding mode we describe here represents a new opportunity to explore BD1 selective inhibitors.

The significantly improved potency of our compounds over **JQ1** was maintained in cells and BD1 selectivity was preserved. Furthermore, antileukemic activity against *MYC* and induction of apoptosis was also maintained with our compounds. While AML cell lines do not express BRDT, further studies on the ability of these compounds to target BRDT in the testis are underway. Finally, the stability of **CDD-787** and **CDD-956** suggests that these compounds should be useful tools to probe the activity of BD1 in both in vivo cancer and spermatogenesis models.

## Conclusions

Here, we highlight the utility of our DNA-encoded chemistry technology platform to discover BET BD1-specific probes. Previous studies have identified other BET BD1-specific probes; however, using the DEC-Tec approach, compounds with significant potency and selectivity were immediately identified, with minimal synthetic optimization. The chiral nature of our compounds, in combination with our cocrystal structure, revealed the critical interactions needed for achieving high BD1 selectivity. Our compounds are active in BRD4-dependent cell lines and had good liver microsomal stability, suggesting these molecules are potential chemical probes to investigate the roles of BET BD1 in cancer models and BRDT-BD1 in in vivo spermatogenesis models.

## Supplementary Material

Supplementary File

## Data Availability

All study data are included in the article and/or *SI Appendix*.
