# Supplementary material for: Discovery of potent BET bromodomain 1 stereoselective inhibitors using DNA-encoded chemical library selections
Source: Proc Natl Acad Sci U S A. 2022 May 27;119(22):e2122506119. doi: 10.1073/pnas.2122506119 (PMC9295786; doi:10.1073/pnas.2122506119)
Supplement: Supplementary File [file pnas.2122506119.sapp.pdf]

PNAS
www.pnas.org

Supporting Information for

Discovery of potent BET bromodomain 1 stereoselective inhibitors using DNA-encoded chemical library selections

CONFIDENTIAL

Ram K. Modukuri[1]*, Zhifeng Yu[1]*, Zhi Tan[1]*, Hai Minh Ta[2*], Melek Nihan Ucisik[1], Zhuang Jin[1], Justin L. Anglin[1], Kiran L. Sharma[1], Pranavanand Nyshadham[1], Feng Li[1,2], Kevin Riehle[1], John C. Faver[1], Kevin Duong[1,3], Sureshbabu Nagarajan[1], Nicholas Simmons[1], Stephen S. Palmer[1], Mingxing Teng[1,2], Damian W. Young[1,2], Joanna S. Yi[1,3], Choel Kim[1,2], and Martin M. Matzuk[1,2,#]

[1]Center for Drug Discovery, Department of Pathology & Immunology, Baylor College of Medicine, Houston, Texas 77030, United States of America
[2]Department of Pharmacology and Chemical Biology, Baylor College of Medicine, Houston, Texas 77030, United States of America
[3]Department of Pediatrics, Baylor College of Medicine, and Texas Children's Hospital, Houston, TX 77030, United States of America

*, equal author; [#]communicating author: Martin M. Matzuk, MD, PhD, Neurological Research Institute, 6th floor, 1250 Moursund Street, Houston, TX 77030. mmatzuk@bcm.edu 713-798-6451.

This PDF file includes:

Supporting information

Table of Contents

1. Syntheses of compounds
1a. Materials and instrumentation……………………………………………………………………S3
1b. Typical method for the preparation of compounds 2a ……………………………………………S3
1c.1. Synthesis of intermediate2a-2c and NMR data of (2a–2c) …………………………………S3 & S4
1c.2. Typical method for the preparation of compound 4a and NMR data of (4a–4d) ……………...S4-S6
1c.3. Typical method for the preparation of compounds 6a and NMR data of (6a–6j)…………….....S6-S10
1c.4. Typical method for the preparation of compound 8a and NMR data of 8a……………..……...S10
1c.5. Typical method for the preparation of compound 9a and NMR data of 9a……………………..S10
1c.6. Typical method for the preparation of compound 11a and NMR data of (11a-11b)………...S11-S12
1c.7. Typical method for the preparation of compound 12a and NMR data of (12a-12g)…………S12-S15
2. ^{1}H and ^{13}C NMR spectra of most selective compounds, **6a** (**CDD-724**), **6c** (**CDD-787**), **6d** (**CDD-956**), **6f** (**CDD-981**), **6g** (**CDD-982**), **6h** (**CDD-986**), and **6j** (**CDD-1146**) ……….………………S16-S29
3. Tier 2 SAR analysis, Table S1, and Table S2…….……………………………………….... S30-S32
4. Mosher Method and Table S3 and spectra………………………………………….........S33-S36
5. Chiral analysis of compounds **CDD-724**, **CDD-786**, **CDD-787**, **CDD-1146**, **CDD-2107,** and **CDD-956**…..……………………………………………………………..………………... S37-S39
Figure S1………..…………………………………………………………………………S40
Figure S2……………………………………………………………………………………..S41
6. Data collection and refinement of the crystal structure and Table S4…………………………..S42-S43
7. BROMO*scan* data of **CDD-956** against all BET BDs and Table S5 ……………….…………….....S44
8. Viability EC_{50} of selected compounds against AML cell line MV4;11and Table S6……...……..….S45
9. Material and Methods…..……………………………..……………………………….S45-S49
10. Supporting References……….……….…………………………………………….......S50

1. Syntheses of Compounds

1a. Materials and instrumentation. All reactions involving air-sensitive reagents were carried out in anhydrous solvents under an atmosphere of nitrogen. Reagents and solvents purchased from commercial supplies were used as received. Reactions were monitored by thin-layer chromatography (TLC) on Baker-flex® silica gel plates (IB2-F) using UV-light (254 and 365 nm) detection or high-performance liquid chromatography/mass spectrometry (HPLC-MS). Column chromatography was carried out using Teledyne ISCO CombiFlash system equipped with either a silica or C-18 column. NMR spectra were recorded at room temperature using a Bruker Avance III HD 600 MHz spectrometer (^{1}H NMR at 600 MHz and ^{13}C NMR at 150 MHz) or a Bruker Avance III HD 800 MHz spectrometer (^{13}C NMR at 200 MHz). Chemical shifts (δ) are reported in parts per million (ppm) with reference to solvent signals [^{1}H-NMR: DMSO-*d*$_6$ (2.50 ppm); ^{13}C-NMR: DMSO-*d*$_6$ (39.51 ppm)]. Signal patterns are reported as s (singlet), d (doublet), t (triplet), q (quartet), h (heptet), m (multiplet) and br (broad). Coupling constants (*J*) are given in Hz. HRMS measurements were performed using ThermoFisher Scientific Q Exactive instrument. Abbreviations presented in experimental procedures are referred to the following definitions: DIEA, *N,N*-diisopropylethylamine; DME, 1,2-dimethoxyethane; DMF, *N,N*-dimethylformamide; EtOAc, ethyl acetate; HATU, *O*-(7-azabenzotriazol-1-yl)-*N,N,N',N'*-tetramethyluronium hexafluorophosphate; $NaOH_{(aq)}$, aqueous sodium hydroxide solution; $Na_2CO_{3(sat)}$, saturated aqueous sodium carbonate solution; Na_2SO_4, sodium sulfate.

1b. Typical method for the preparation of compounds 2a

To a solution of 2-((((9H-fluoren-9-yl) methoxy) carbonyl)amino)-3-(1-(tert butoxycarbonyl) piperidin-4-yl)propanoic acid (1) (494.58 mg, 1 mmol, 1 equiv.), methylamine HCl (202.5 mg, 3 equiv.), and HATU (1140 mg, 3 mmol, 3 equiv.) in anhydrous DMF (10 mL) was added DIEA (522 µL, 3 mmol, 3 equiv.) under nitrogen. The reaction mixture was stirred at room temperature for 1 h and then quenched by the addition of water. The aqueous layer was extracted twice with EtOAc, and the combined organic extracts were washed with brine, dried over anhydrous Na_2SO_4, filtered and concentrated. The residue was purified by column chromatography on silica gel (CH_3OH/CH_2Cl_2, 1:99 to 5:95) to afford N1-(2-methoxy-5-nitrophenyl)-N4-methylterephthalamide (507 mg, 98%) as a sticky oil.

The compounds (**2a**–**2c**) were prepared in a way similar to the procedure described above.

1c.1. Synthesis of intermediate N1-(2-methoxy-5-nitrophenyl)-N4-methylterephthalamide (2a):

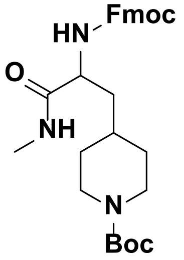

Sticky oil ; yield: 98%^{1}H NMR (600 MHz, CD_3OD) δ 7.80 (d, *J* = 7.5 Hz, 2H), 7.66 (t, *J* = 7.9 Hz, 2H), 7.39 (t, *J* = 7.3 Hz, 2H), 7.31 (t, *J* = 7.4 Hz, 2H), 4.47 (dd, *J* = 10.6, 6.7 Hz, 1H), 4.37 (dd, *J* = 10.6, 6.5 Hz, 1H), 4.21 (t, *J* = 6.5 Hz, 1H), 4.11 (dd, *J* = 9.9, 4.9 Hz, 1H), 4.04−4.02 (m, 2H), 2.72−2.65 (m, 5H),

1.72−1.47 (m, 5H), 1.44 (s, 9H), 1.13−1.00 (m, 2H); HRMS (ESI) m/z calcd for $C_{29}H_{37}N_3O_5$ [M + H]+ 508.2806, found 508.2895.

Tert-butyl (R)-4-(2-((((9H-fluoren-9-yl) methoxy)carbonyl)amino)-3-(methylamino)-3oxopropyl) piperidine-1-carboxylate (2b) :

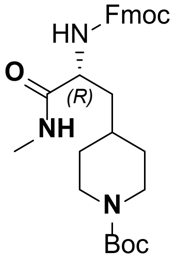

White solid; yield: 98%; ^{1}H NMR (600 MHz, CD_3OD) δ 7.80 (d, J = 7.5 Hz, 2H), 7.66 (t, J = 7.9 Hz, 2H), 7.39 (t, J = 7.4 Hz, 2H), 7.31 (t, J = 7.4 Hz, 2H), 4.48 (dd, J = 10.6, 6.8 Hz, 1H), 4.37 (dd, J = 10.6, 6.5 Hz, 1H), 4.22 (t, J = 6.5 Hz, 1H), 4.11 (dd, J = 9.9, 4.9 Hz, 1H), 4.04−4.02 (m, 2H), 2.71−2.65 (m, 5H), 1.73−1.47 (m, 5H), 1.44 (s, 9H), 1.13−1.00 (m, 2H); HRMS (ESI) m/z calcd for $C_{29}H_{37}N_3O_5$ [M + H]+ 508.2806, found 508.2794.; [α]25/D +2.7±1°, c = 1% in DMF

Tert-butyl (S)-4-(2-((((9H-fluoren-9-yl) methoxy) carbonyl)amino)-3-(methylamino)-3oxopropyl) piperidine-1-carboxylate (2c) :

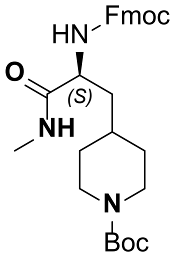

Sticky oil; yield: 98%; ^{1}H NMR (600 MHz, CD_3OD) δ 7.80 (d, J = 7.5 Hz, 2H), 7.66 (t, J = 8.0 Hz, 2H), 7.39 (t, J = 7.4 Hz, 2H), 7.31 (t, J = 7.4 Hz, 2H), 4.48 (dd, J = 10.6, 6.8 Hz, 1H), 4.37 (dd, J = 10.6, 6.4 Hz, 1H), 4.22 (t, J = 6.5 Hz, 1H), 4.11 (dd, J = 9.9, 4.9 Hz, 1H), 4.04−4.02 (m, 2H), 2.71−2.65 (m, 5H), 1.72−1.47 (m, 5H), 1.44 (s, 9H), 1.13−1.00 (m, 2H); HRMS (ESI) m/z calcd for $C_{29}H_{37}N_3O_5$ [M + H]+ 508.2806, found 508.2800.; [α]25/D -2.3±1°, c = 1% in DMF

1c.2 Typical method for the preparation of compound 4a

To a mixture of **2a** (407 mg, 1 mmol, 0.6 eq) in dioxane (20 mL) was added HCl/dioxane (4 M, 4 mL, 1.5 eq.) dropwise via syringe at 20 °C, and the resulting reaction mixture stirred for 1 h. After completion of the reaction all solvents were removed in vacuo and kept overnight dry in high vacuo. To the same vessel mixture of DIEA (522 μL, 3 mmol, 2 eq), 2-(4-methyl-2-oxo-1,2-dihydroquinolin-6-yl) acetic acid (3a) (325 mg, 1.5 mmol, 1 eq) and HATU (1140 mg, 3 mmol, 3 equiv.) added in the solution of anhydrous DMF (10 ml) under nitrogen and stir for 12h. The aqueous layer was extracted twice with EtOAc, and the combined organic extracts were washed with brine, dried over anhydrous Na_2SO_4, filtered and concentrated. The residue was purified by column chromatography on silica gel (CH_3OH/CH_2Cl_2, 1:99 to 5:95) to afford 4a (510 mg, 84%) as a white solid.

The compounds (**4a**–**4d**) were prepared in a way similar to the procedure described above.

(9H-fluoren-9-yl) methyl (3-(1-(2-(4-methyl-2-oxo-1,2-dihydroquinolin-6-yl) acetyl) piperidin-4-yl)-1-(methylamino)-1-oxopropan-2-yl) carbamate (4a):

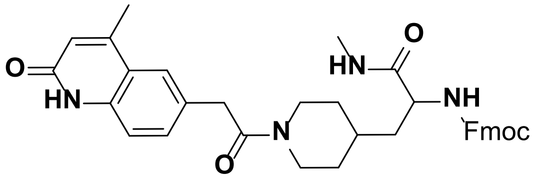

White solid; yield: 84%; ^1H NMR (600 MHz, DMSO-d_6) δ 11.57 (s, 1H), 7.89 (d, *J* = 7.5 Hz, 2H), 7.85−7.84 (m, 1H), 7.72 (t, *J* = 6.6 Hz, 2H), 7.53 (s, 1H), 7.51 (t, *J* = 8.7 Hz, 1H), 7.42−7.40 (m, 2H), 7.36−7.30 (m, 3H), 7.24 (d, *J* = 8.3 Hz, 1H), 6.39 (s, 1H), 6.28 (s, 1H), 4.36−4.28 (m, 2H), 4.25−4.20 (m, 2H), 3.99−3.96 (m, 2H), 3.76 (brs, 2H), 2.97−2.87 (m, 1H), 2.59−2.54 (m, 4H), 2.39 (s, 3H), 1.66−1.59 (m, 2H), 1.51−1.43 (m, 2H), 0.99-0.85 (m, 2H); HRMS (ESI) m/z calcd for $C_{36}H_{38}N_4O_5$ [M + H]+ 607.2915, found 607.2903.

(9H-fluoren-9-yl) methyl (R)-(3-(1-(2-(4-methyl-2-oxo-1,2-dihydro quinolin-6-yl) acetyl) piperidin-4-yl)-1-(methylamino)-1-oxopropan-2-yl) carbamate (4b):

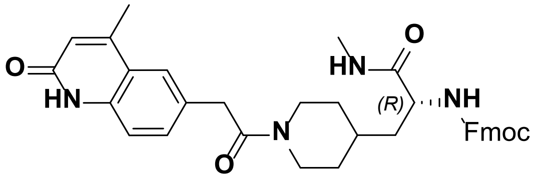

White solid; yield: 84%; ^1H NMR (600 MHz, DMSO-d_6) δ 11.57 (s, 1H), 7.89 (d, *J* = 7.5 Hz, 2H), 7.85−7.83 (m, 1H), 7.72 (t, *J* = 6.6 Hz, 2H), 7.53 (s, 1H), 7.50 (t, *J* = 8.7 Hz, 1H), 7.43−7.40 (m, 2H), 7.36−7.30 (m, 3H), 7.24 (d, *J* = 8.3 Hz, 1H), 6.39 (s, 1H), 6.28 (s, 1H), 4.36−4.30 (m, 2H), 4.25−4.20 (m, 2H), 3.98−3.96 (m, 2H), 3.76 (brs, 2H), 2.97−2.87 (m, 1H), 2.58−2.54 (m, 4H), 2.39 (s, 3H), 1.66−1.59 (m, 2H), 1.51−1.40 (m, 2H), 0.97-0.84 (m, 2H) ; HRMS (ESI) m/z calcd for $C_{36}H_{38}N_4O_5$ [M + H]+ 607.2915, found 607.2912; [α]25/D +1.7±1°, c = 1% in DMF

(9H-fluoren-9-yl) methyl (S)-(3-(1-(2-(4-methyl-2-oxo-1,2-dihydro quinolin-6-yl) acetyl) piperidin-4-yl)-1-(methylamino)-1-oxopropan-2-yl) carbamate (4c):

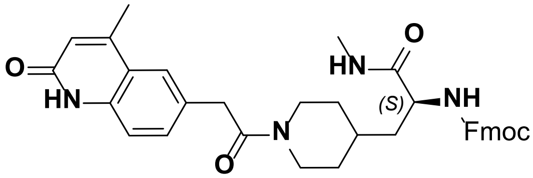

Sticky oil; yield: 84%; ^1H NMR (600 MHz, DMSO-d_6) δ 11.57 (s, 1H), 7.89 (d, *J* = 7.5 Hz, 2H), 7.84−7.83 (m, 1H), 7.72 (t, *J* = 6.6 Hz, 2H), 7.53 (s, 1H), 7.50 (t, *J* = 8.7 Hz, 1H), 7.42−7.39 (m, 2H), 7.35−7.30 (m, 3H), 7.24 (d, *J* = 8.3 Hz, 1H), 6.38 (s, 1H), 6.27 (s, 1H), 4.35−4.29 (m, 2H), 4.24−4.19 (m, 2H), 3.99−3.93 (m, 2H), 3.75 (brs, 2H), 2.96−2.86 (m, 1H), 2.57−2.55 (m, 4H), 2.38 (s, 3H), 1.67−1.60 (m, 2H), 1.50−1.38 (m, 2H), 0.96-0.84 (m, 2H) ; HRMS (ESI) m/z calcd for $C_{36}H_{38}N_4O_5$ [M + H]+ 607.2915, found 607.2902; [α]25/D -1.0±1°, c = 1% in DMF

(9H-fluoren-9-yl) methyl (R)-(1-(methylamino)-1-oxo-3-(1-(2-(2-oxo-1,2-dihydroquinolin-6-yl) acetyl) piperidin-4-yl) propan-2-yl) carbamate(4d):

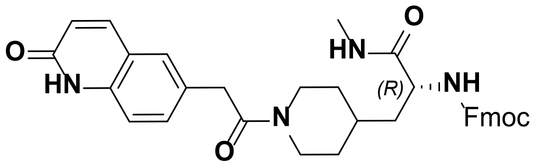

White solid; yield: 84%; ^{1}H NMR (600 MHz, CD_3OD) δ 7.88−7.87 (m, 1H), 7.75 (d, *J* = 7.1 Hz, 2H), 7.61 (brs, 2H), 7.49 (brs, 1H), 7.42 (d, *J* = 6.6 Hz, 2H), 7.37−7.26 (m, 5H), 6.6 (m, 1H), 4.52−4.44 (m, 2H), 4.37−4.33 (m, 1H), 4.18 (t, *J* = 6.4 Hz, 1H), 4.11−4.08 (m, 1H), 3.96 (brs, 1H), 3.80 (s, 2H), 3.03−2.93 (m, 1H), 2.70 (s, 3H), 2.63−2.55 (m, 1H), 1.78−1.52 (m, 5H), 1.10−0.87 (m, 2H); HRMS (ESI) m/z calcd for $C_{35}H_{36}N_4O_5$ [M + H]+ 593.2758, found 593.2754; [α]25/D +1.3±1°, c = 1% in DMF

1c.3 Typical method for the preparation of compounds 6a

Compound 4a (322 mg, 0.84 mmol, 1 eq) was dissolved in DMF (3 ml) containing piperidine (10% v/v) and stirred 1h in room temperature. Resulting mixture is evaporated to The SM was dissolved in DMF containing piperidine, and all solvents were removed after 20 min. After completing reaction all solvents were removed in vacuo and kept overnight dry in high vacuo. To the same vessel mixture of DIEA (439 µL, 2.5 mmol, 3 eq), 5-(2,4-dimethylphenyl) picolinic acid (5a) (140 mg, 0.7 mmol, 0.8 eq) and HATU (957.6 mg, 2.5 mmol, 3 equiv.) added in the solution of anhydrous DMF (8 ml) under nitrogen. The aqueous layer was extracted twice with EtOAc, and the combined organic extracts were washed with brine, dried over anhydrous Na_2SO_4, filtered and concentrated. The residue was purified by column chromatography on silica gel (CH_3OH/CH_2Cl_2, 1:99 to 5:95) to afford 4a (327 mg, 68%) as a white solid.

The compounds (**6a**–**6o**) were prepared in a way similar to the procedure described above.

Synthesis of 5-(2,4-dimethylphenyl)-N-(3-(1-(2-(4-methyl-2-oxo-1,2-dihydroquinolin-6-yl) acetyl) piperidin-4-yl)-1-(methylamino)-1-oxopropan-2-yl) picolinamide (6a): CDD-724

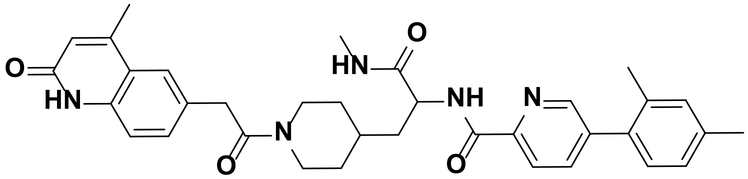

White solid; yield: 60%; ^{1}H NMR (600 MHz, DMSO-d_6) δ 11.56 (s, 1H), 8.66 (t, *J* = 9.4 Hz, 1H), 8.63 (s, 1H), 8.10−8.08 (m, 2H), 7.98 (dd, *J* = 7.9, 2.1 Hz, 1H), 7.53 (brs, 1H), 7.34 (d, *J* = 7.9 Hz, 1H), 7.23 (d, *J* = 8.3 Hz, 1H), 7.18−7.17 (m, 2H), 7.14 (d, *J* = 7.8 Hz, 1H), 6.38 (s, 1H), 4.58−4.56 (m, 1H), 4.36−4.31 (m, 1H), 3.99−3.93 (m, 1H), 3.75 (s, 2H), 2.97−2.91 (m, 1H), 2.6 (d, *J* = 4.5 Hz, 2H), 2.50−2.46 (m, 1H), 2.39 (d, *J* = 3.1 Hz, 3H), 2.33 (s, 3H), 2.23 (s, 3H), 1.8 (brs, 1H), 1.69−1.52 (m, 4H), 0.97−0.94 (m, 2H); 13C NMR (150 MHz, DMSO-d6) δ 171.8, 168.4, 163.1, 161.5, 148.3, 147.6, 147.6, 139.6, 138.0, 137.8, 137.2, 134.9, 134.0, 131.4, 131.3, 129.7, 129.4, 126.9, 124.7, 121.5, 120.9, 119.4, 115.3, 50.3, 45.4, 41.3, 32.6, 32.1, 31.9, 31.5, 30.9, 25.6, 20.7, 19.9, 18.5; HRMS (ESI) m/z calcd for $C_{35}H_{39}N_5O_4$ [M + H]+ 594.3075, found 594.3068.

(R)-5-(2,4-dimethylphenyl)-N-(3-(1-(2-(4-methyl-2-oxo-1,2-dihydroquinolin-6-yl) acetyl) piperidin-4-yl)-1-(methylamino)-1-oxopropan-2-yl) picolinamide (6b): CDD-787

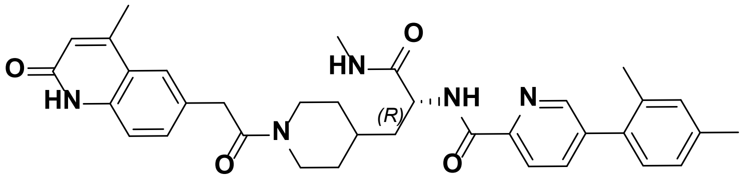

White solid; yield: 60%; ^{1}H NMR (600 MHz, DMSO-d_6) δ 11.56 (s, 1H), 8.66 (t, J = 9.4 Hz, 1H), 8.63 (s, 1H), 8.10−8.08 (m, 2H), 7.98 (dd, J = 7.9, 2.1 Hz, 1H), 7.53 (brs, 1H), 7.34 (d, J = 7.9 Hz, 1H), 7.23 (d, J = 8.3, Hz, 1H), 7.19−7.18 (m, 2H), 7.14 (d, J = 7.8 Hz, 1H), 6.38 (s, 1H), 4.58−4.56 (m, 1H), 4.36−4.31 (m, 1H), 3.99−3.93 (m, 1H), 3.75 (s, 2H), 2.98−2.91 (m, 1H), 2.6 (d, J = 4.5 Hz, 2H), 2.50−2.46 (m, 1H), 2.39 (d, J = 3.1 Hz, 3H), 2.33 (s, 3H), 2.23 (s, 3H), 1.8 (brs, 1H), 1.71−1.52 (m, 4H), 0.99−0.95 (m, 2H); 13C NMR (150 MHz, DMSO-d6) δ 171.8, 168.5, 163.2, 161.5, 148.4, 147.7, 139.6, 138.0, 137.8, 137.2, 134.9, 134.0, 131.3, 129.7, 129.4, 126.9, 124.7, 121.5, 120.9, 119.4, 115.3, 50.3, 45.4, 41.3, 32.6, 32.1, 31.9, 31.6, 30.9, 25.6, 20.7, 19.9, 18.5; HRMS (ESI) m/z calcd for $C_{35}H_{39}N_5O_4$ [M + H]+ 594.3075, found 594.3072; [α]25/D +16.2±1°, c = 1% in DMF

(S)-5-(2,4-dimethylphenyl)-N-(3-(1-(2-(4-methyl-2-oxo-1,2-dihydroquinolin-6-yl) acetyl) piperidin-4-yl)-1-(methylamino)-1-oxopropan-2-yl)picolinamide (6c): CDD-786

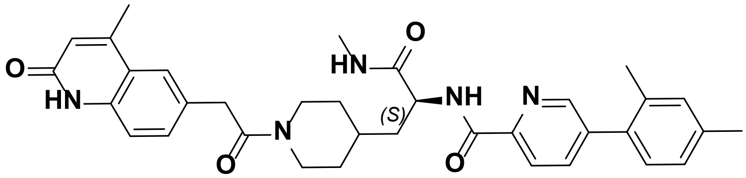

White solid; yield: 60%; ^{1}H NMR (600 MHz, DMSO-d_6) δ 11.55 (s, 1H), 8.66 (t, J = 9.4 Hz, 1H), 8.63 (s, 1H), 8.09−8.08 (m, 2H), 7.98 (dd, J = 7.9, 2.1 Hz, 1H), 7.53 (brs, 1H), 7.34 (d, J = 7.9 Hz, 1H), 7.23 (d, J = 8.3, Hz, 1H), 7.18−7.17 (m, 2H), 7.13 (d, J = 7.8 Hz, 1H), 6.38 (s, 1H), 4.59−4.54 (m, 1H), 4.36−4.31 (m, 1H), 3.98−3.92 (m, 1H), 3.75 (s, 2H), 2.98−2.89 (m, 1H), 2.59 (d, J = 4.5 Hz, 2H), 2.54−2.46 (m, 1H), 2.38 (d, J = 3.1 Hz, 3H), 2.33 (s, 3H), 2.22 (s, 3H), 1.8 (brs, 1H), 1.72−1.52 (m, 4H), 0.99−0.93 (m, 2H); 13C NMR (150 MHz, DMSO-d6) δ 171.8, 168.4, 163.1, 161.5, 148.3, 147.6, 147.5, 139.6, 138.0, 137.8, 137.2, 134.9, 134.0, 131.4, 131.3, 129.7, 129.4, 126.9, 124.7, 121.5, 120.9, 119.4, 115.3, 50.3, 45.4, 41.3, 32.6, 32.1, 31.9, 31.5, 30.9, 25.6, 20.7, 19.9, 18.5; HRMS (ESI) m/z calcd for $C_{35}H_{39}N_5O_4$ [M + H]+ 594.3075, found 594.3073; [α]25/D -16.7±1°, c = 1% in DMF

(R)-N-(3-(1-(2-(4-methyl-2-oxo-1,2-dihydroquinolin-6-yl)acetyl)piperidin-4-yl)-1-(methylamino)-1-oxopropan-2-yl)-5-phenylpicolinamide (6d): CDD-956

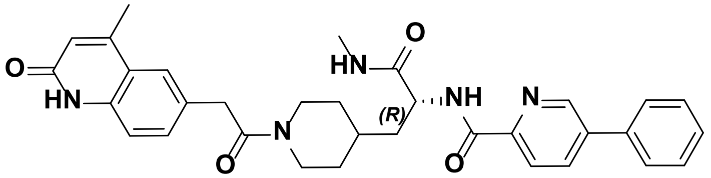

White solid; yield: 60%; ^{1}H NMR (600 MHz, DMSO-d_6) δ 11.54 (s, 1H), 8.98 (s, 1H), 8.65 (t, J = 9.0 Hz, 1H), 8.29 (dd, J = 8.1, 2.1 Hz, 1H), 8.12−8.09 (m, 2H), 7.80 (d, J = 7.3 Hz, 2H), 7.56−7.53 (m, 3H), 7.4

(t, J = 7.3 Hz, 1H), 7.34 (d, J = 8.3 Hz, 1H), 7.23 (d, J = 8.3 Hz, 1H), 6.38 (s, 1H), 4.61−4.56 (m, 1H), 4.36−4.32 (m, 1H), 3.98−3.93 (m, 1H), 3.77−3.72 (m, 2H), 2.97−2.89 (m, 1H), 2.61 (d, J = 4.5 Hz, 1H), 2.54−2.46 (m, 1H), 2.38 (d, J = 2.5 Hz, 3H), 1.80 (s, 1H), 1.73−1.52 (m, 4H), 0.99−0.93 (m, 2H); 13C NMR (150 MHz, DMSO-d6) δ 171.7, 168.4, 163.0, 161.4, 148.1, 147.5, 146.5, 138.1, 137.1, 136.1, 135.5, 131.2, 129.3, 129.2, 128.7, 127.1, 124.6, 122.0, 120.8, 119.3, 115.2, 50.2, 45.3, 41.2, 32.5, 32.0, 31.8, 31.5, 30.9, 25.5, 18.4; HRMS (ESI) m/z calcd for $C_{33}H_{35}N_5O_4$ [M + H]+ 566.2762, found 566.2762; [α]25/D +19.1±1°, c = 1% in DMF

(S)-N-(3-(1-(2-(4-methyl-2-oxo-1,2-dihydroquinolin-6-yl) acetyl) piperidin-4-yl)-1-(methylamino)-1-oxo propan-2-yl)-5-phenylpicolinamide (6e): CDD-2107

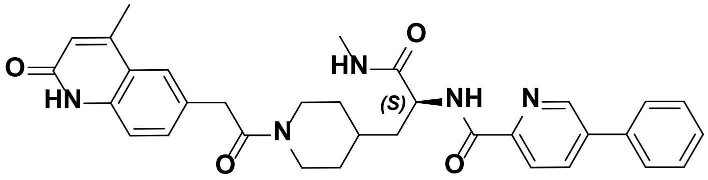

White solid; yield: 55%; ^{1}H NMR (600 MHz, CD_3OD) δ 8.88 (s, 1H), 8.18 (dd, J = 8.0, 1.5 Hz, 1H), 8.14−8.12 (m, 1H), 7.69 (d, J = 7.3 Hz, 2H), 7.66 (s, 1H), 7.51 (t, J = 7.5 Hz, 2H), 7.44 (t, J = 8.3 Hz, 2H), 7.31 (d, J = 8.4 Hz, 1H), 6.49 (s, 1H), 4.69−4.64 (m, 1H), 4.52−4.51 (m, 1H), 4.05−4.03 (m, 1H), 3.89−3.85 (m, 2H), 3.07−2.99 (m, 1H), 2.65−2.57 (m, 1H), 2.49 (s, 3H), 1.92−1.65 (m, 5H), 1.18−0.95 (m, 2H); 13C NMR (150 MHz, CD_3OD) δ 174.6, 171.6, 166.2, 164.8, 151.4, 149.1, 148.0, 138.2, 137.9, 136.8, 132.8, 131.5, 130.3, 129.9, 128.3, 125.9, 125.9, 125.9, 123.4, 121.9, 120.9, 117.4, 52.3, 47.5, 43.4, 40.6, 33.9, 33.3, 32.9, 32.3, 26.4, 19.1; HRMS (ESI) m/z calcd for $C_{33}H_{35}N_5O_4$ [M + H]+ 566.2762, found 566.2748; [α]25/D -17.7±1°, c = 1% in DMF

(R)-5-(4-chlorophenyl)-N-(3-(1-(2-(4-methyl-2-oxo-1,2-dihydroquinolin-6-yl)acetyl)piperidin-4-yl)-1 (methylamino)-1-oxopropan-2-yl) picolinamide (6f): CDD-981

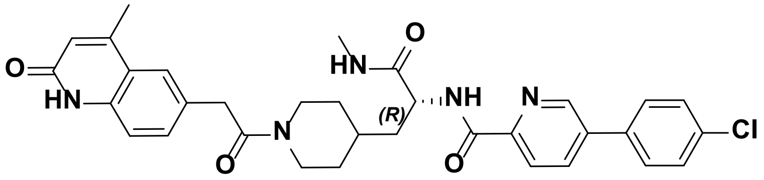

White solid; yield: 55%; ^{1}H NMR (600 MHz, CD_3OD) δ 8.82 (s, 1H), 8.14−8.09 (m, 2H), 7.65 (d, , J = 8.5 Hz, 2H), 7.61 (s, 1H), 7.47 (d, , J = 8.5 Hz, 2H), 7.39 (d, , J = 8.4 Hz, 1H), 7.26 (d, , J = 8.4 Hz, 1H), 6.45 (s, 1H), 4.69−4.64 (m, 1H), 4.53−4.50 (m, 1H), 4.04−4.02 (m, 1H), 3.87−3.80 (m, 2H), 3.05−2.97 (m, 1H), 2.74 (s, 3H), 2.64−2.56 (m, 1H), 2.45 (s, 3H), 1.91−1.64 (m, 5H), 1.16−0.95 (m, 2H); 13C NMR (150 MHz, CD_3OD) δ 174.6, 171.5, 166.0, 164.7, 151.2, 149.4, 147.9, 139.4, 138.1, 136.6, 136.5, 136.1, 132.7, 131.3, 130.4, 129.8, 125.9, 123.4, 121.8, 121.0, 117.3, 52.3, 47.4, 43.3, 40.6, 40.2, 33.9, 33.3, 32.9, 32.3, 26.4, 19.0; HRMS (ESI) m/z calcd for $C_{33}H_{35}ClN_5O_4$ [M + H]+ 600.2372, found 600.2343; [α]25/D +16.5±1°, c = 1% in DMF

(R)-5-(4-chloro-3-fluorophenyl)-N-(3-(1-(2-(4-methyl-2-oxo-1,2-dihydroquinolin-6-yl)acetyl)piperidin-4-yl)-1-(methylamino)-1-oxopropan-2-yl) picolinamide (6g): CDD-982

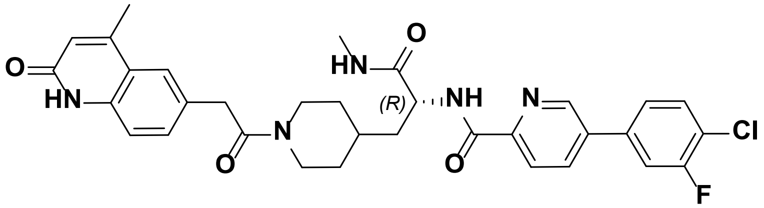

White solid; yield: 58%; ^{1}H NMR (600 MHz, CD_3OD) δ 8.87 (s, 1H), 8.18 (dd, , *J* = 8.1, 1.0 Hz, 1H), 8.13−8.11 (m, 1H), 7.63−7.61 (m, 2H), 7.58 (t, *J* = 7.9 Hz, 1H), 7.51 (dd, *J* = 8.3, 1.4 Hz, 1H), 7.41 (d, *J* = 8.4 Hz, 1H), 7.28 (d, *J* = 8.4 Hz, 1H), 6.4 (brs, 1H), 4.69−4.64 (m, 1H), 4.53−4.51 (m, 1H), 4.05−4.03 (m, 1H), 3.88−3.81 (m, 2H), 3.06−2.99 (m, 1H), 2.74 (s, 3H), 2.64−2.57 (m, 1H), 2.47 (s, 3H), 1.92−1.65(m, 5H), 1.17−0.96 (m, 2H); 13C NMR (150 MHz, CD_3OD) δ 174.6, 171.6, 165.9, 164.8, 160.6, 158.9, 151.2, 149.8, 147.9, 138.8, 138.2, 136.8, 132.5, 131.4, 125.9, 125.0, 123.4, 122.4, 121.8, 121.0, 117.3, 116.5, 52.3, 43.4, 40.6, 40.2, 33.9, 33.3, 33.0, 32.3, 26.4, 19.0; HRMS (ESI) m/z calcd for $C_{33}H_{33}ClFN_5O_4$ [M + H]+ 618.2278, found 618.2275; [α]25/D +15.8±1°, c = 1% in DMF

(R)-N-(1-(methylamino)-1-oxo-3-(1-(2-(2-oxo-1,2-dihydroquinolin-6-yl)acetyl)piperidin-4-yl)propan-2-yl)-5-phenylpicolinamide (6h): CDD-986

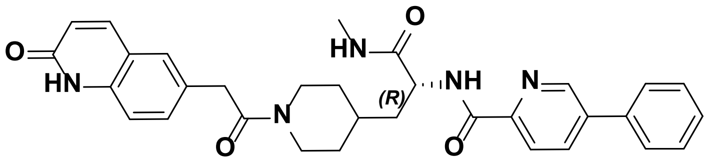

White solid; yield: 58%; ^{1}H NMR (600 MHz, CD_3OD) δ 8.88 (s, 1H), 8.19−8.12 (m, 2H), 7.9 (d, , *J* = 8.4 Hz, 1H), 7.69 (d, , *J* = 7.3 Hz, 2H), 7.52−7.49 (m, 3H), 7.45−7.42 (m, 2H), 7.31 (d, *J* = 9.0 Hz, 1H), 6.59 (d, *J* = 9.4 Hz, 1H), 4.69−4.64 (m, 1H), 4.53−4.47 (m, 1H), 4.01 (t, *J* = 8.3 Hz, 1H), 3.82 (s, 2H), 3.06−2.97 (m, 1H), 2.74 (s, 3H), 2.61−2.54 (m, 1H), 1.92−1.64 (m, 5H), 1.15−0.96 (m, 2H); 13C NMR (150 MHz, CD_3OD) δ 174, 171.4, 166.2, 165.1, 149.1, 148.0, 142.5, 140.9, 138.5, 137.9, 136.7, 132.8, 131.5, 130.3, 129.0, 128.3, 123.4, 122.0, 121.4, 116.9, 52.3, 47.5, 43.3, 40.5, 40.3, 33.9, 33.2, 32.9, 32.2, 26.4; HRMS (ESI) m/z calcd for $C_{32}H_{33}N_5O_4$ [M + H]+ 552.2605, found 552.2601; [α]25/D +10.7±1°, c = 1% in DMF

N-(3-(1-(2-(4-methyl-2-oxo-1,2-dihydroquinolin-6-yl)acetyl)piperidin-4-yl)-1-(methylamino)-1-oxopropan-2-yl)picolinamide (6i): CDD-784

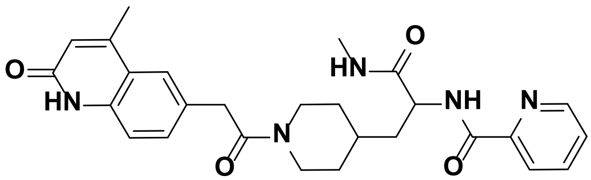

White solid; yield: 72%; ^{1}H NMR (600 MHz, DMSO-d_6) δ 11.58 (s, 1H), 8.67 (d, *J* = 4.5 Hz, 1H), 8.63 (t, *J* = 8.5 Hz, 1H), 8.08−8.00 (m, 3H), 7.64−7.62 (m, 1H), 7.53(s, 1H), 7.34 (d, *J* = 8.3 Hz, 1H), 7.24 (d, *J* = 8.3 Hz, 1H), 6.38 (brs, 1H), 4.56−4.53 (m, 1H), 4.35−4.30 (m, 1H), 3.97 −3.92 (m, 1H), 3.74 (s, 2H), 2.96−2.88 (m, 1H), 2.59 (d, *J* = 4.5 Hz, 3H), 2.54 (s, 2H), 2.39 (brs, 3H), 1.78−1.76 (m, 1H), 1.69−1.50 (m, 4H), 1.01−0.90 (m, 2H); 13C NMR (150 MHz, DMSO-d_6) δ 172.2, 168.9, 162.0, 149.7, 148.9, 138.4, 137.6, 131.8, 129.9, 127.2, 125.1, 122.4, 121.2, 119.9, 116.6, 50.7, 45.8, 41.7, 40.9, 33.0,

32.5, 32.3, 32.0, 31.4, 26.0, 18.9; HRMS (ESI) m/z calcd for $C_{27}H_{31}N_5O_4$ [M + H]+ 490.2449, found 490.2451.

N-(3-(1-(2-(4-methyl-2-oxo-1,2-dihydroquinolin-6-yl)acetyl)piperidin-4-yl)-1-(methylamino)-1-oxopropan-2-yl)-5-phenylpicolinamide (6j): CDD-1146

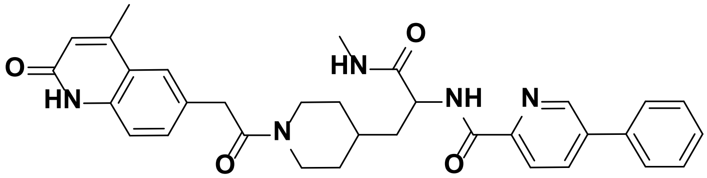

White solid; yield: 62%; ^{1}H NMR (600 MHz, DMSO-d_6) δ 11.56 (s, 1H), 8.98 (s, 1H), 8.66 (t, J = 8.8 Hz, 1H), 8.29−8.27 (m, 1H), 8.12−8.10 (m, 2H), 7.8 (d, J = 7.5 Hz, 2H), 7.55−7.53 (m, 3H), 7.49−7.46 (m, 1H), 7.34 (d, J = 8.3 Hz, 1H), 7.24 (d, J = 8.3 Hz, 1H), 6.38 (s, 1H), 4.60−4.58 (m, 1H), 4.36−4.32 (m, 1H), 3.98−3.93 (m, 1H), 3.74 (s, 2H), 2.97−2.88 (m, 1H), 2.61 (d, J = 4.5 Hz, 3H), 2.5−2.46 (m, 1H), 2.38 (brs, 3H), 1.80 (brs, 1H), 1.73−1.52 (m, 4H), 1.02−0.92 (m, 2H); 13C NMR (150 MHz, DMSO-d_6) δ 172.2, 168.9, 163.5, 162.0, 148.6, 148.0, 147.0, 138.6, 137.6, 136.6, 136.0, 131.7, 131.8, 129.9, 129.7, 129.2, 127.6, 125.1, 122.5, 121.3, 119.9, 115.8, 50.7, 45.8, 41.7, 40.9, 33.0, 32.6, 32.3, 32.1, 31.4, 26.0, 18.9; HRMS (ESI) m/z calcd for $C_{33}H_{35}N_5O_4$ [M + H]+ 566.2762, found 566.2766.

1c.4. Typical method for the preparation of compounds 8 tert-butyl 4-(2-(5-(2,4-dimethylphenyl) picolinamido) propyl) piperidine-1-carboxylate (8a):

To a solution of tert-butyl 5-(2,4-dimethylphenyl) picolinic acid (**5a**) (228 mg, 1mmol, 1equal.), tert-butyl 4-(2-aminopropyl) piperidine-1-carboxylate (7a) (242 mg, 1mmol, 1equal.), and HATU (570 mg, 1.5 mmol, 1.5 equiv.) in anhydrous DMF (5 mL) was added DIEA (175 µL, 1.5 mmol, 1.5 equiv.) under nitrogen. The reaction mixture was stirred at room temperature for 3 h and then quenched by the addition of water. The aqueous layer was extracted twice with EtOAc, and the combined organic extracts were washed with brine, dried over anhydrous Na_2SO_4, filtered and concentrated. The residue was purified by column chromatography on silica gel (CH_3OH/CH_2Cl_2, 1:99 to 5:95) to afford tert-butyl 4-(2-(5-(2,4-dimethylphenyl) picolinamido) propyl) piperidine-1-carboxylate (507 mg, 98%) as a sticky solid.

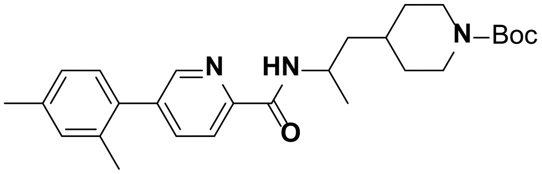

Sticky solid; yield: 98%; ^{1}H NMR (600 MHz, DMSO-d_6) δ 8.58 (d, J = 1.5 Hz, 1H), 8.51 (d, J = 9.1 Hz, 1H), 8.08 (d, J = 8.4 Hz, 1H), 7.95 (dd, J = 8.0, 2.2 Hz, 1H), 7.17−7.16 (m, 2H), 7.13 (d, J = 7.8 Hz, 1H), 4.21−4.16 (m, 1H), 3.89 (s, 2H), 2.33 (s, 3H), 2.22 (s, 3H), 1.77−1.75 (m, 1H), 1.67−1.62 (m, 1H), 1.59−1.57 (m, 1H), 1.48−1.42 (m, 1H), 1.37−1.31 (m, 11H), 1.17 (d, J = 6.5 Hz, 3H), 1.04−1.57 (m, 2H); HRMS (ESI) m/z calcd for $C_{27}H_{37}N_3O_3$ [M + H]+ 452.2908, found 452.2912.

1c.5 Typical method for the preparation of compound 5-(2,4-dimethylphenyl)-N-(1-(1-(2-(4-methyl-2-oxo-1,2-dihydroquinolin-6-yl) acetyl) piperidin-4-yl) propan-2-yl)picolinamide (9a): CDD-906

To a mixture of **8a (**451 mg, 1mmol, 1 eq), in dioxane (20 mL) was added HCl/dioxane (4 M, 4 mL, 1.5 eq.) dropwise via syringe at 20 °C, and the resulting reaction mixture stirred for 1 h. After completion of

the reaction all solvents were removed in vacuo and kept overnight dry in high vacuo. To the same vessel mixture of DIEA (263 µL, 1.5 mmol, 1.5 eq), 2-(4-methyl-2-oxo-1,2-dihydroquinolin-6-yl) acetic acid (3a) (217 mg, 1mmol, 1 eq), and HATU (570 mg, 1.5 mmol, 1.5 equiv.) added in the solution of anhydrous DMF (10 ml) under nitrogen. The aqueous layer was extracted twice with EtOAc, and the combined organic extracts were washed with brine, dried over anhydrous Na_2SO_4, filtered and concentrated. The residue was purified by column chromatography on silica gel (CH_3OH/CH_2Cl_2, 1:99 to 5:95) to afford 9a (510 mg, 84%) as a white solid.

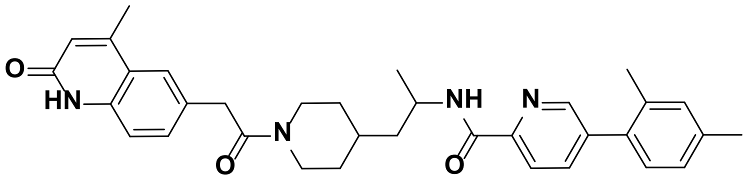

White solid; yield: 84%; ^{1}H NMR (600 MHz, DMSO-d_6) δ 11.57 (s, 1H), 8.55 (s, 1H), 8.51−8.49 (m, 1H), 8.08 (dd, *J* = 7.9, 3.1 Hz, 1H), 7.9 (dd, *J* = 7.9, 2.0 Hz, 1H), 7.5 (s, 1H), 7.33 (d, *J* = 8.3 Hz, 1H), 7.24 (d, *J* = 8.3 Hz, 1H), 7.13−7.12 (m, 2H), 7.08 (d, *J* = 7.9 Hz, 1H), 6.37 (s, 1H), 4.37−4.33 (m, 1H), 4.18−4.17 (m, 1H), 3.96−3.92 (m, 1H), 3.73 (s, 2H), 3.39 (s, 3H), 2.95−2.85 (m, 1H), 2.49−2.43 (m, 1H), 2.37 (d, 3.1 Hz, 3H), 2.29 (s, 3H), 2.18 (s, 3H), 1.80−1.78 (m, 1H), 1.63−1.57 (m, 2H), 1.51−1.50 (m, 1H), 1.29−1.27 (m, 1H), 1.15 (d, *J* = 6.3 Hz, 3H), 0.98−0.88 (m, 2H); 13C NMR (150 MHz, DMSO-d_6) δ 168.9, 164.4, 162.0, 150.7, 148.0, 146.4, 138.2, 137.6, 131.8, 130.8, 129.9, 129.6, 129.2, 128.5, 128.4, 125.1, 121.3, 119.9, 119.1, 115.8, 45.9, 41.8, 40.5, 39.6, 39.5, 36.9, 36.2, 33.4, 32.6, 31.9, 18.9 (2 x); HRMS (ESI) m/z calcd for $C_{34}H_{38}N_4O_3$ [M + H]+ 551.3017, found 551.3004.

1c.6 Typical method for the preparation of compound tert-butyl (2-(1-(2-(4-methyl-2-oxo-1,2-dihydroquinolin-6-yl) acetyl) piperidin-4-yl) ethyl) carbamate (11a):

To a solution of tert-butyl (2-(piperidin-4-yl) ethyl) carbamate (**10a**) (228 mg, 1 mmol, 1 eq.), 2-(4-methyl-2-oxo-1,2-dihydroquinolin-6-yl) acetic acid (**3a**) (217 mg, 1 mmol, 1 eq.), and HATU (570 mg, 1.5 mmol, 1.5 equiv.) in anhydrous DMF (5 mL) was added DIEA (175 µL, 1.5 mmol, 1.5 equiv.) under nitrogen. The reaction mixture was stirred at room temperature for 3 h and then quenched by the addition of water. The aqueous layer was extracted twice with EtOAc, and the combined organic extracts were washed with brine, dried over anhydrous Na_2SO_4, filtered and concentrated. The residue was purified by column chromatography on silica gel (CH_3OH/CH_2Cl_2, 1:99 to 5:95) to afford tert-butyl (2-(1-(2-(4-methyl-2-oxo-1,2-dihydroquinolin-6-yl) acetyl) piperidin-4-yl) ethyl) carbamate (**11a**) (507 mg, 98%) as a sticky oil.

The compounds (**11a–11b**) were prepared in a way similar to the procedure described above.

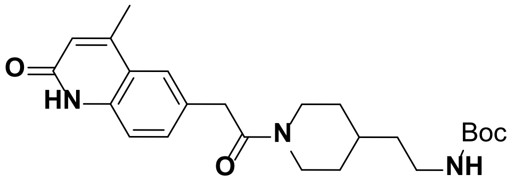

Sticky solid; yield: 84%; ^{1}H NMR (600 MHz, CD_3OD) δ 7.65 (s, 1H), 7.43 (dd, *J* = 8.4, 1.4 Hz, 1H), 7.29 (d, *J* = 8.4 Hz, 1H), 6.49 (s, 1H), 4.54−4.51 (m, 1H), 4.05−4.03 (m, 1H), 3.89−3.83 (m, 2H), 3.07−3.03

(m, 3H), 2.66−2.61 (m, 1H), 2.49 (s, 3H), 1.75−1.69 (m, 2H), 1.59−1.54 (m, 1H), 1.41 (s, 9H), 1.36−1.34 (m, 2H), 1.07−0.90 (m, 2H); HRMS (ESI) m/z calcd for $C_{24}H_{33}N_3O_4$ [M + H]+ 428.2544, found 428.2534.

tert-butyl (2-(4-(2-(4-methyl-2-oxo-1,2-dihydroquinolin-6-yl) acetyl) piperazin-1-yl)ethyl)carbamate (11b):

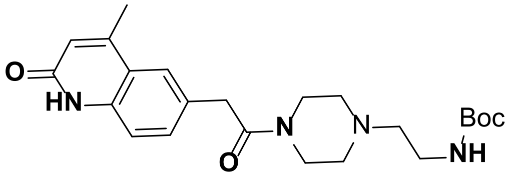

White solid; yield: 92%; ^{1}H NMR (600 MHz, DMSO-d_6) δ 11.56 (s, 1H), 7.58−7.53 (m, 1H), 7.40−7.39 (m, 1H), 7.34 (dd, J = 8.4, 1.5 Hz, 1H), 7.25 (t, J = 8.4 Hz, 1H), 6.65 (s, 1H), 6.38 (s, 1H), 3.76 (s, 2H), 3.64 (s, 1H), 3.49−3.45 (m, 4H), 2.40−2.39 (m, 4H), 2.33−2.31 (m, 6H), 1.36 (s, 9H); HRMS (ESI) m/z calcd for $C_{23}H_{32}N_4O_4$ [M + H]+ 429.2496, found 429.2495.

1c.7 Typical method for the preparation of compound 5-(2,4-dimethylphenyl)-N-(2-(1-(2-(4-methyl-2-oxo-1,2-dihydroquinolin-6-yl) acetyl)piperidin-4-yl)ethyl)picolinamide (12a):

Compound **11a (**536 mg, 1 mmol, 1 eq) was dissolved in DMF (3 ml) containing piperidine (10% v/v) and stirred 1h in room temperature. Resulting mixture is evaporated to The SM was dissolved in DMF containing piperidine, and all solvents were removed after 20 min. After completing reaction all solvents were removed in vacuo and kept overnight dry in high vacuo. To the same vessel mixture of DIEA (439 µL, 2.5 mmol, 3 eq), 5-(2,4-dimethylphenyl) picolinic acid (5a) (140 mg, 0.7 mmol, 0.8 eq) and HATU (957.6 mg, 2.5 mmol, 3 equiv.) added in the solution of anhydrous DMF (8 ml) under nitrogen. The aqueous layer was extracted twice with EtOAc, and the combined organic extracts were washed with brine, dried over anhydrous Na_2SO_4, filtered and concentrated. The residue was purified by column chromatography on silica gel (CH_3OH/CH_2Cl_2, 1:99 to 5:95) to afford **12a** (327 mg, 68%) as a white solid.

The compounds (**12a**–**12g**) were prepared in a way similar to the procedure described above.

5-(2,4-dimethylphenyl)-N-(2-(1-(2-(4-methyl-2-oxo-1,2-dihydroquinolin-6-yl) acetyl) piperidin-4-yl)ethyl)picolinamide (12a): CDD-813

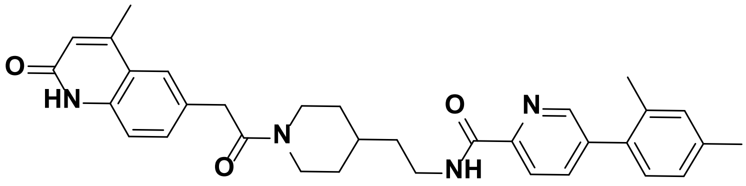

Sticky oil; yield: 84%; ^{1}H NMR (600 MHz, DMSO-d_6) δ 11.56 (s, 1H), 8.82 (t, J = 5.9 Hz, 1H), 8.57 (d, J = 1.5 Hz, 1H), 8.07 (d, J = 8.0 Hz, 1H), 8.07 (d, J = 8.0 Hz, 1H), 7.94 (dd, J = 7.9, 2.14 Hz, 1H), 7.54 (s, 1H), 7.35 (dd, J = 7.9, 2.14 Hz, 1H), 7.25 (d, J = 8.3 Hz, 1H), 7.17−7.15 (m, 2H), 7.11 (d, J = 8.3 Hz, 1H), 6.38 (s, 1H), 4.39−4.39 (m, 1H), 3.37−3.33 (m, 2H), 3.98−3.96 (m, 1H), 3.76 (s, 2H), 2.96−2.94 (m, 1H), 2.54−2.50 (m, 1H), 2.39 (s, 3H), 2.32 (s, 3H), 2.21 (s, 3H), 1.73−1.70 (m, 2H), 1.55−1.45 (m, 3H), 0.97−0.91 (m, 2H); 13C NMR (150 MHz, DMSO-d_6) δ 148.9, 148.6, 148.0, 139.6, 138.3, 138.2, 137.6, 135.4, 134.6, 131.8, 131.7, 130.1, 131.7, 129.9, 127.3, 125.1, 121.8, 121.3, 119.9, 115.7, 54.0, 46.0, 41.9,

36.7, 36.2, 33.3, 32.6, 33.3, 32.6, 31.9, 21.1, 20.4, 18.9; HRMS (ESI) m/z calcd for $C_{33}H_{36}N_4O_3$ [M + H]+ 537.2860, found 537.2858.

N-(2-(1-(2-(4-methyl-2-oxo-1,2-dihydroquinolin-6-yl)acetyl)piperidin-4-yl)ethyl) picolinamide (12b): CDD-767

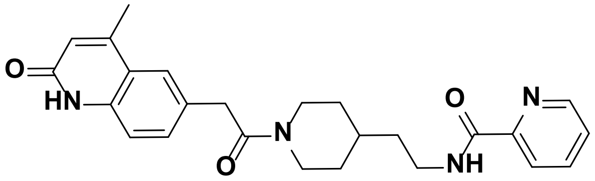

White solid; yield: 80%; ^{1}H NMR (600 MHz, DMSO-d_6) δ 11.55 (s, 1H), 8.77 (t, *J* = 5.9 Hz, 1H), 8.62 (d, *J* = 2.1 Hz, 1H), 8.05−8.02 (m, 1H), 7.99−7.96 (m, 1H), 7.59−7.57 (m, 1H), 7.53 (s, 1H), 7.34 (dd, *J* = 8.4, 1.8 Hz, 1H), 7.24 (d, *J* = 8.3 Hz, 1H), 6.38 (s, 1H), 4.37−4.35 (m, 1H), 3.97−3.95 (m, 1H), 3.75 (s, 2H), 3.34−3.31 (m, 2H), 2.97−2.93 (m, 1H), 2.54−2.50 (m, 1H), 2.38 (s, 3H), 1.70−1.69 (m, 2H), 1.53−1.43 (m, 3H), 0.97−0.89 (m, 2H); 13C NMR (150 MHz, DMSO-d_6) δ 168.5, 163.7, 161.5, 150.1, 148.3, 147.6, 137.7, 137.2, 131.4, 129.4, 126.3, 124.7, 121.8, 120.9, 119.4, 115.3, 45.5, 41.4, 39.0, 36.2, 35.7, 32.9, 32.1, 31.4, 18.4; HRMS (ESI) m/z calcd for $C_{25}H_{28}N_4O_3$ [M + H]+ 433.2234, found 433.2225.

N-(2-(1-(2-(4-methyl-2-oxo-1,2-dihydroquinolin-6-yl)acetyl)piperidin-4-yl)ethyl) benzamide (12c): CDD-779

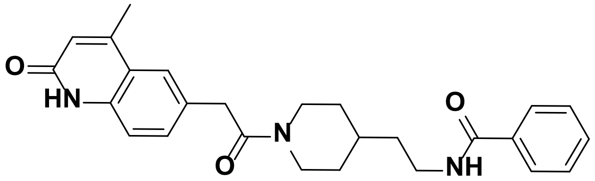

White solid; yield: 80%; ^{1}H NMR (600 MHz, DMSO-d_6) δ 11.55 (s, 1H), 8.41 (t, *J* = 5.4 Hz, 1H), 7.82 (d, *J* = 4.2 Hz, 1H), 7.54 (s, 1H), 7.52−7.49 (m, 1H), 7.47−7.43 (m, 2H), 7.35 (dd, *J* = 8.4, 1.5 Hz, 1H), 7.24 (d, *J* = 8.3 Hz, 1H), 6.38 (s, 1H), 4.39−4.37 (m, 1H), 3.98−3.96 (m, 1H), 3.76 (s, 2H), 3.30−3.27 (m, 2H), 2.98−2.94 (m, 1H), 2.55−2.50 (m, 1H), 2.39 (s, 3H), 1.70 (t, *J* = 11.4 Hz, 2H), 1.56−1.51 (m, 1H), 1.45−1.42 (m, 2H), 0.98−0.90 (m, 2H); 13C NMR (150 MHz, DMSO-d_6) δ 168.5, 166.0, 161.5, 150.5, 147.5, 139.6, 137.2, 134.6, 131.3, 130.9, 128.5, 127.0, 124.7, 120.9, 120.3, 119.4, 115.3, 45.5, 41.4, 39.0, 36.6, 35.6, 32.9, 32.2, 31.4, 18.4; HRMS (ESI) m/z calcd for $C_{26}H_{29}N_3O_3$ [M + H]+ 432.2282, found 432.2275.

N-(2-(1-(2-(4-methyl-2-oxo-1,2-dihydroquinolin-6-yl)acetyl)piperidin-4-yl)ethyl) quinoline-2-carboxamide (12d): CDD-855

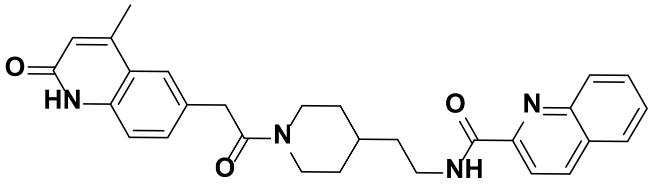

White solid; yield: 80%; ^{1}H NMR (600 MHz, DMSO-d_6) δ 11.58 (s, 1H), 8.92 (t, *J* = 6.0 Hz, 1H), 8.52 (d, *J* = 8.4 Hz, 1H), 8.15 (d, *J* = 8.4 Hz, 1H), 8.12 (d, *J* = 8.4 Hz, 1H), 8.04 (d, *J* = 8.0 Hz, 1H), 7.85−7.82 (m, 1H), 7.70−7.67 (m, 1H), 7.52 (s, 1H), 7.34 (dd, *J* = 8.9, 2.0 Hz, 1H), 7.25 (d, *J* = 8.4 Hz,

1H), 6.38 (s, 1H), 4.38−4.36 (m, 1H), 3.96−3.94 (m, 1H), 3.75 (s, 2H), 3.41−3.37 (m, 2H), 2.96−2.92 (m, 1H), 2.53−2.49 (m, 1H), 2.37 (s, 3H), 1.71−1.69 (m, 1H), 1.54−1.47 (m, 3H), 0.98−0.91 (m, 2H); 13C NMR (150 MHz, DMSO-d_6) δ 168.9, 162.9, 161.5, 148.4, 148.0, 147.5, 139.2, 137.8, 137.7, 137.2, 134.9, 134.1, 131.2, 129.6, 129.4, 126.8, 124.6, 121.4, 119.4, 115.3, 45.5, 41.8, 41.4, 32.4, 31.8, 31.2, 20.6, 19.8, 18.4; HRMS (ESI) m/z calcd for $C_{29}H_{30}N_4O_3$ [M + H]+ 483.2391, found 483.2382.

N-(2-(1-(2-(4-methyl-2-oxo-1,2-dihydroquinolin-6-yl)acetyl)piperidin-4-yl)ethyl) isoquinoline-3-carboxamide (12e): CDD-854

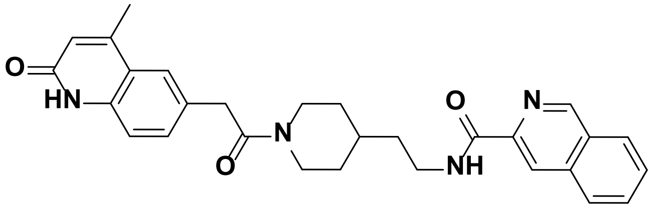

White solid; yield: 81%; ^{1}H NMR (600 MHz, DMSO-d_6) δ 11.54 (s, 1H), 9.37 (s, 1H), 8.91 (t, J = 5.9 Hz, 1H), 8.54 (s, 1H), 8.24 (d, J = 8.0 Hz, 1H), 8.15 (d, J = 8.1 Hz, 1H), 7.87 (t, J = 4.0 Hz, 1H), 7.80 (t, J = 4.0 Hz, 1H), 7.54 (s, 1H), 7.35 (dd, J = 8.4, 1.5 Hz, 1H), 7.24 (d, J = 8.3 Hz, 1H), 6.38 (s, 1H), 4.38−4.36 (m, 1H), 3.99−3.96 (m, 1H), 3.76 (s, 2H), 3.41−3.37 (m, 2H), 2.99−2.95 (m, 1H), 2.56−2.50 (m, 1H), 2.39 (s, 3H), 1.74 (s, 2H), 1.57−1.47 (m, 3H), 0.98−0.94 (m, 2H); 13C NMR (150 MHz, DMSO-d_6) δ 168.4, 163.9, 161.4, 151.4, 147.5, 143.9, 137.1, 135.3, 131.3, 131.2, 129.4, 129.1, 128.9, 129.1, 128.9, 127.9, 127.7, 124.6, 120.8, 119.5, 119.3, 115.2, 45.5, 41.8, 41.4, 35.8, 32.9, 32.1, 18.4, 18.0, 16.7; HRMS (ESI) m/z calcd for $C_{29}H_{30}N_4O_3$ [M + H]+ 483.2391, found 483.2387.

N-(2-(1-(2-(4-methyl-2-oxo-1,2-dihydroquinolin-6-yl)acetyl)piperidin-4-yl)ethyl) isoquinoline-1-carboxamide (12f): CDD-853

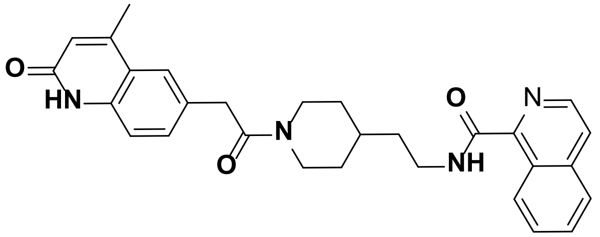

White solid; yield: 84%; ^{1}H NMR (600 MHz, DMSO-d_6) δ 11.57 (s, 1H), 8.91 (d, J = 8.5 Hz, 1H), 8.83 (t, J = 5.8 Hz, 1H), 8.52 (d, J = 5.5 Hz, 1H), 8.02 (d, J = 8.2 Hz, 1H), 7.98 (d, J = 5.5 Hz, 1H), 7.81−7.79 (m, 1H), 7.72−7.70 (m, 1H), 7.54 (s, 1H), 7.35 (dd, J = 8.4, 1.5 Hz, 1H), 7.25 (d, J = 8.3 Hz, 1H), 6.38 (s, 1H), 4.40−4.38 (m, 1H), 3.99−3.97 (m, 1H), 3.76 (s, 2H), 3.40−3.37 (m, 2H), 2.98−2.94 (m, 1H), 2.55−2.50 (m, 1H), 2.38 (s, 3H), 1.74−1.72 (m, 2H), 1.59−1.55 (m, 1H), 1.51−1.47 (m, 2H), 1.00−0.93 (m, 2H); 13C NMR (150 MHz, DMSO-d_6) δ 168.5, 166.0, 161.5, 151.4, 147.5, 140.7, 137.1, 136.5, 131.3, 130.5, 129.4, 128.2, 127.0, 126.5, 125.4, 124.6, 123.1, 120.8, 119.4, 115.3, 45.5, 41.8, 41.4, 36.3, 35.6, 32.9, 32.1, 18.4, 18.0, 16.6; HRMS (ESI) m/z calcd for $C_{23}H_{30}N_4O_3$ [M + H]+ 483.2391, found 483.2389.

N-(2-(4-(2-(4-methyl-2-oxo-1,2-dihydroquinolin-6-yl)acetyl)piperazin-1-yl)ethyl) picolinamide (12g): CDD-778

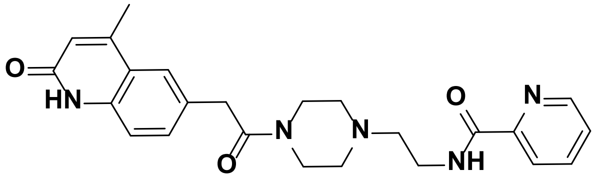

White solid; yield: 81%; ^{1}H NMR (600 MHz, CD_3OD) δ 8.63 (d, *J* = 4.4 Hz, 1H), 8.11 (d, *J* = 7.8 Hz, 1H), 7.99−7.96 (m, 1H), 7.68 (s, 1H), 7.58−7.96 (m, 1H), 7.46 (dd, *J* = 8.4, 1.6 Hz, 1H), 7.33 (d, *J* = 8.4 Hz, 1H), 6.52 (s, 1H), 3.95 (s, 2H), 8.63 (d, *J* = 4.4 Hz, 1H), 3.73−3.35 (m, 6H), 3.31−3.30 (m, 4H), 2.51 (s, 3H); 13C NMR (150 MHz, DMSO-d_6) δ 172.1, 168.1, 164.8, 151.5, 150.3, 149.8, 138.9, 138.3, 133.1, 130.7, 128.1, 126.4, 123.3, 121.9, 120.9, 117.4, 58.0, 53.09 (2x), 43.8, 39.9, 35.2, 19.08 (2x); HRMS (ESI) m/z calcd for $C_{24}H_{27}N_5O_4$ [M + H]+ 434.2187, found 434.2186.

2) ^{1}H and ^{13}C NMR spectra of most selective compounds, **6a** (**CDD-724**), **6c** (**CDD-787**), **6d** (**CDD-956**), **6f** (**CDD-981**), **6g** (**CDD-982**), **6h** (**CDD-986**), and **6j** (**CDD-1146**).

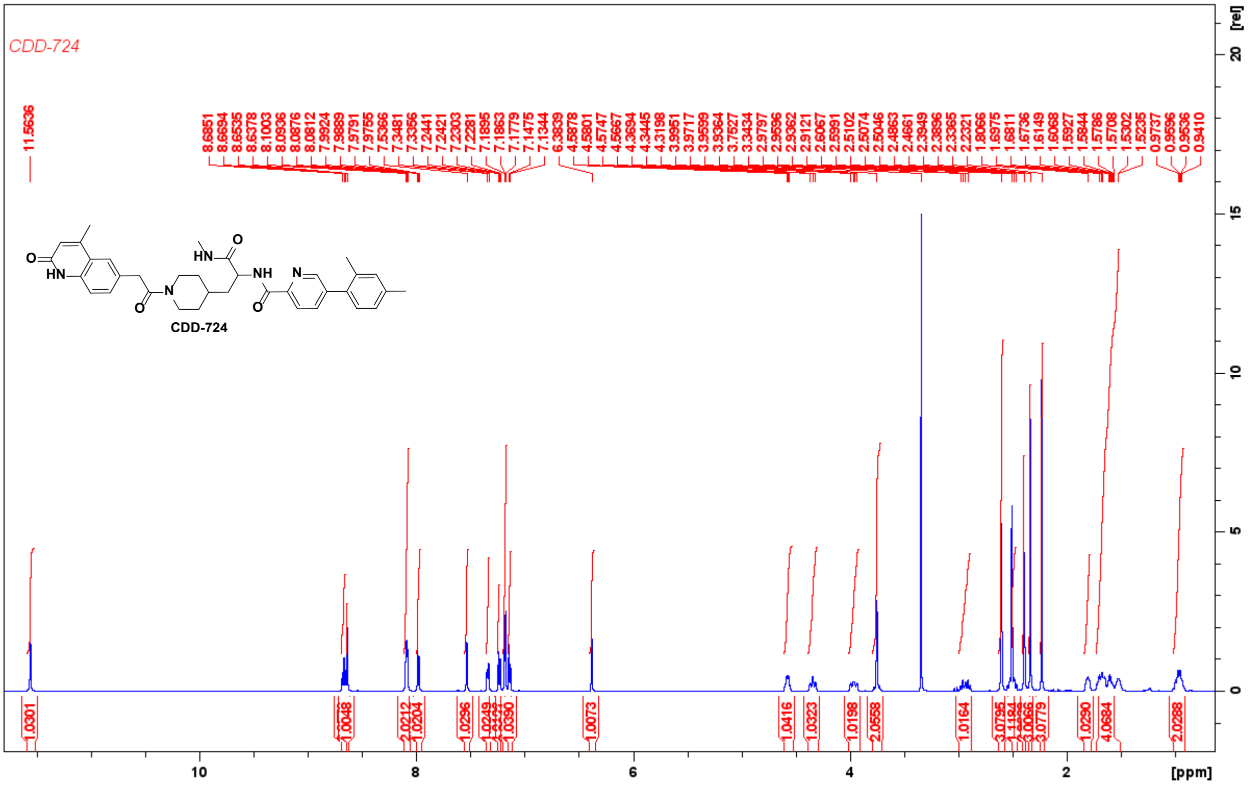

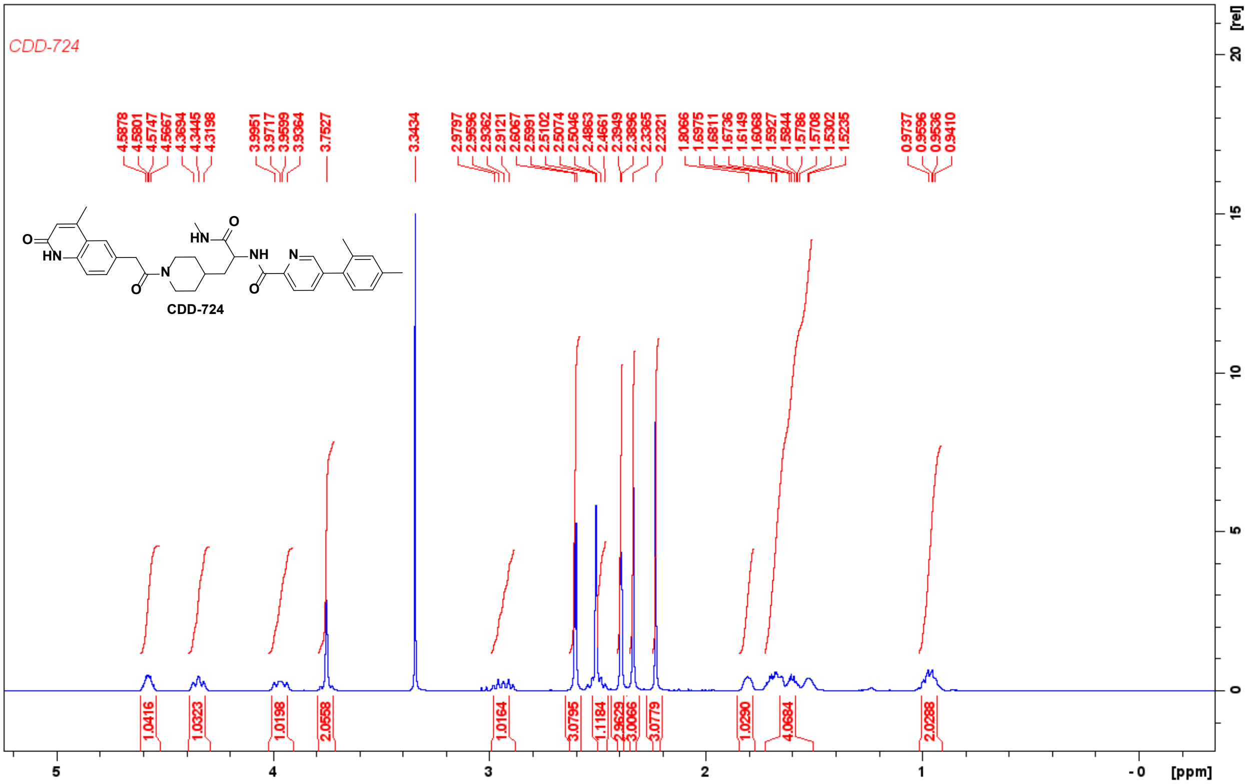

^{1}H NMR (DMSO-d_6, 600 MHz) and zoom-in (0–5 ppm) spectra of **6a** (**CDD-724)**

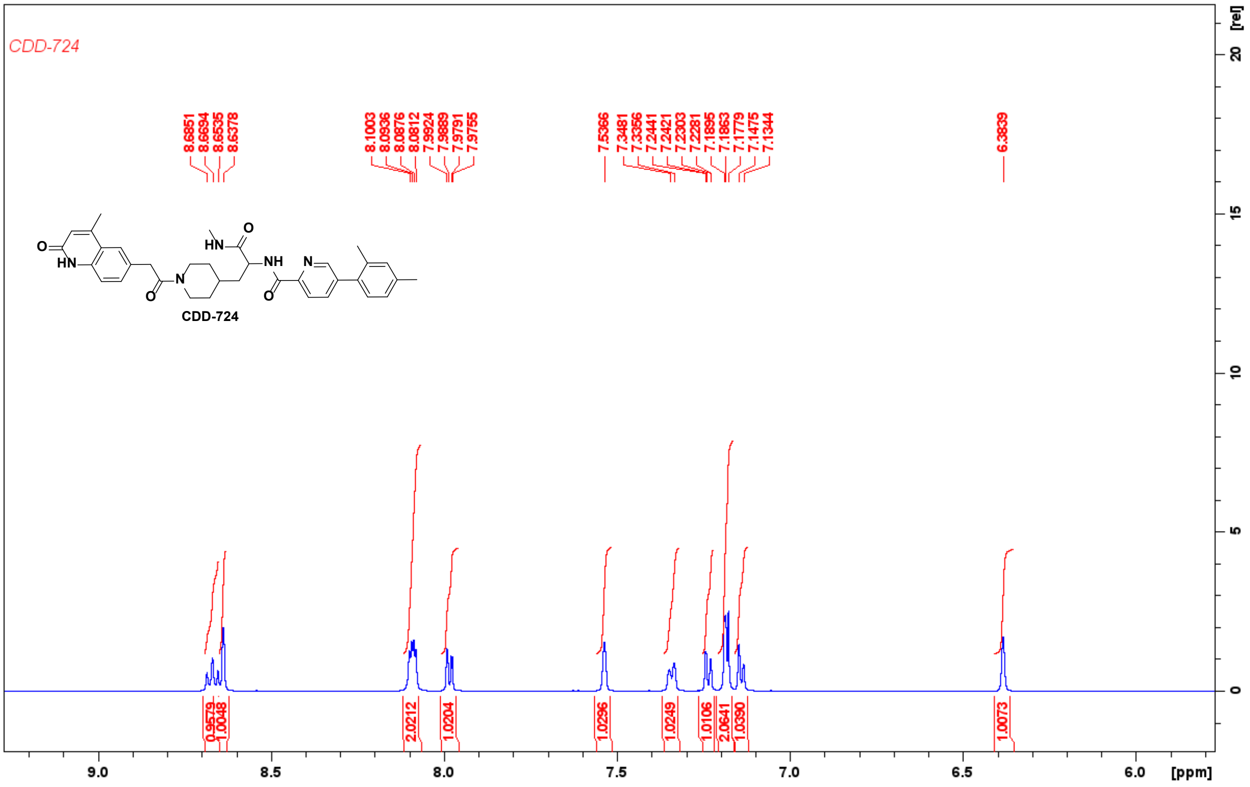

^{1}H NMR (DMSO-*d*₆, 600 MHz) and zoom-in (6–9 ppm) spectra of **6a** (**CDD-724**)

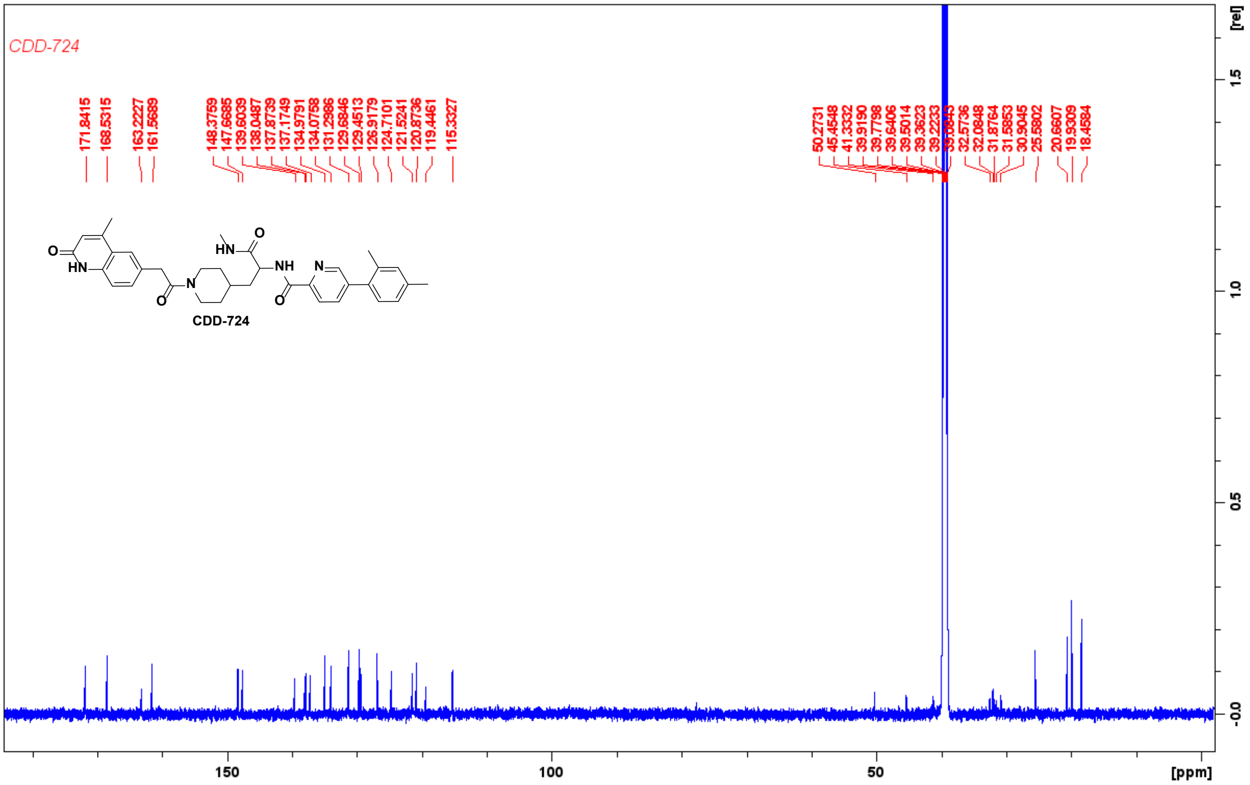

^{13}C NMR (DMSO-*d*₆, 150 MHz) spectrum of **6a** (**CDD-724)**

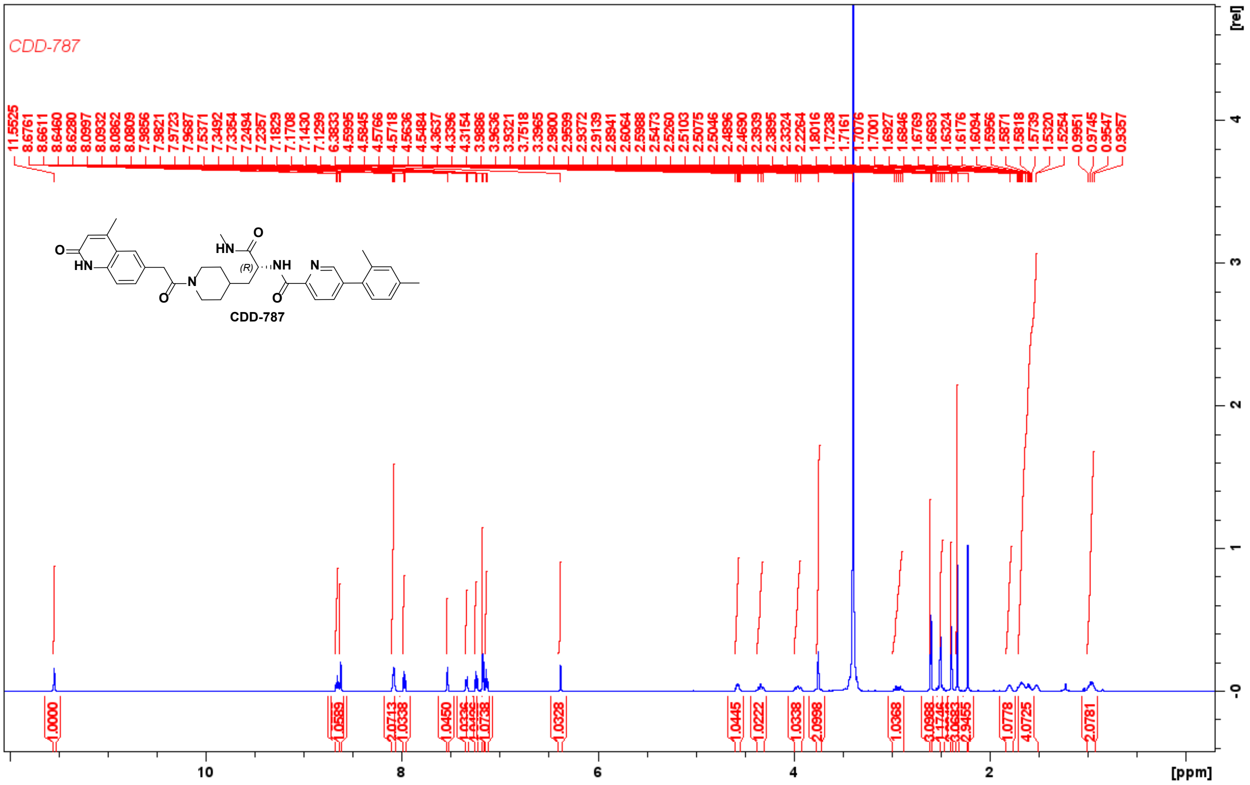

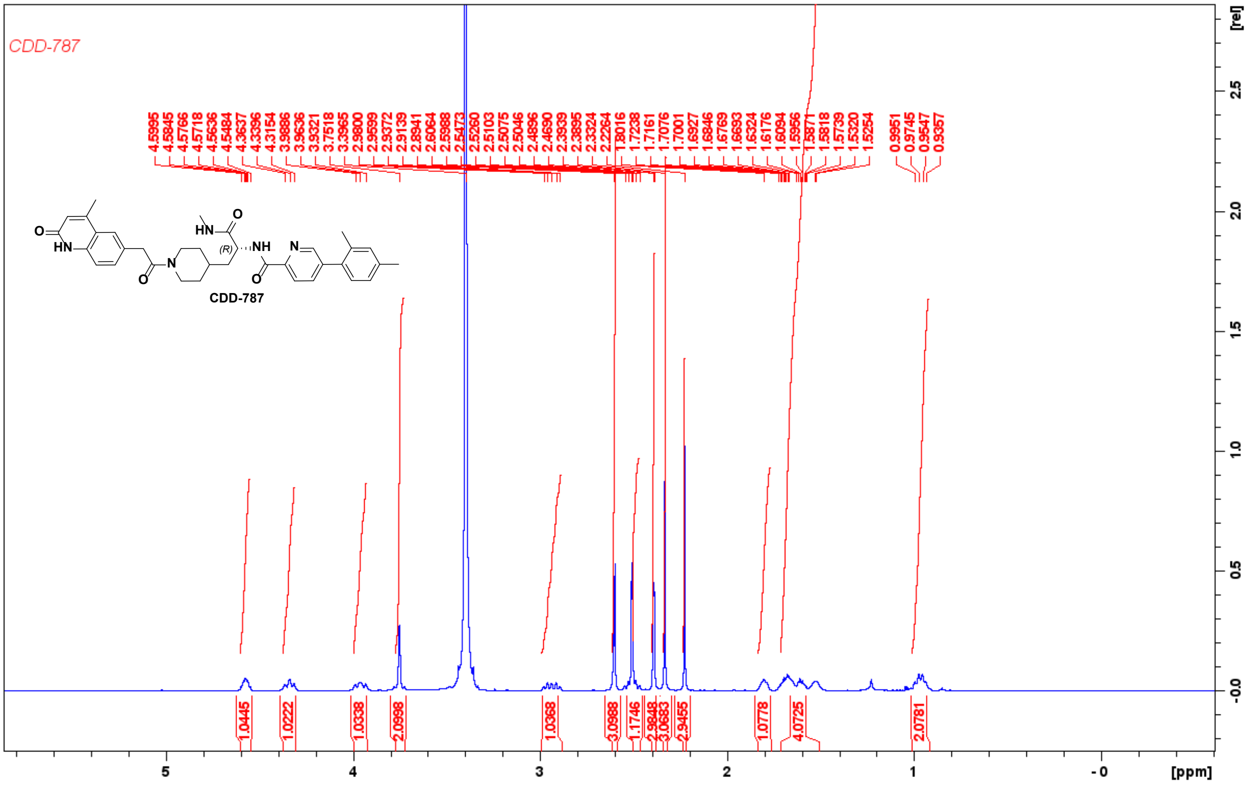

^{1}H NMR (DMSO-d_6, 600 MHz) and zoom-in (0–5 ppm) spectra of **6c** (**CDD-787)**

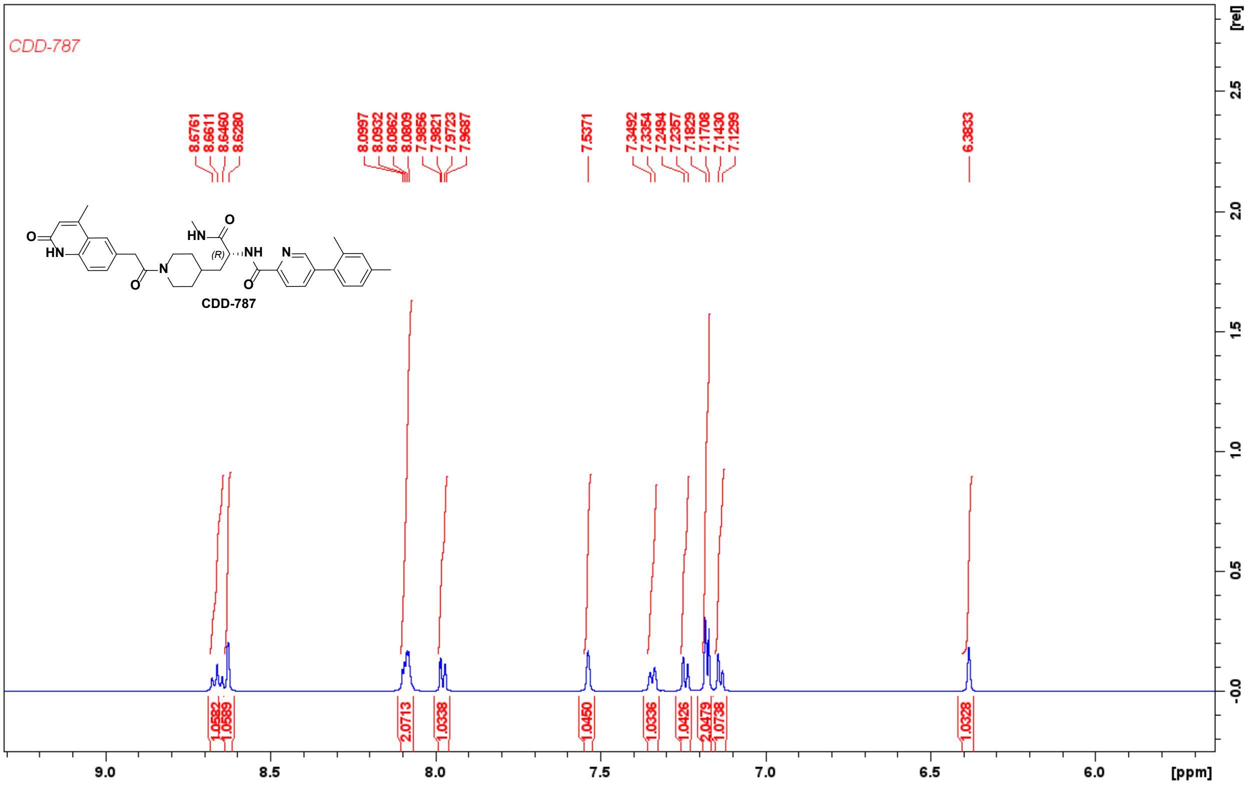

^{1}H NMR (DMSO-d_6, 600 MHz) and zoom-in (6–9 ppm) spectra of **6c** (**CDD-787**)

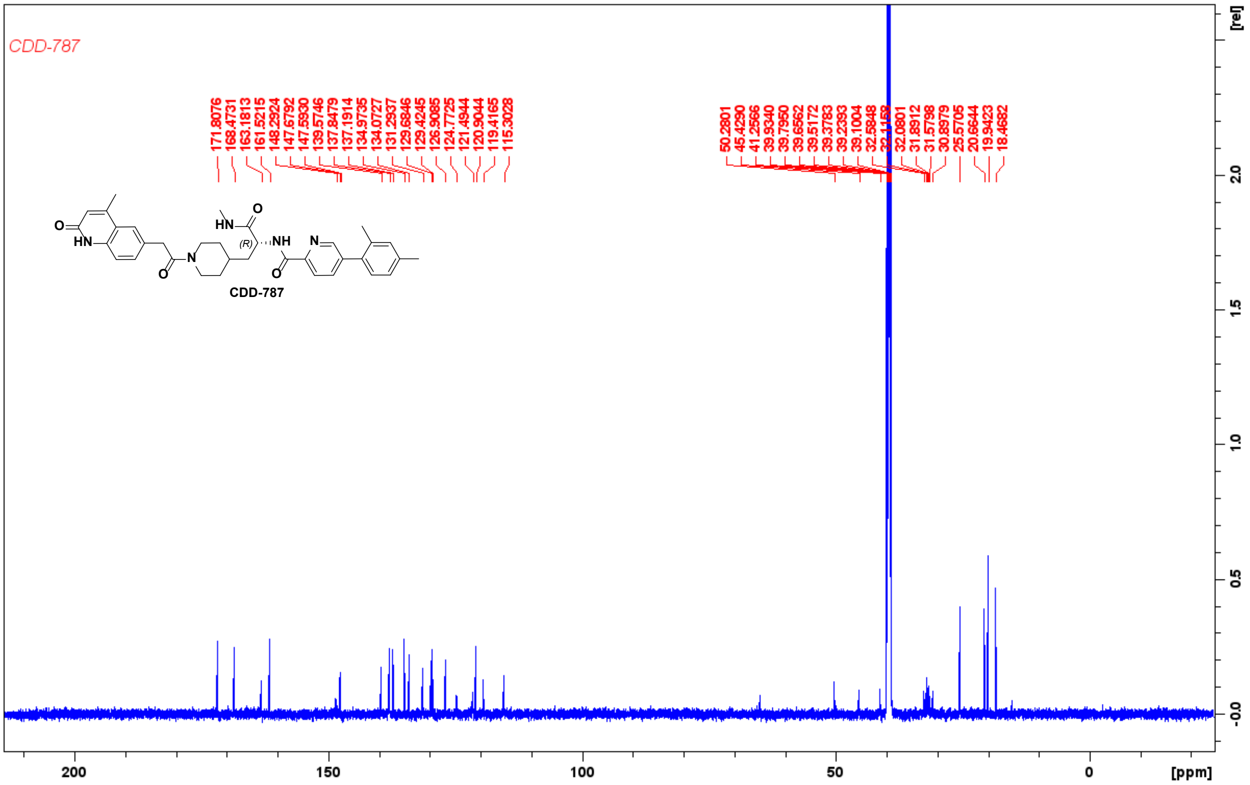

^{13}C NMR (DMSO-d_6, 150 MHz) spectrum of **6c** (**CDD-787**)

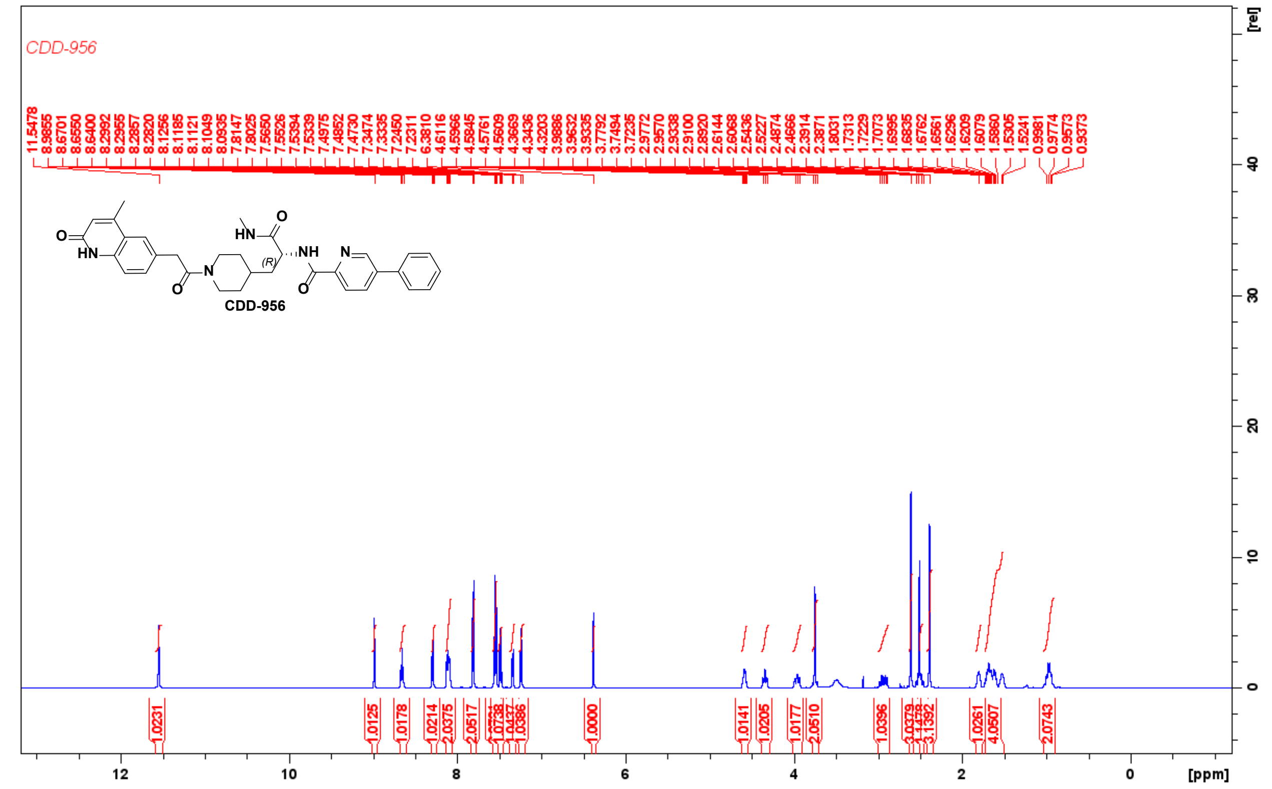

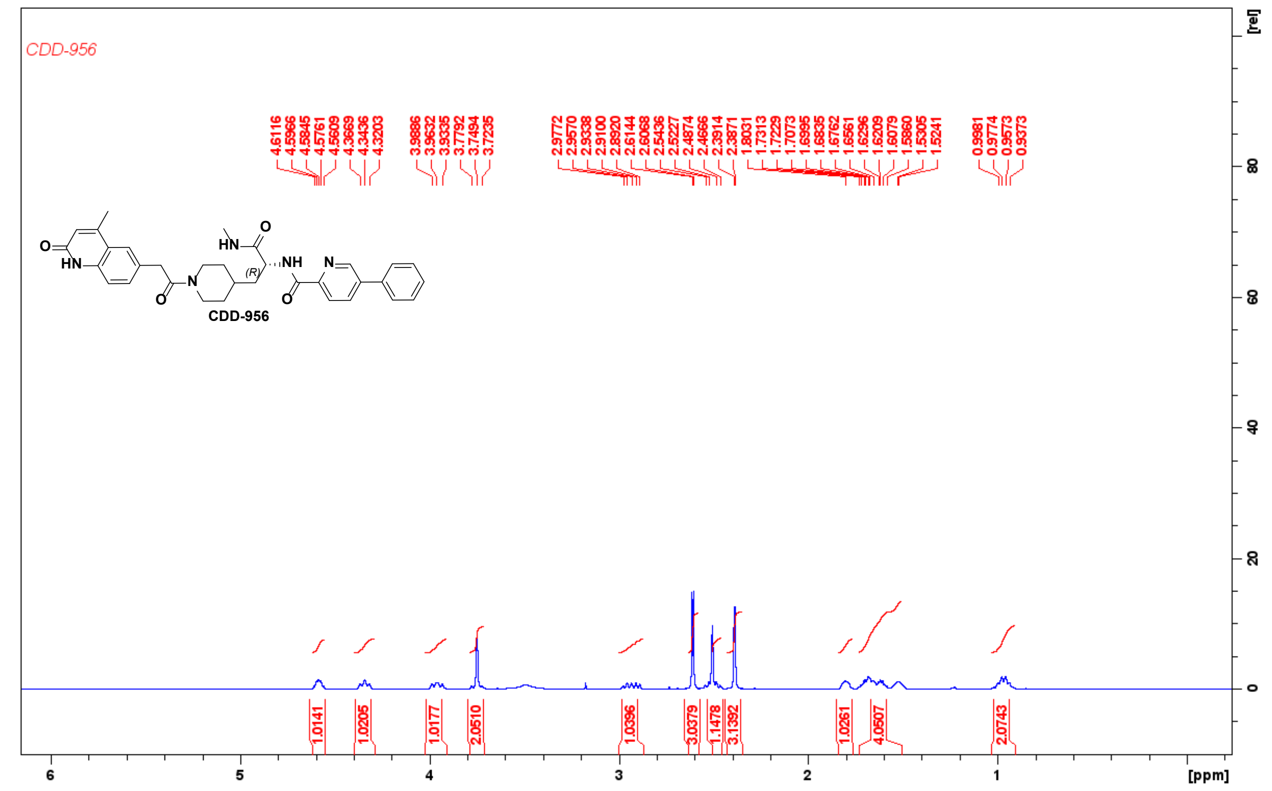

^{1}H NMR (DMSO-d_6, 600 MHz) and zoom-in (0–6 ppm) spectra of **6d (CDD-956)**

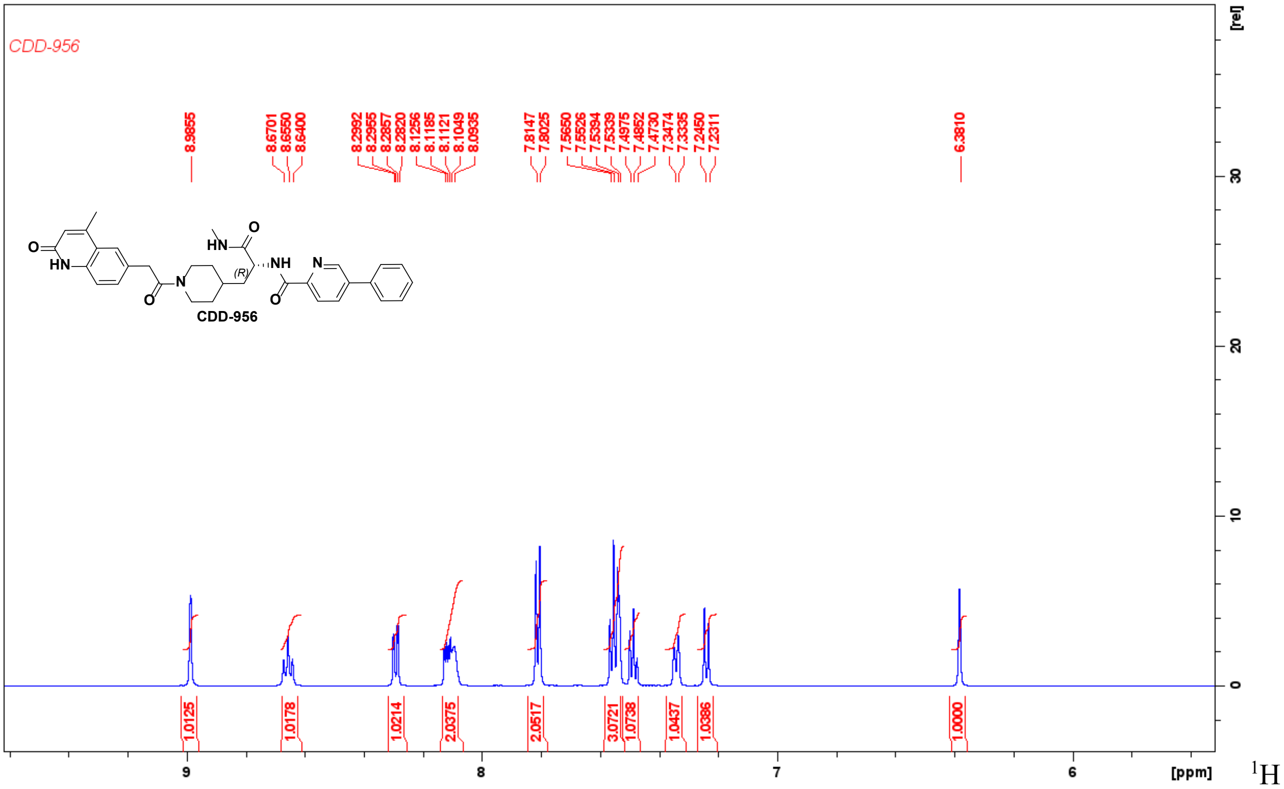

NMR (DMSO-d_6, 600 MHz) and zoom-in (6–9 ppm) spectra of **6d** (**CDD-956**)

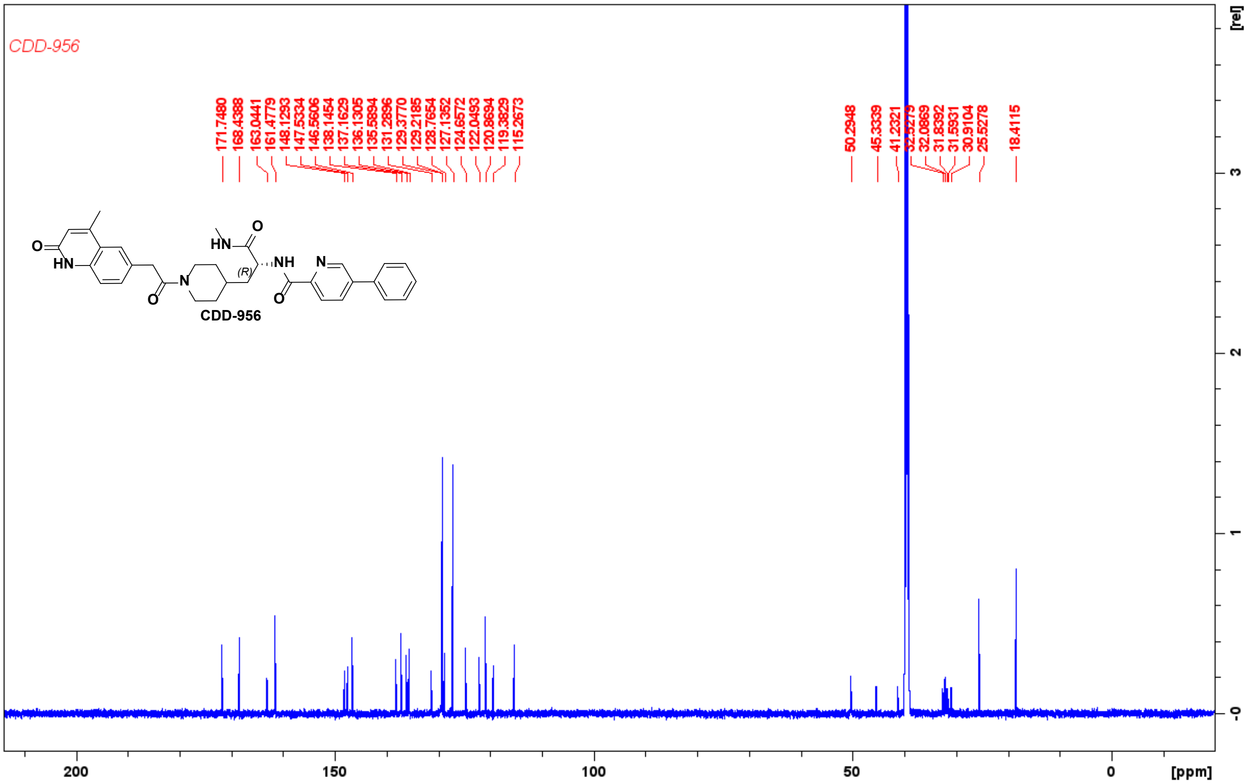

^{13}C NMR (DMSO-d_6, 150 MHz) spectrum of **6d** (**CDD-956**)

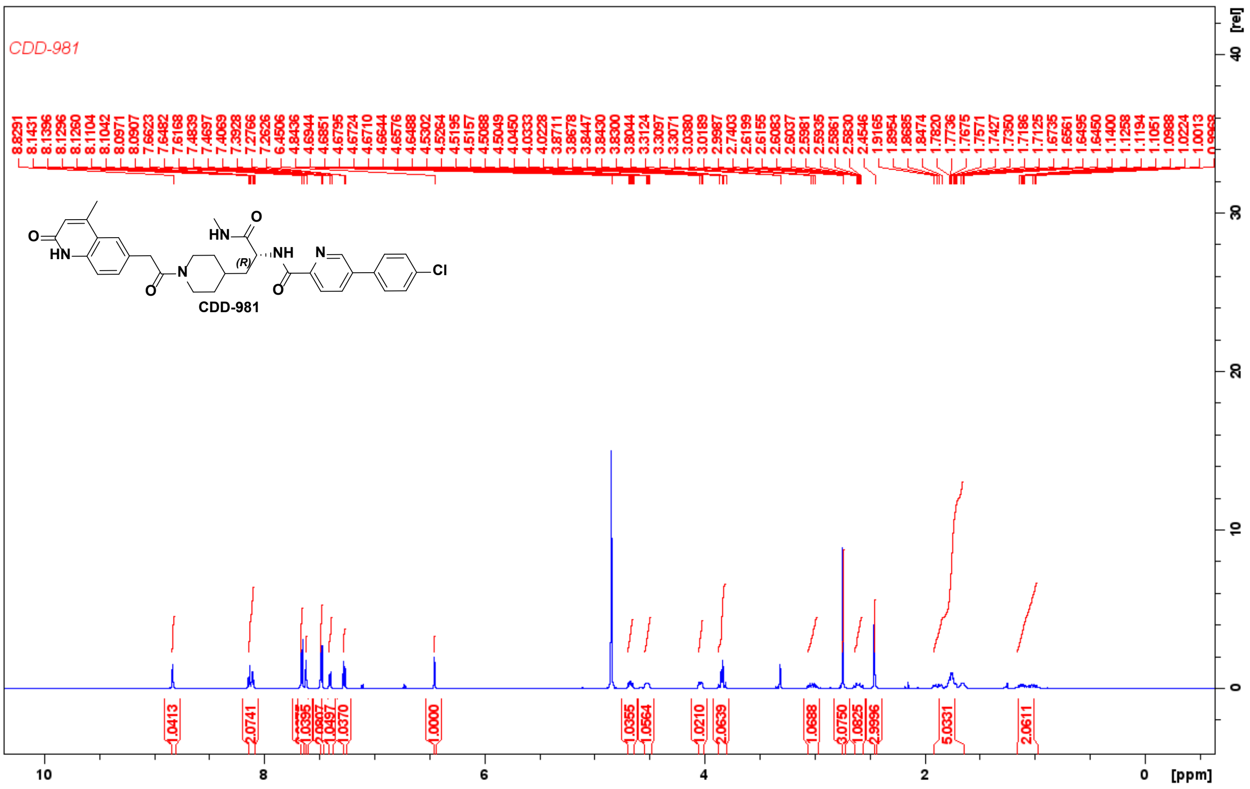

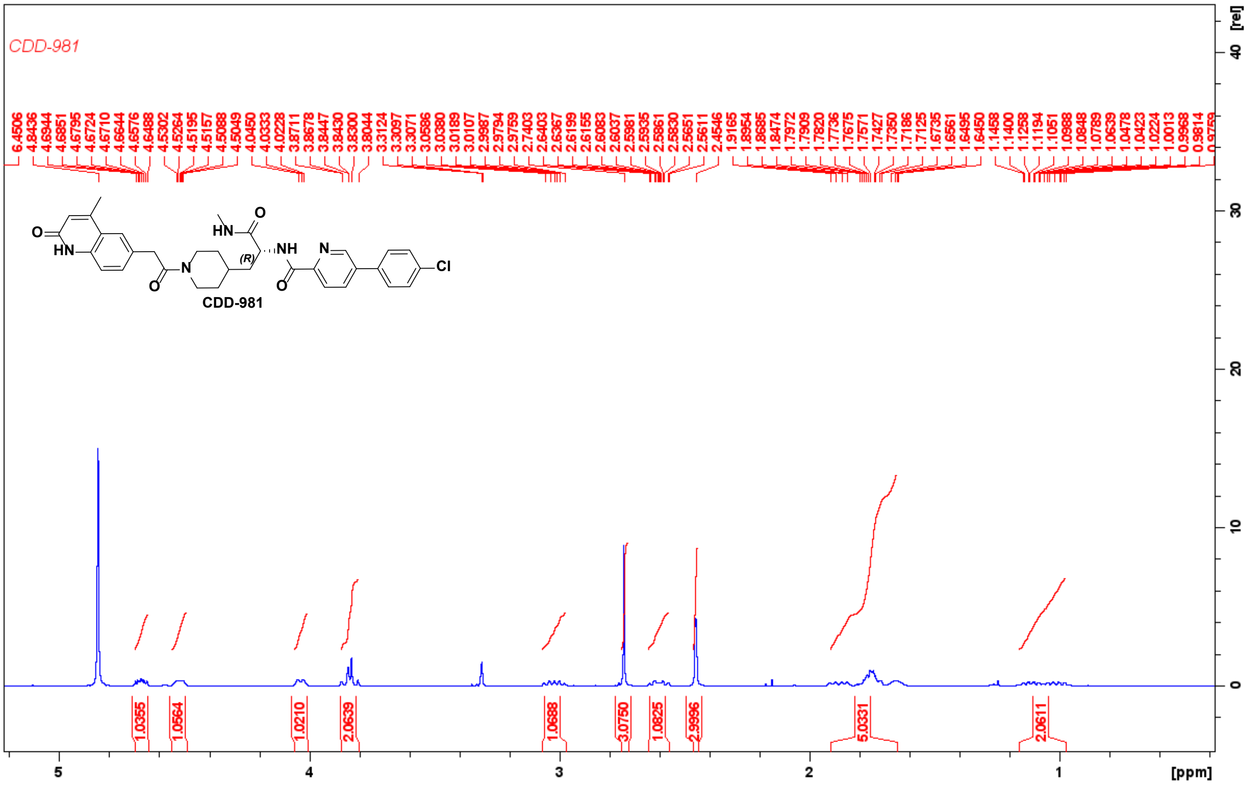

^{1}H NMR (CD_3OD, 600 MHz) and zoom-in (0–5 ppm) spectra of **6f** (**CDD-981)**

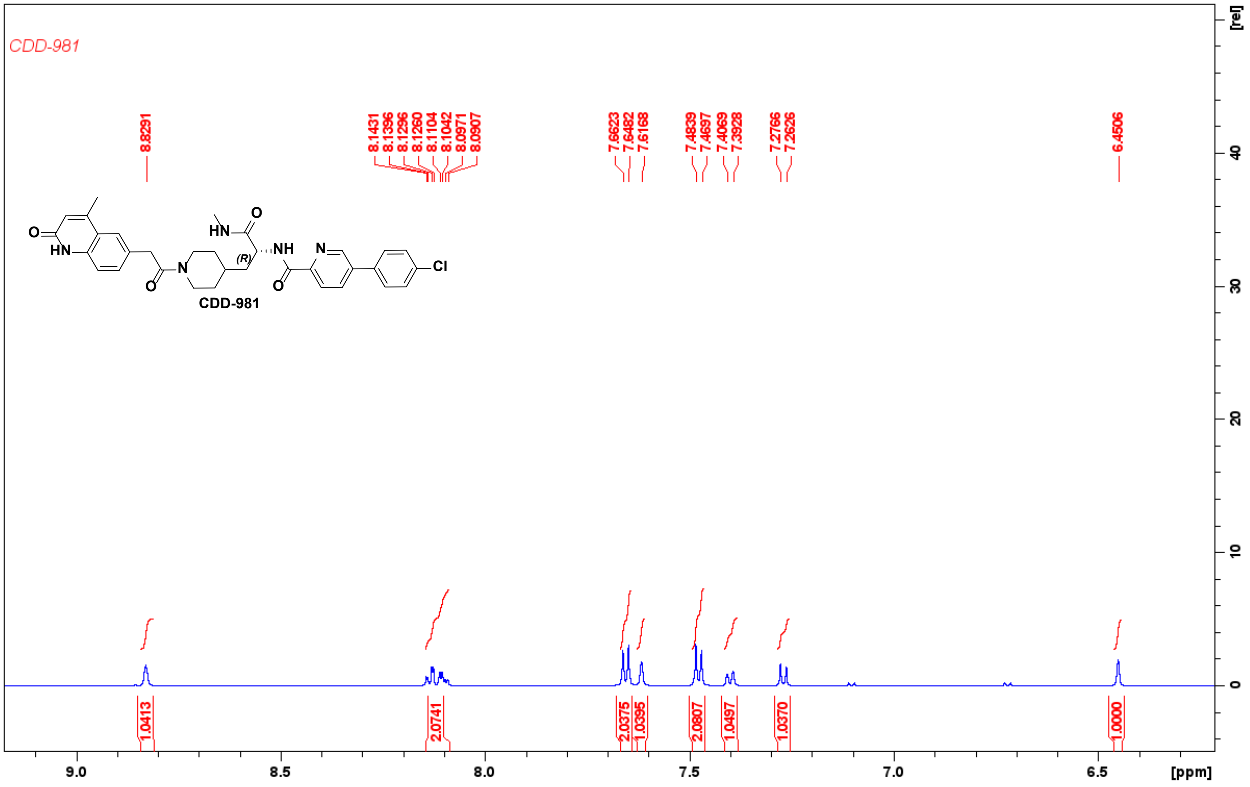

^{1}H NMR (CD_3OD, 600 MHz) and zoom-in (6–9 ppm) spectra of **6h** (**CDD-981**)

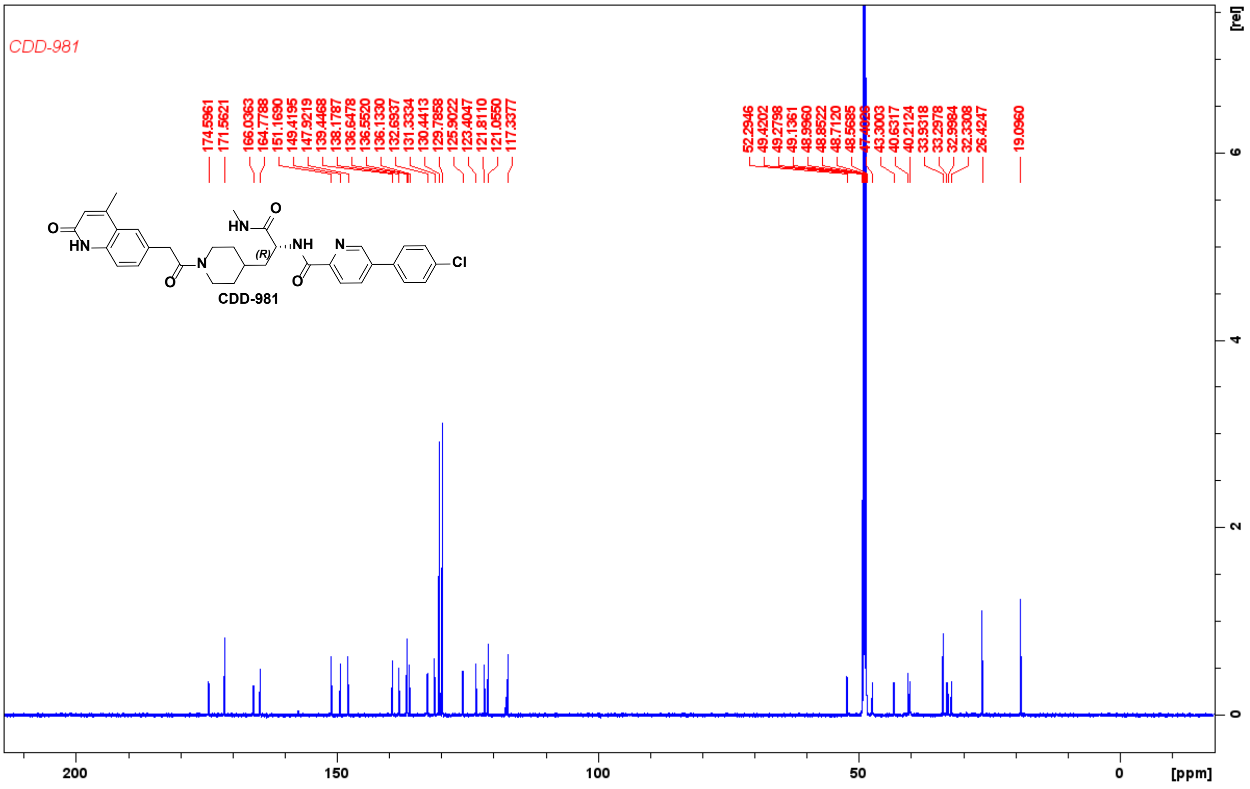

^{13}C NMR (CD_3OD, 150 MHz) spectrum of **6f** (**CDD-981**)

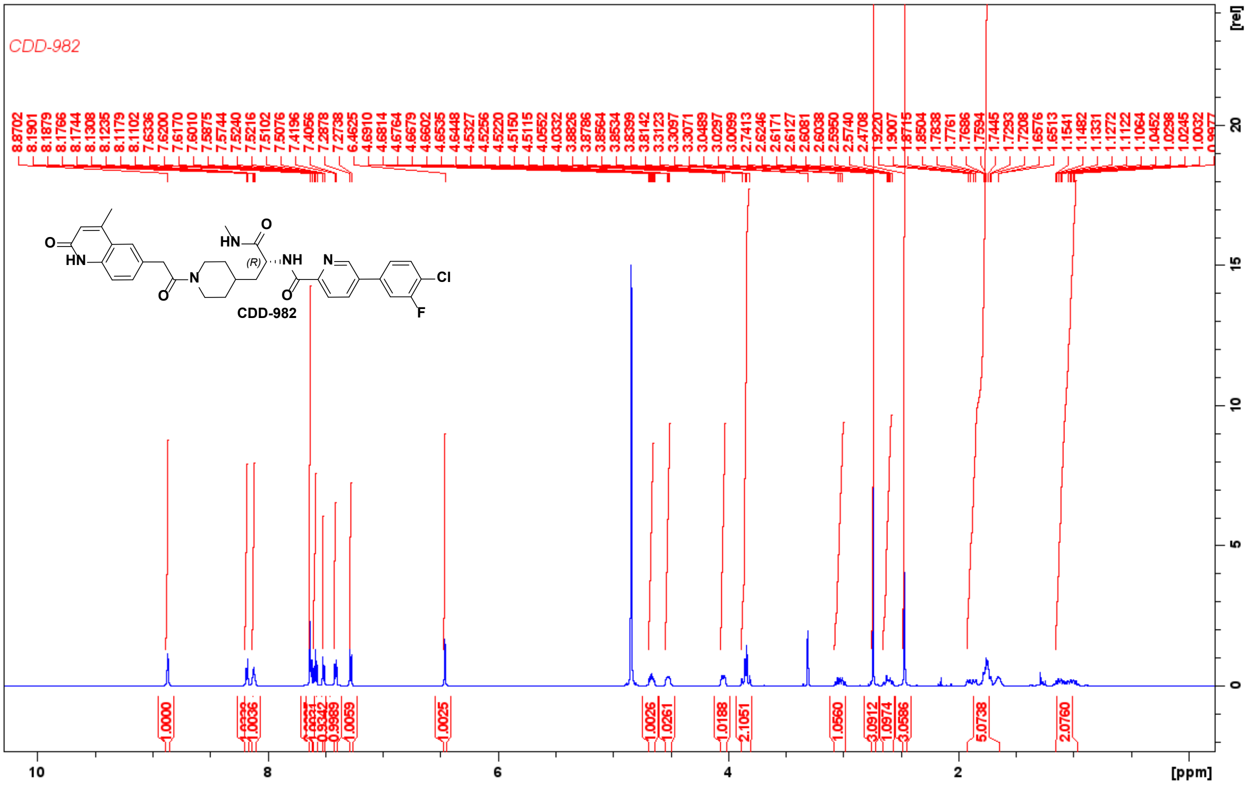

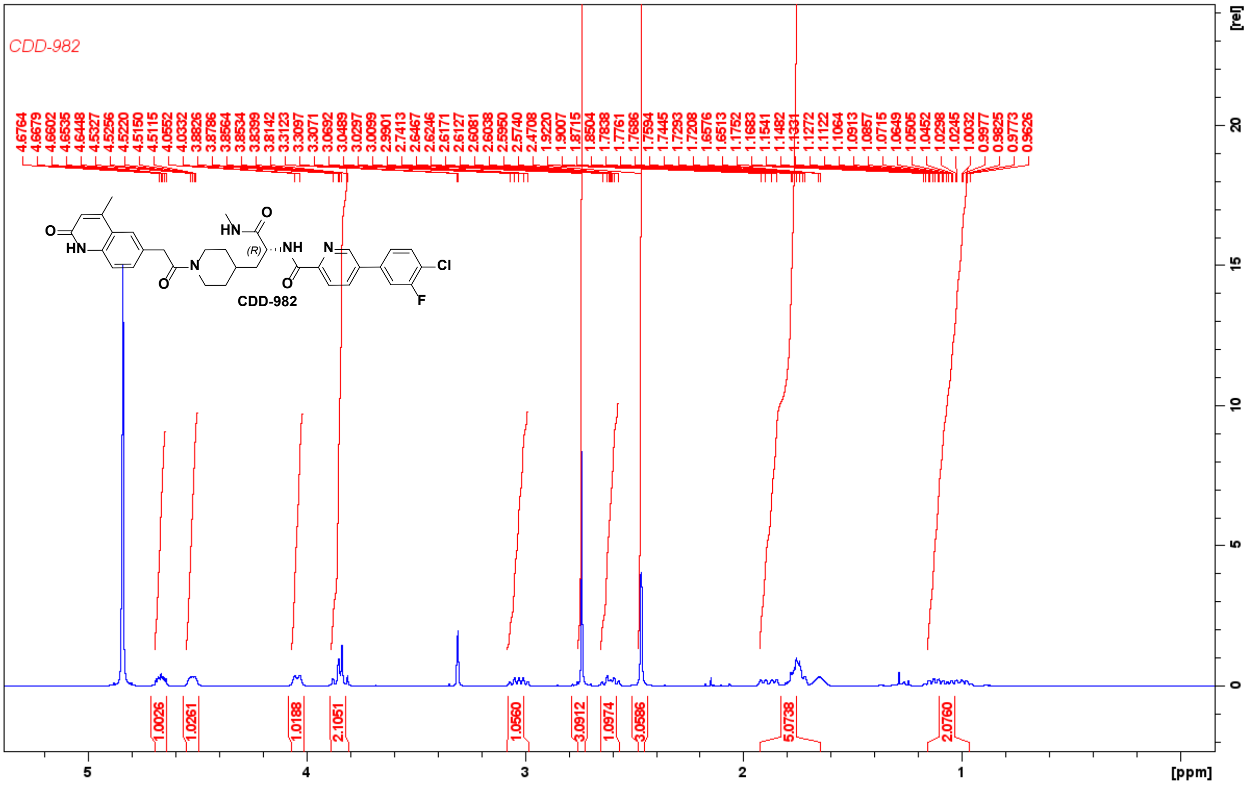

^{1}H NMR (CD_3OD, 600 MHz) and zoom-in (0–5 ppm) spectra of **6g** (**CDD-982)**

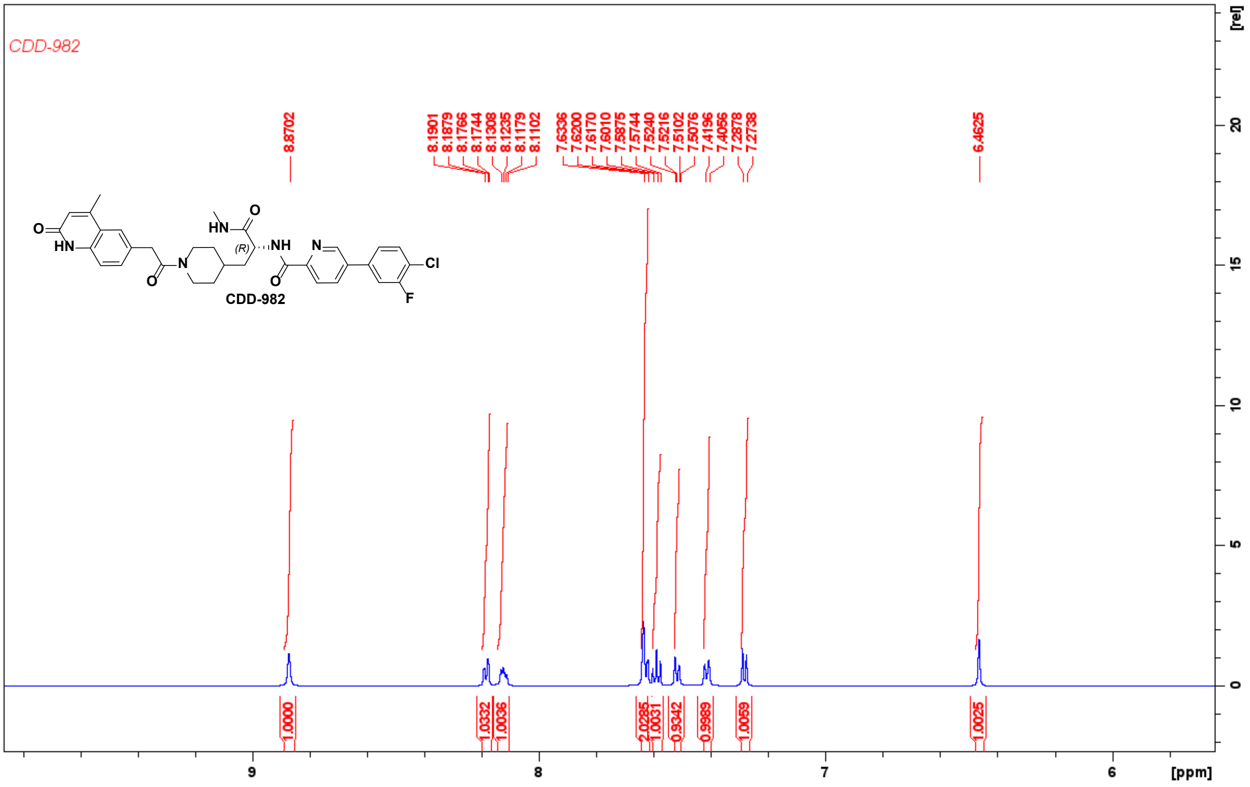

^{1}H NMR (CD_3OD, 600 MHz) and zoom-in (6–9 ppm) spectra of **6g** (**CDD-982**)

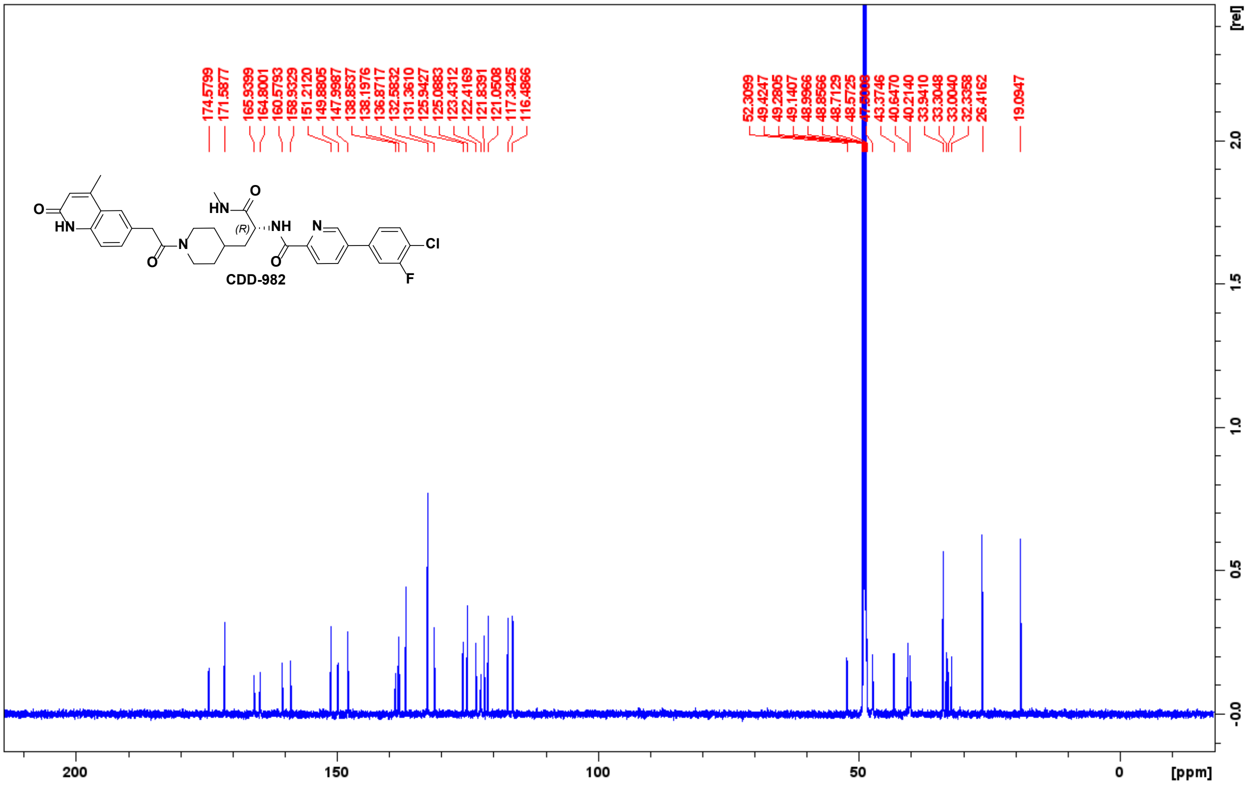

^{13}C NMR (CD_3OD, 150 MHz) spectrum of 6**g** (**CDD-982**)

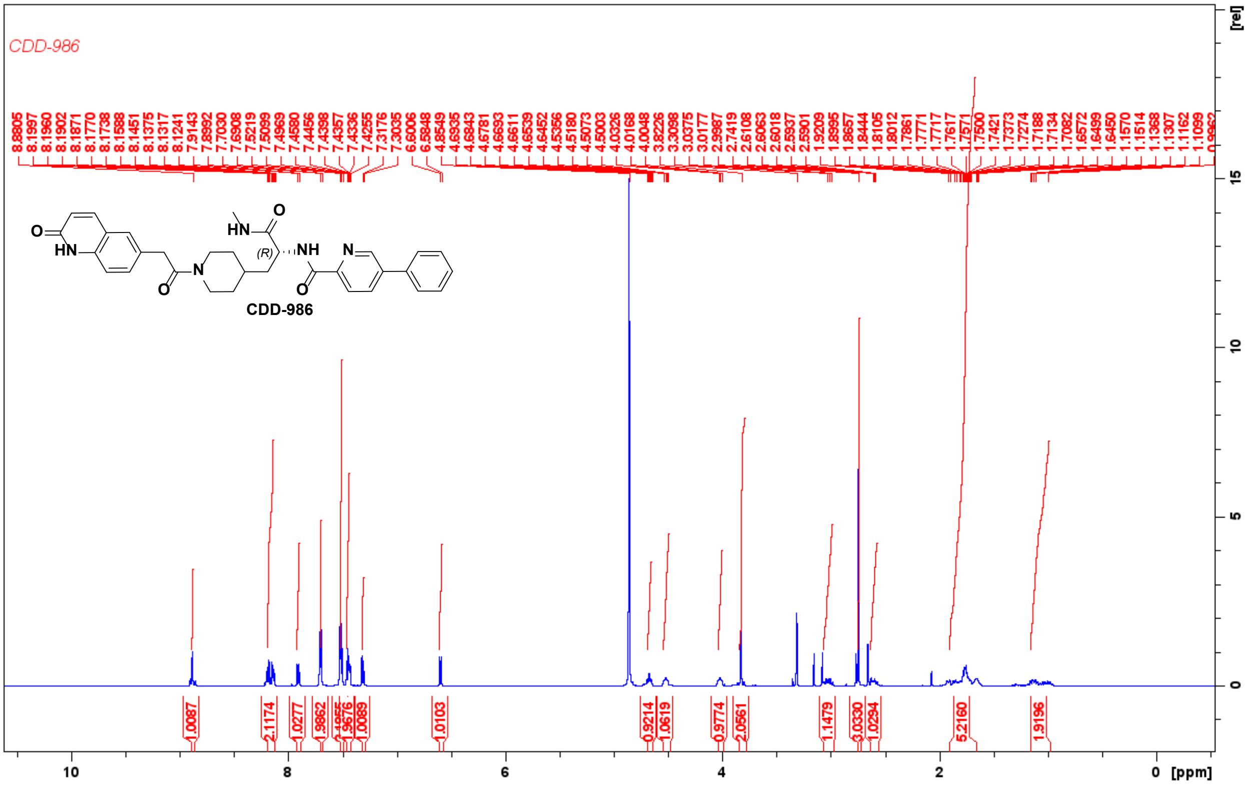

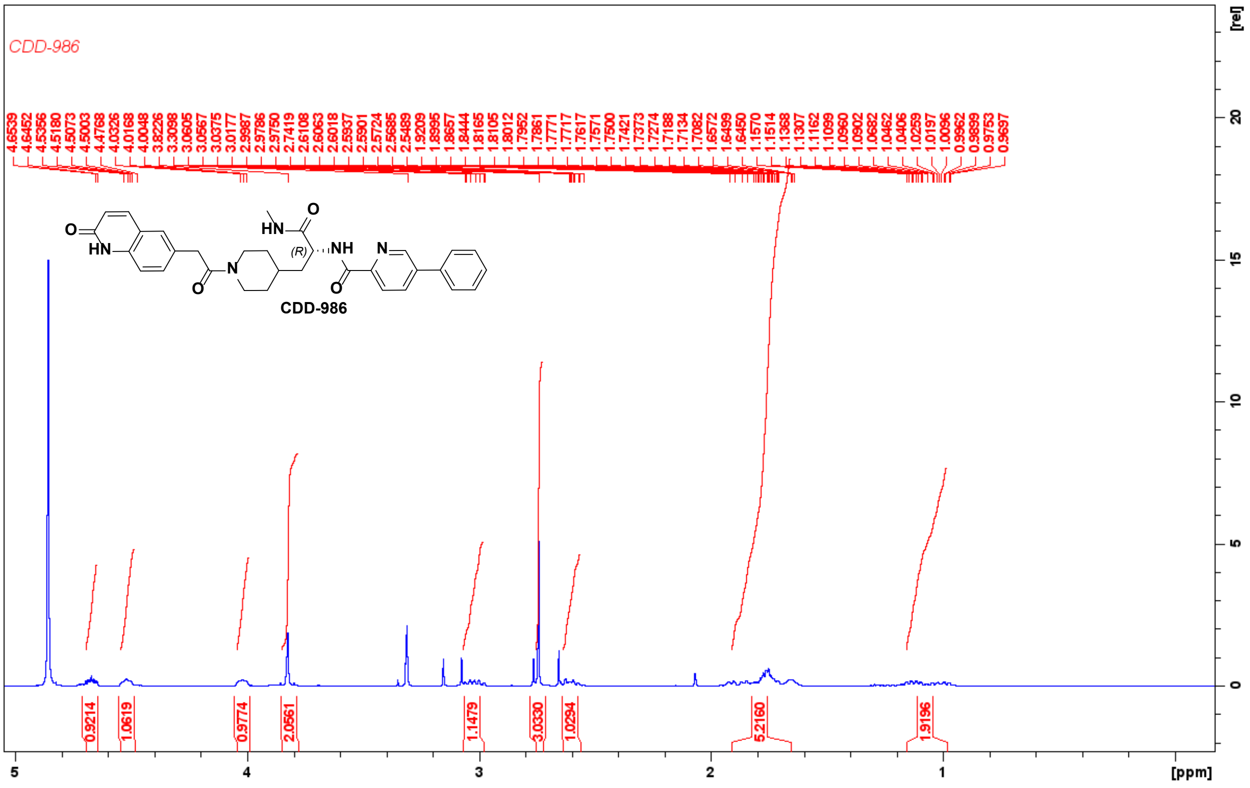

^{1}H NMR (CD_3OD, 600 MHz) and zoom-in (0–5 ppm) spectra of **6h** (**CDD-986)**

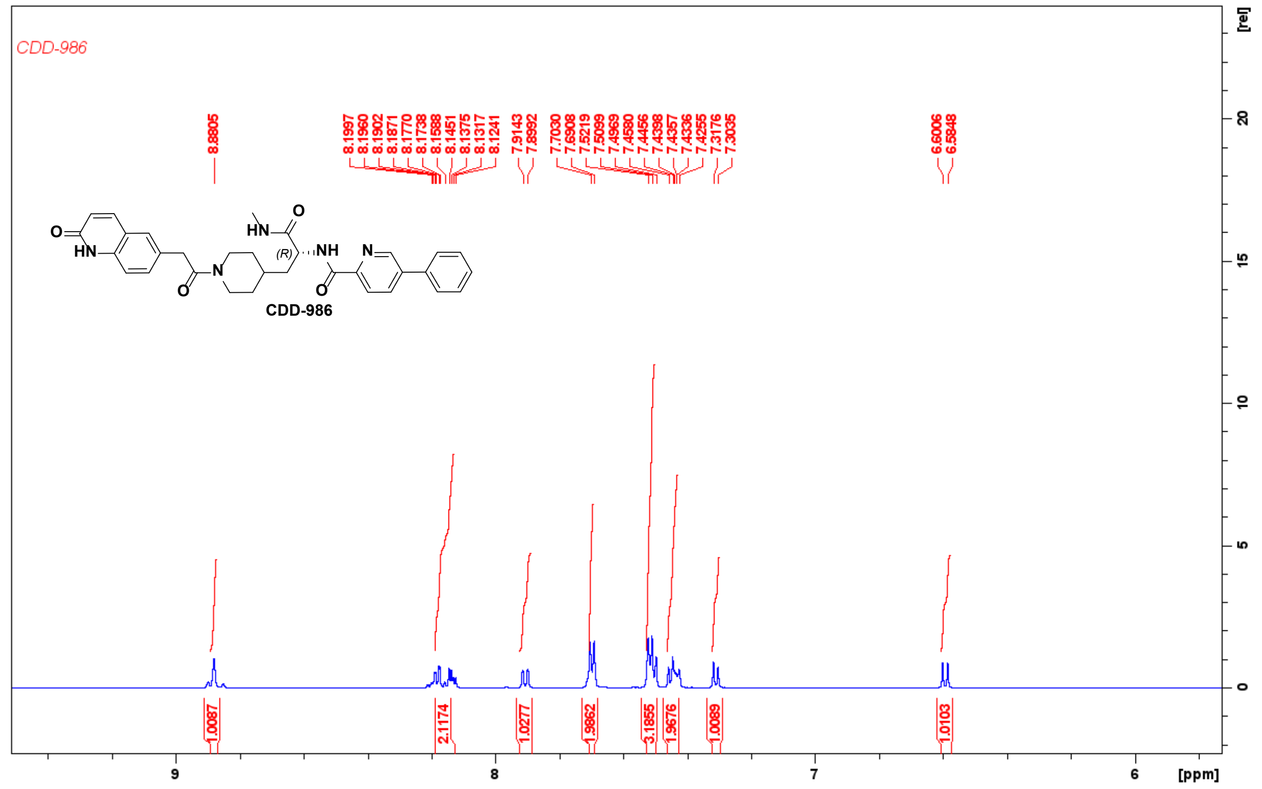

^{1}H NMR (CD3OD, 600 MHz) and zoom-in (6–9 ppm) spectra of **6h** (**CDD-986**)

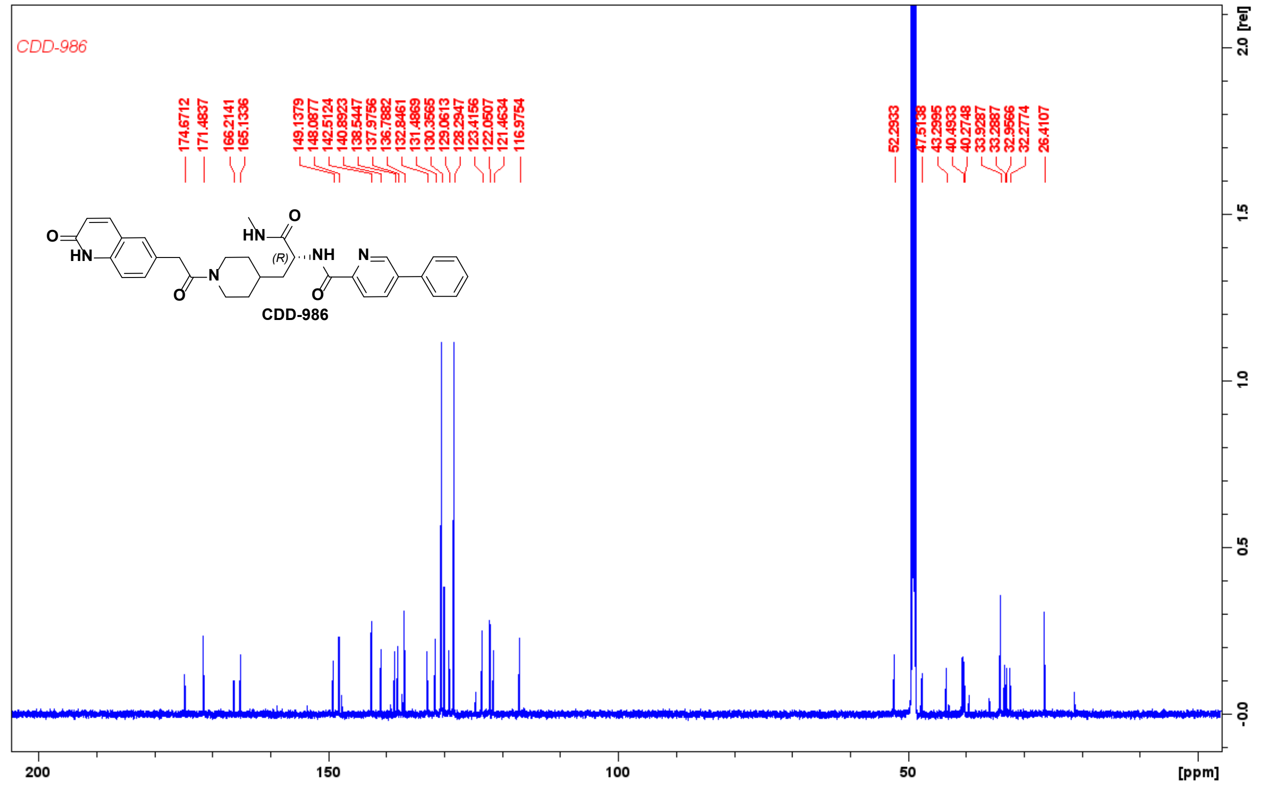

^{13}C NMR (CD3OD, 150 MHz) spectrum of **6h** (**CDD-986**)

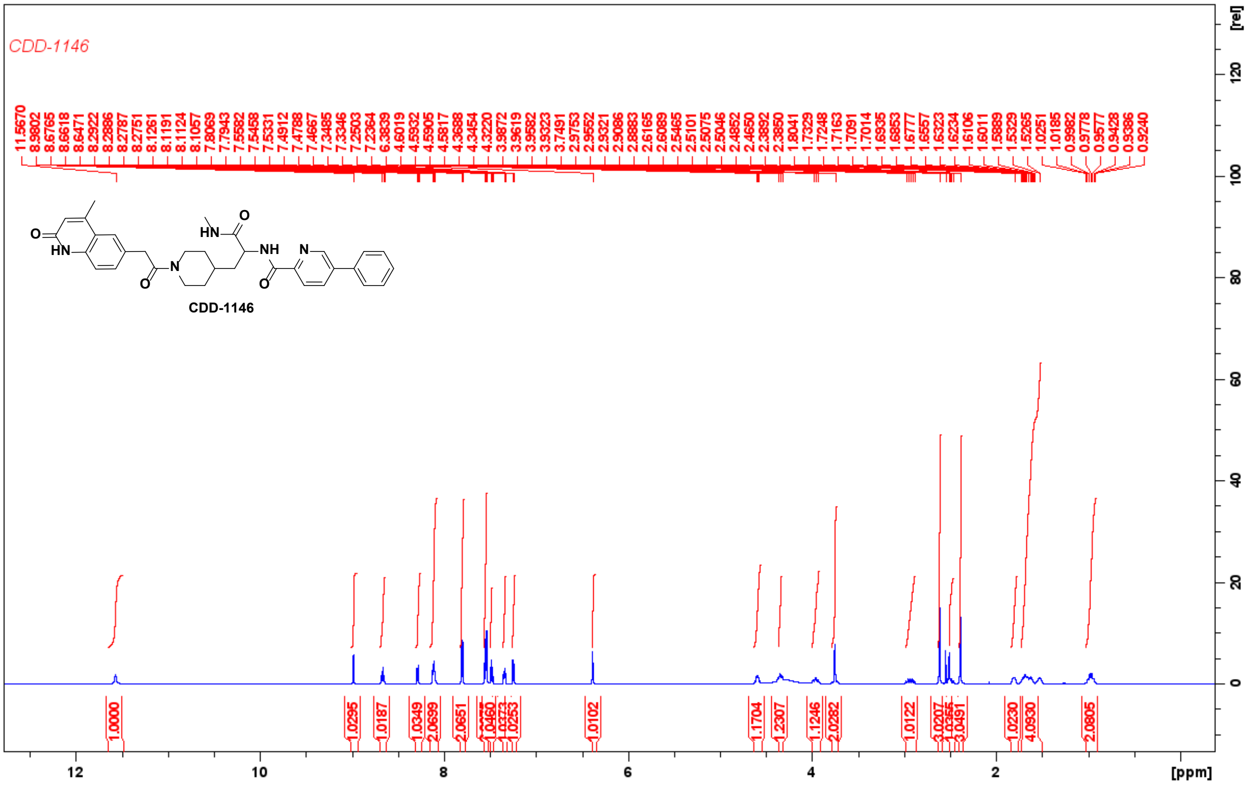

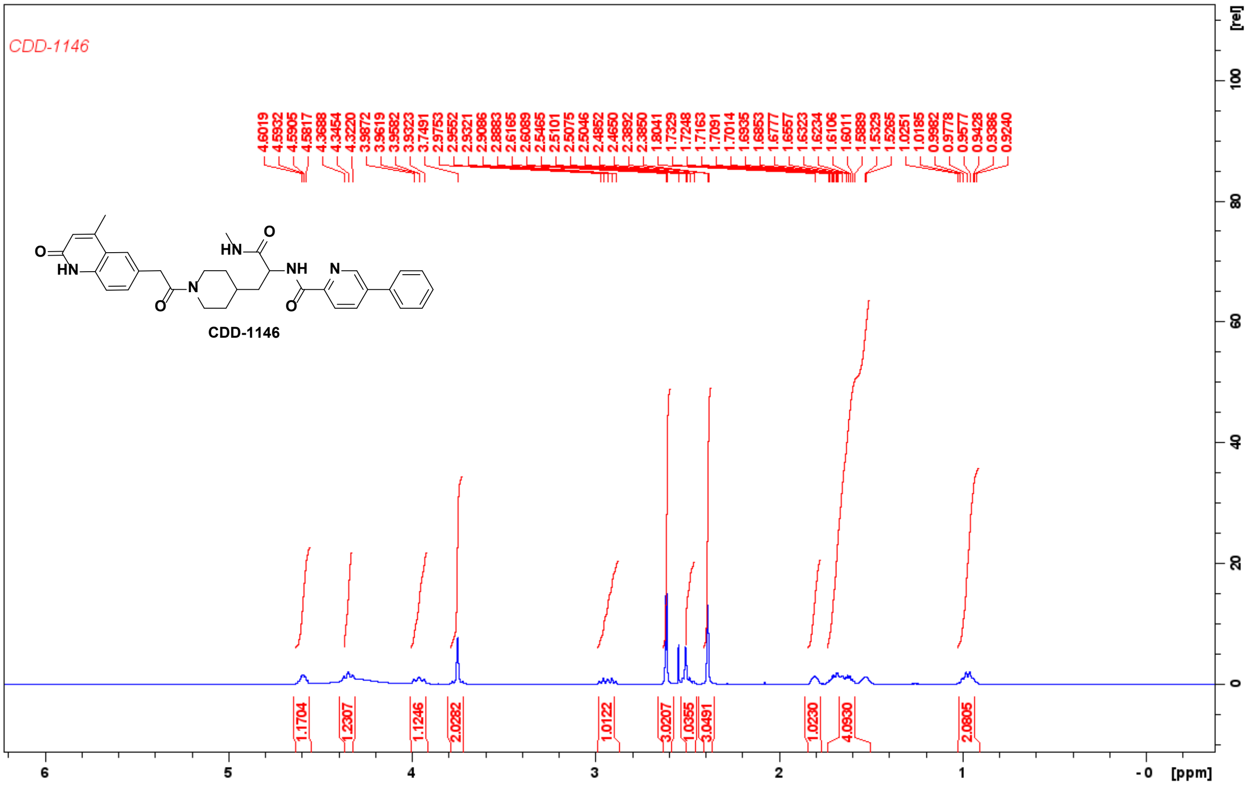

^{1}H NMR (DMSO-d$_6$, 600 MHz) and zoom-in (0–5 ppm) spectra of **6j** (**CDD-1146**)

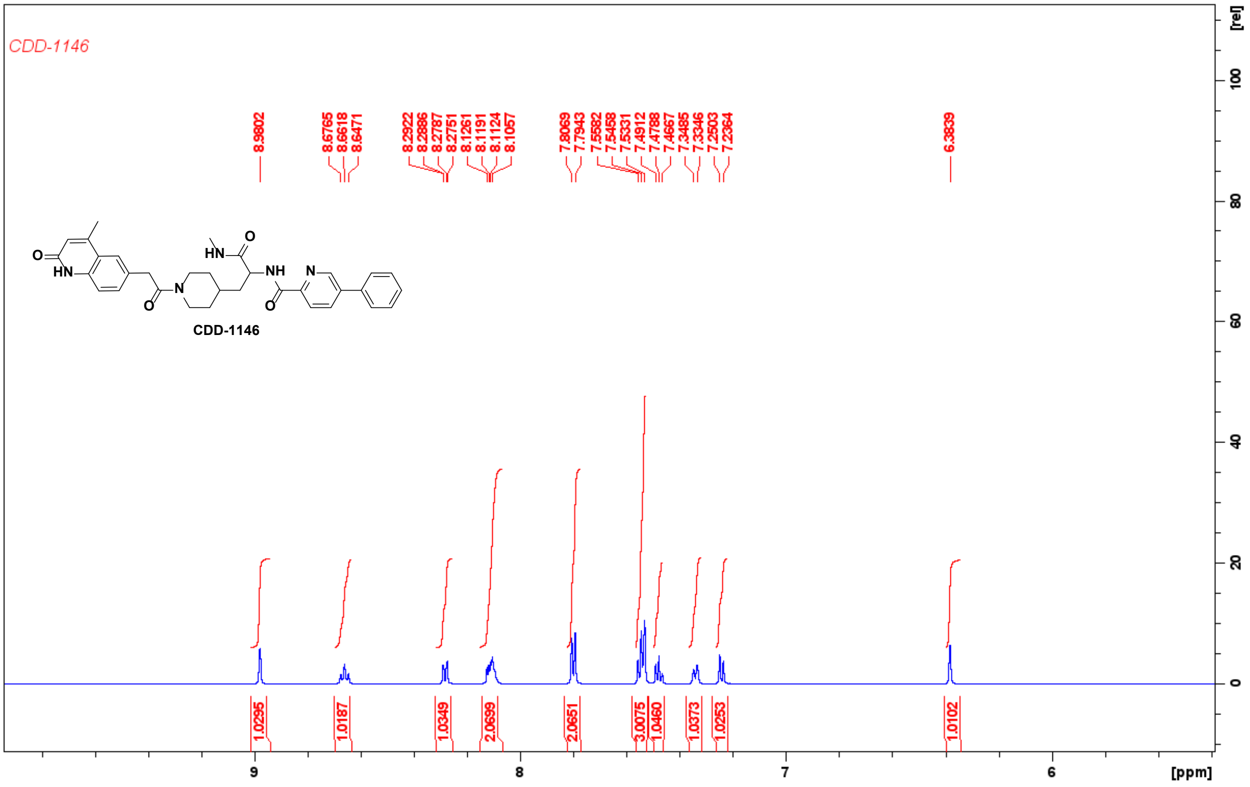

^{1}H NMR (DMSO-d_6, 600 MHz) and zoom-in (6–10 ppm) spectra of **6j** (**CDD-1146)**

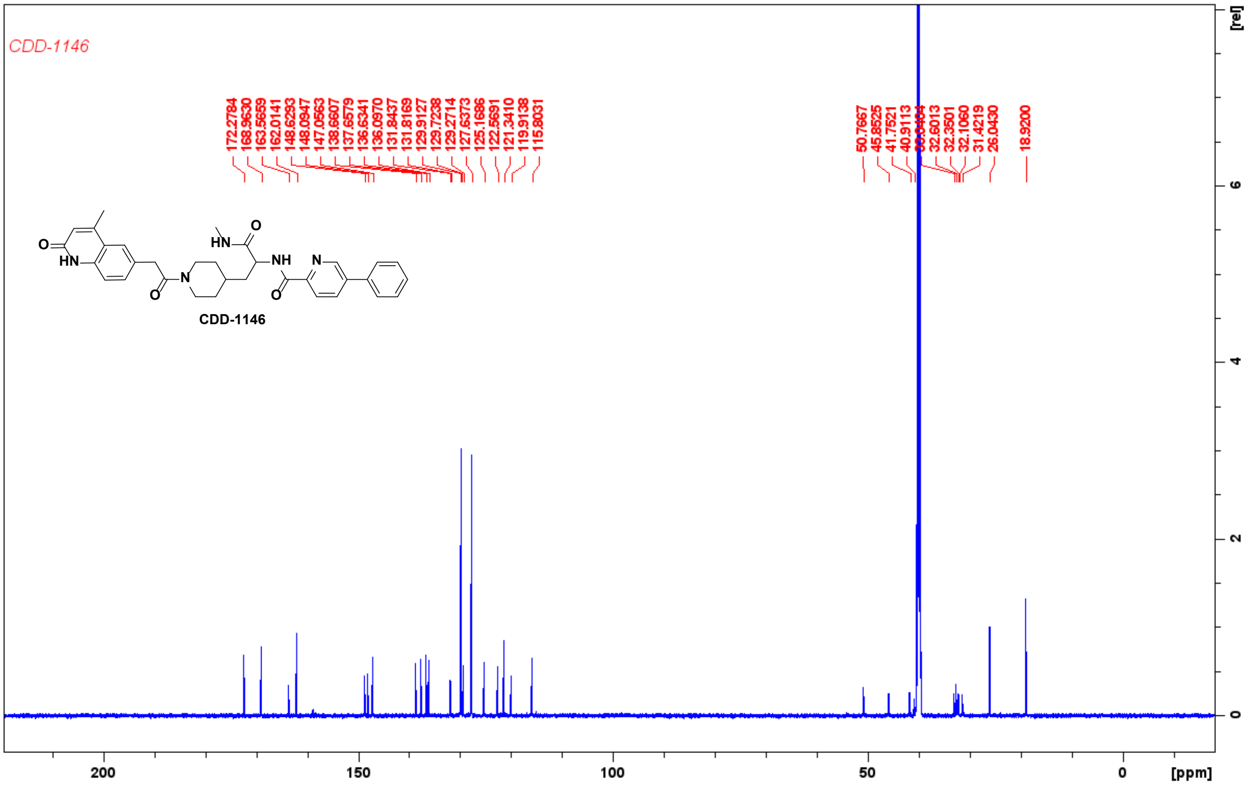

^{13}C NMR (DMSO-d_6, 150 MHz) spectrum of **6j** (**CDD-1146**)

3. Tier 2 SAR analysis

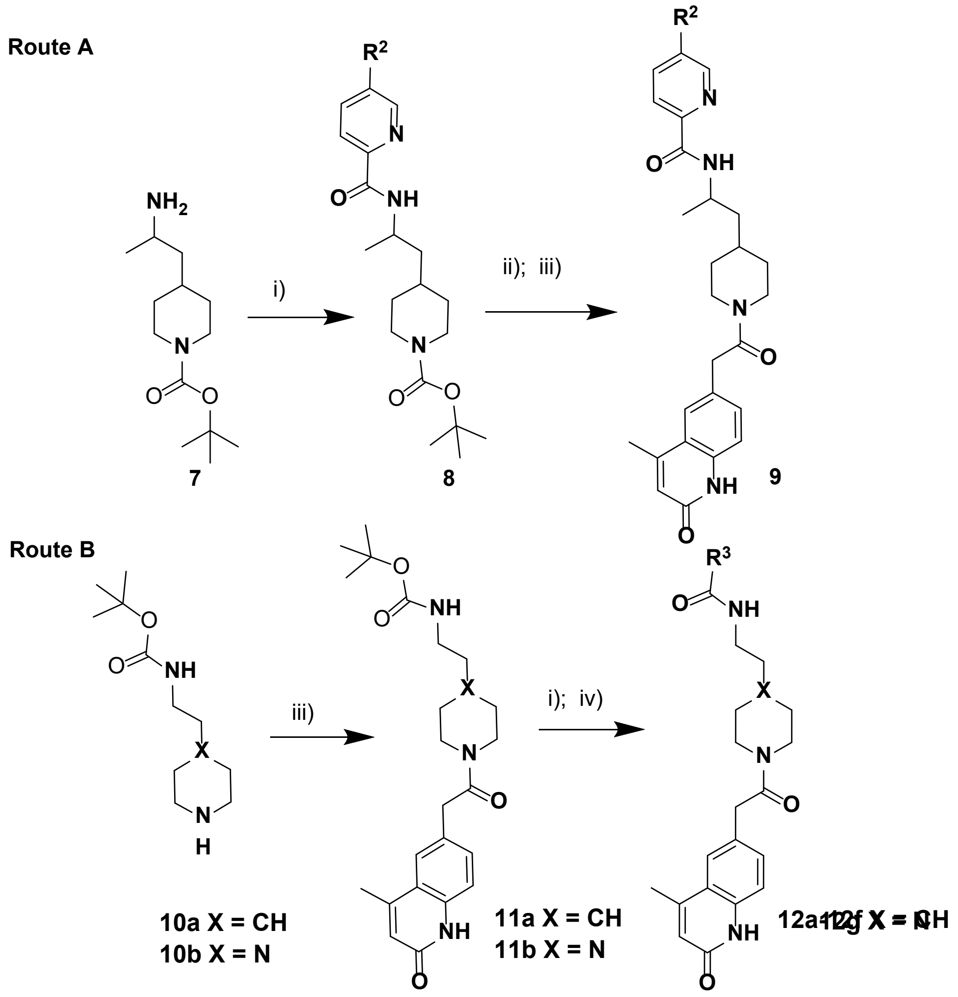

Scheme S1. Syntheses of DEC-Tec selection hits and Tier 2 SAR 9 and **12a-12g**. Reagents and conditions: i) different substituted picolinic acids, HATU, DIEA, DMF, 12h; ii) 4M HCl/dioxane, 1h; iii) 2-(4-methyl-2-oxo-1,2-dihydroquinolin-6-yl) acetic acid, HATU, DIEA, DMF, 12h; iv) different quinolines amines, HATU, DIEA, DMF, 12h.

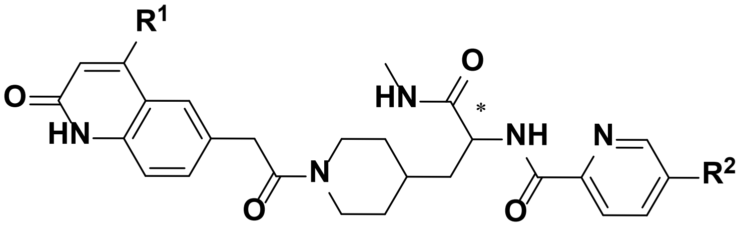

Table S1. Structures and activities of analogues 6h, 6i, and 6j. ND, Not determined.

Compound	R[1]	R[2]	*Chir-ality	BRDT-BD1 IC_{50} nM	BRDT-BD2 IC_{50} nM	BRDT-BD1 vs. BRDT-BD2 fold	BRD4-BD1 IC_{50} nM	BRD4-BD2 IC_{50} nM	BRD4-BD1 vs. BRD4-BD2 fold
**6h** (**CDD-986**)	H	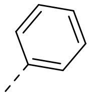	*R*	5.4	20,930	3,875	7.1	16,701	2,352
**6i** (**CDD-784**)	CH_3	H	*Rac*	20.2	19,400	960	15.7	32,640	2,078
**6j** (**CDD-1146**)	CH_3	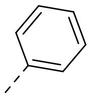	*Rac*	7.6	ND	ND	ND	ND	ND

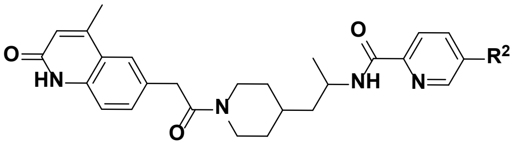

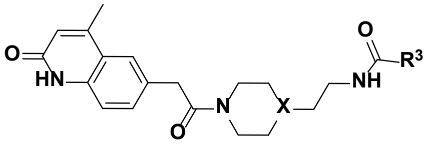

Table S2. Structures and activities of analogues 9, 12a-12g. NE, Not efficient.

Compound	R^2	R^3	X	BRDT-BD1 IC_{50} nM	BRDT-BD2 IC_{50} nM	BRDT-BD1 vs. BRDT-BD2 fold	BRD4-BD1 IC_{50} nM	BRD4-BD2 IC_{50} nM	BRD4-BD1 vs. BRD4-BD2 fold
**9 (CDD-906)**	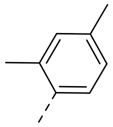			31	23,449	756	52.4	20,000	381
**12a (CDD-813)**		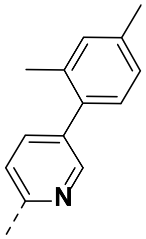	CH	33.7	67,836	2012	27.8	8,689	312
**12b (CDD-767)**		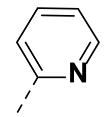	CH	35.4	6,800	192	13.8	75,200	5,449
**12c (CDD-779)**		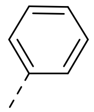	CH	148.2	10,400	70	47.9	NE	NE
**12d (CDD-855)**		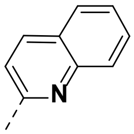	CH	81.3	NE	NE	122.4	NE	NE
**12e (CDD-854)**		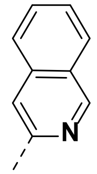	CH	115.7	NE	NE	119.4	NE	NE
**12f (CDD-853)**		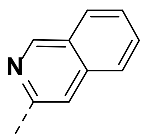	CH	638.1	NE	NE	341.4	NE	NE
**12g (CDD-778)**		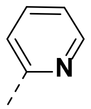	N	5200	71800	13.8	1300	NE	NE

4. Mosher Method.

We utilized the Mosher method to determine the absolute stereochemistry of α-chiral carbon of piperidine propanoic acid 3R. Two possible isomers *R* and *S* are possible at the α-carbon; to identify this, we have synthesized MTPA adducts **4 (*R*, *R*)** (R-MTPA amide) and **4 (*R*, *S*)** (*S*-MTPA amide) and carried out chiral analysis by determining Δδ*SR* (ppm). Detailed synthetic steps depicted in Scheme 3. Substituted piperidine propanoic acids (**1*R***) undergo sequential addition of O-(7-azabenzotriazol-1-yl)-N,N,N,N'-tetramethyluronium hexafluorophosphate (HATU) mediated amide coupling produce intermediate (2*R*), Subsequent deprotection of fluorenylmethyloxycarbonyl followed by amide coupling reaction furnished desired products **4 (*R*, *R*)** (R-MTPA amide) and **4 (*R*, *S*)** (*S*-MTPA amide).After making MTPA amides with **3*R***, we found that there was clear difference in the chemical shift at positions 3, 4, 5 and 9, which are affected by shielding effect of phenyl group of MTPA. Detailed chemical shifts, Δδ*SR* (ppm) and Δδ*SR* (Hz) are depicted in Table 4. Protons on position 3 and 4, adjacent to the α-carbon have shown positive values of Δδ*SR* (ppm) and Δδ*SR* (Hz), which indicates below the plane. Whereas protons on position 10 shows above the plane and shown negative values of Δδ*SR* (ppm) and Δδ*SR* (Hz). Detailed H1 NMR spectra of MTPA amides are mentioned in supporting data. From this experiment it is clear that compound 3*R* is *R* isomer and its corresponding most active compounds **CDD-787** and **CDD-956** were also *R* isomers, which are also in agreement with our positive optical rotation.

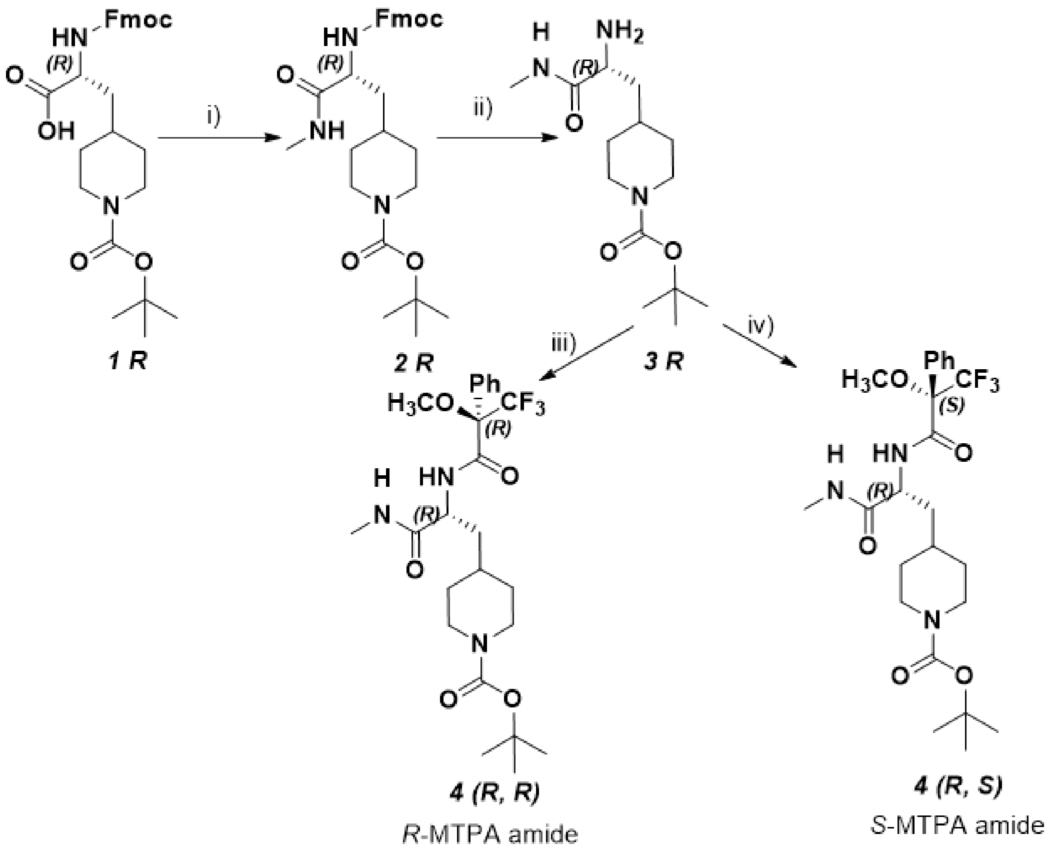

Scheme S2. Scheme for the synthesis of **MTPA** amide from ***1R***. Reagents and conditions: i) Methylamine HCl, *O*-(7-azabenzotriazol-1-yl)-*N*,*N*,*N'*,*N'*-tetramethyluronium hexafluorophosphate (HATU), *N*,*N* diisopropylethylamine (DIEA), *N*,*N*-dimethylformamide (DMF), room temperature (r.t), 1 h; ii) 10% Piperidine, DMF, 1h; iii) (*R*)-(+)-MTPA, 1,3-dicyclohexylcarbodiimide (DCC), anhydrous CH_2Cl_2 (DCM), 4-*N*,*N* (dimethylamino)pyridine (DMPA), r.t, 1 h; iv) (*S*)-(+)-MTPA, DCC, DCM, DMPA, r.t, 1 h.

Table S3. Δ δ^{SR} (=δ_S- δ_R) data for the 4 (*R*, *R*) (*R*-MTPA amide) and 4 (*R*, *S*) (*S*-MTPA amide).

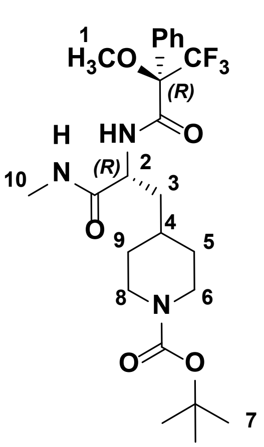

4 (R, R)

***R*-MTPA amide**

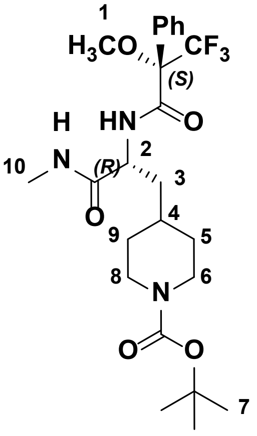

4 (R, S)

***S*-MTPA amide**

Position	δ *R*-amide 4 (*R*, *R*)	δ *S*-amide 4 (*R*, *S*)	Δδ^{SR} (=δ_S- δ_R) Δδ^{SR} (ppm)	Δδ^{SR} (Hz) Hz (600 MHz)
1	3.57, s	3.39, s	-0.18	-108
2	4.53, m	4.53, m		
3	1.65, m	1.75, m	+0.1	+60
4	1.50, m	1.69, m	+0.19	+114
5,9a	1.31, m	1.63	+0.32	+192
5,9b	1.17, m	1.52	+0.35	+210
5,9c	0.99, m	1.10, m	+0.11	+66
6,8a	3.97, m	4.05, d (13.4)	+0.08	+48
6,8b	2.50, brs	2.68, brs	+0.18	+108
7	1.44	1.44		
10	2.74, s	2.72, s	-0.02	-12

Mosher Method Spectra of 4 (*R*, *R*) (*R*-MTPA amide) and 4 (*R*, *S*) (*S*-MTPA amide)

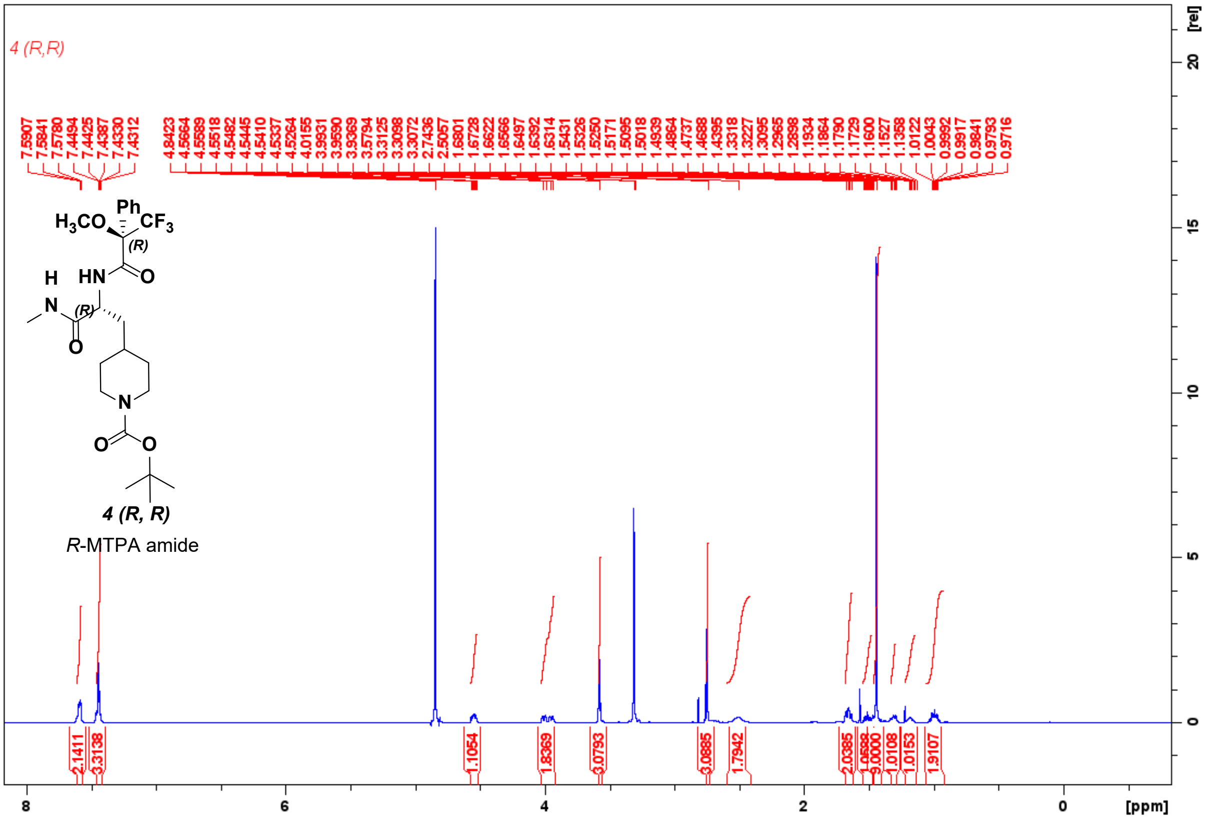

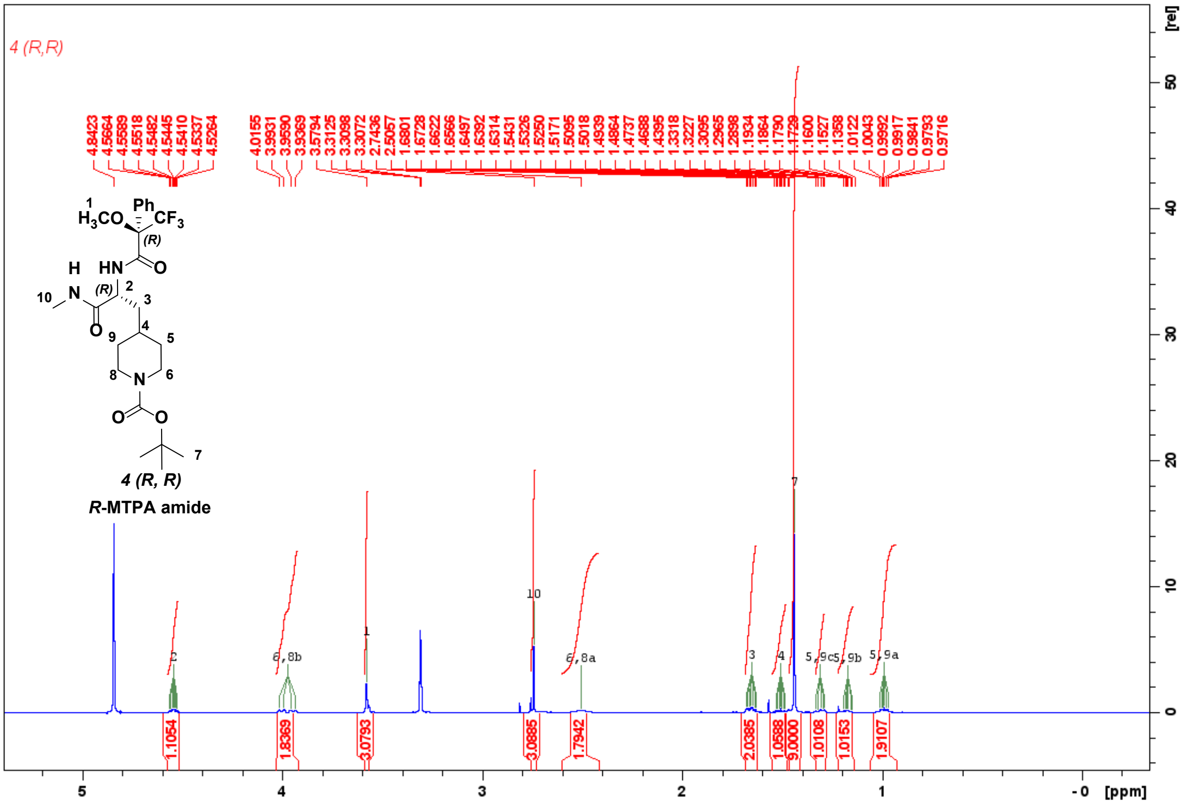

^{1}H NMR (CD_3OD, 600 MHz) and zoom-in (0–6 ppm) spectra of **4 (*R*, *R*)**

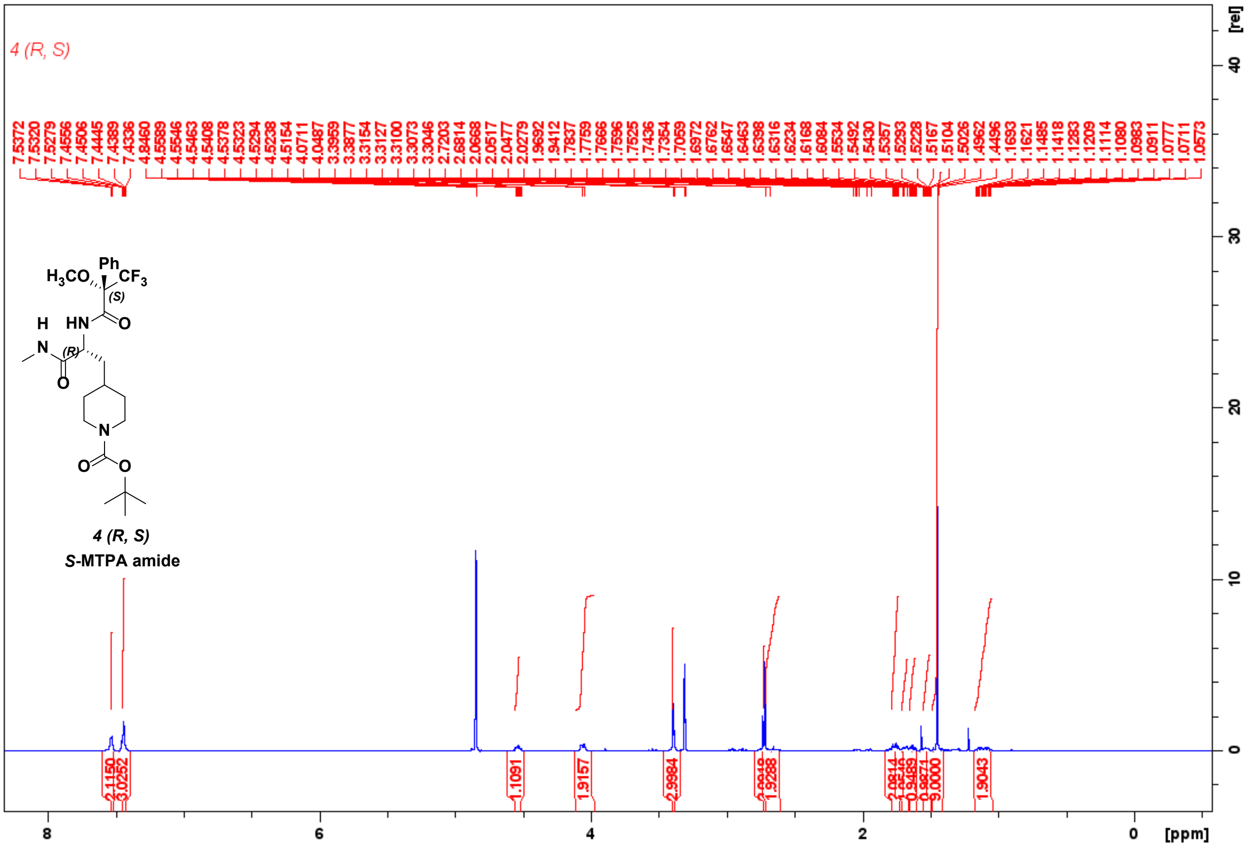

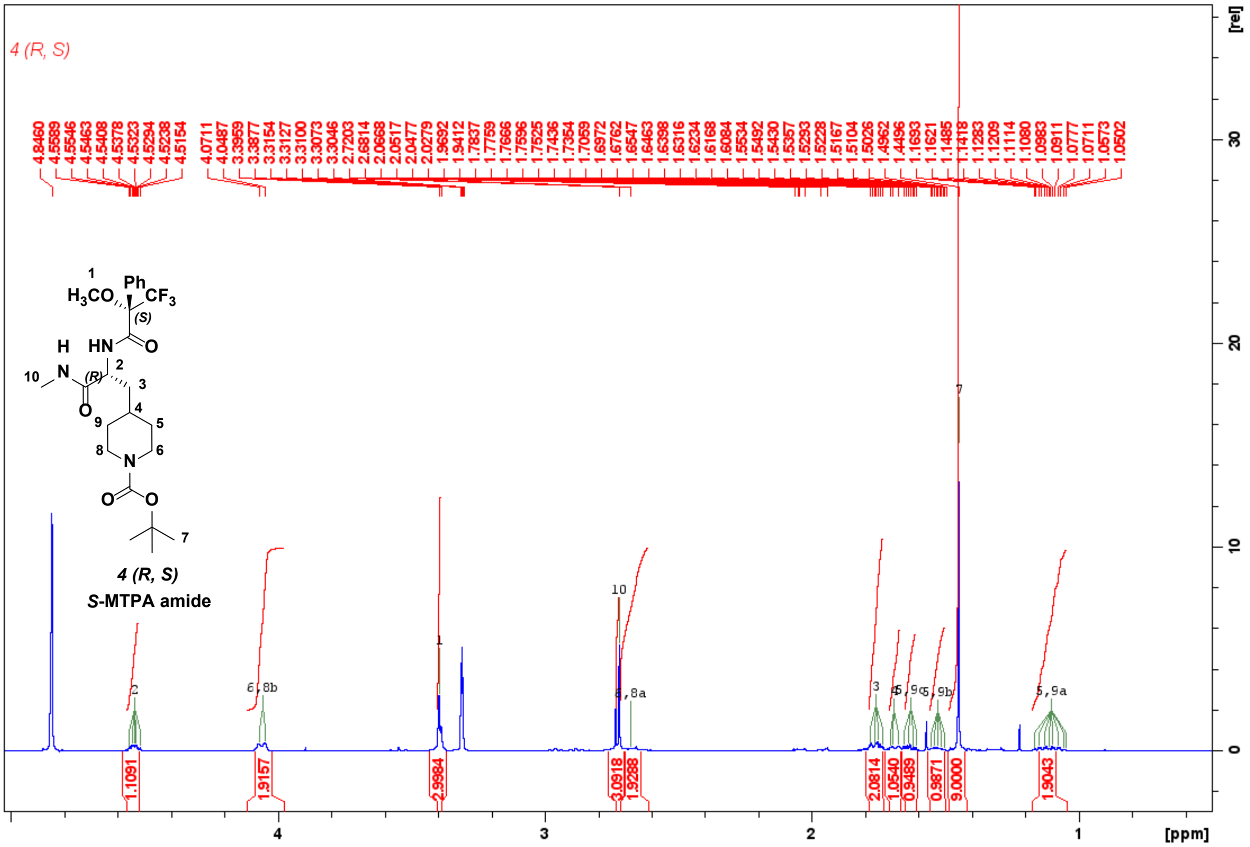

^{1}H NMR (CD_3OD, 600 MHz) and zoom-in (1–6 ppm) spectra of **4 (*R*, *S*)**

5. Chiral analysis of compounds CDD-724, CDD-786, CDD-787, CDD-1146, CDD-2107, and CDD-956.

**Column**	Chiralpak_IH_3
**Mobile phase**	Solvent A1: Heptane Solvent B2: Ethanol
**Gradient**	Isocratic: Heptane/Ethanol(40/60) for 15 minutes
**Flow rate**	0.75ml/min
**Detection**	UV 254nm
**Temperature**	ambient
**Concentration**	1mM (injection volume 5µl)
**Instrument**	Analytical HPLC

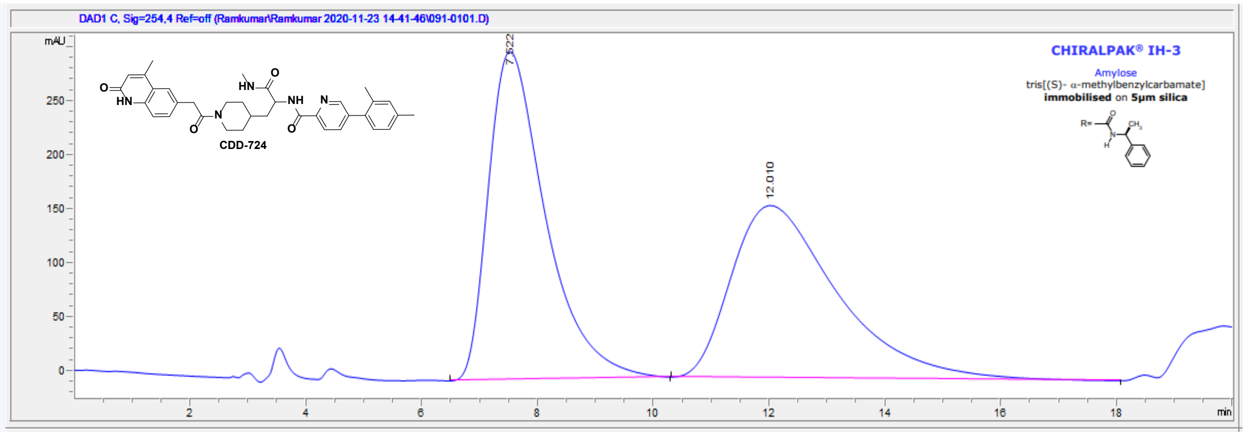

#	Time	Area	Height	Width	Area%	Symmetry
1	7.522	20928.2	304	0.9731	50.651	0.557
2	12.01	20390	159.6	1.5068	49.349	0.54

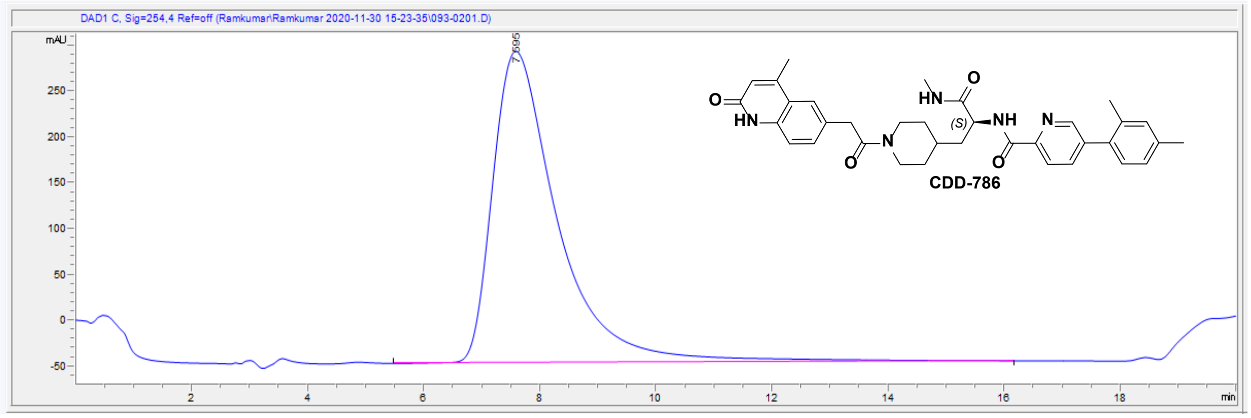

#	Time	Area	Height	Width	Area%	Symmetry
1	7.595	26745.6	341.3	1.1082	100.000	0.509

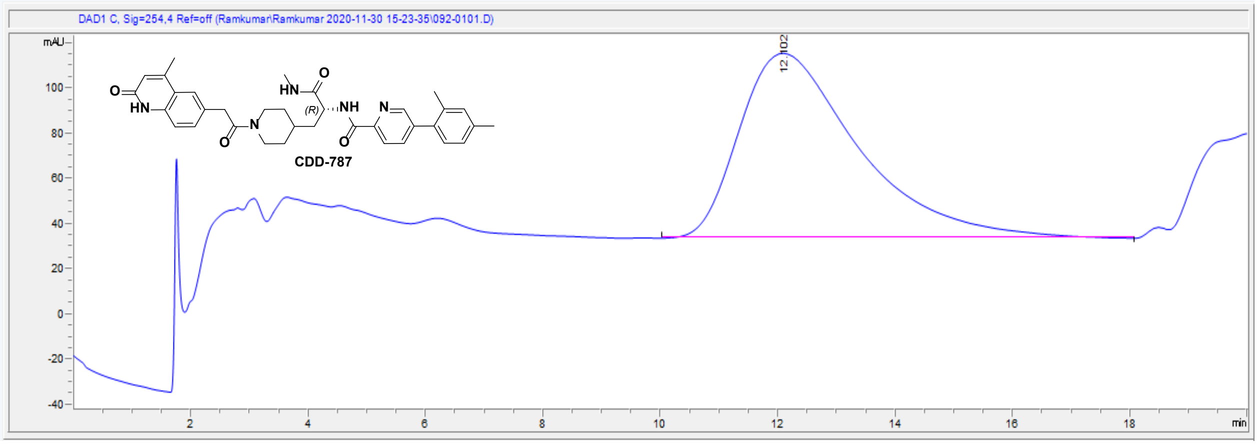

#	Time	Area	Height	Width	Area%	Symmetry
1	12.102	11636.7	82	1.6722	100.000	0.564

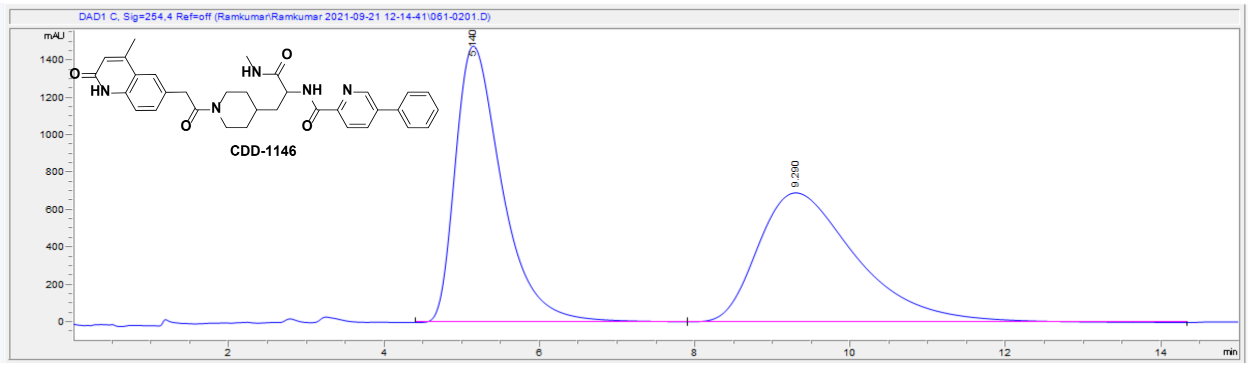

#	Time	Area	Height	Width	Area%	Symmetry
1	5.14	61929.5	1485	0.6345	50.285	0.575
2	9.29	61227.1	694.3	1.3288	49.715	0.58

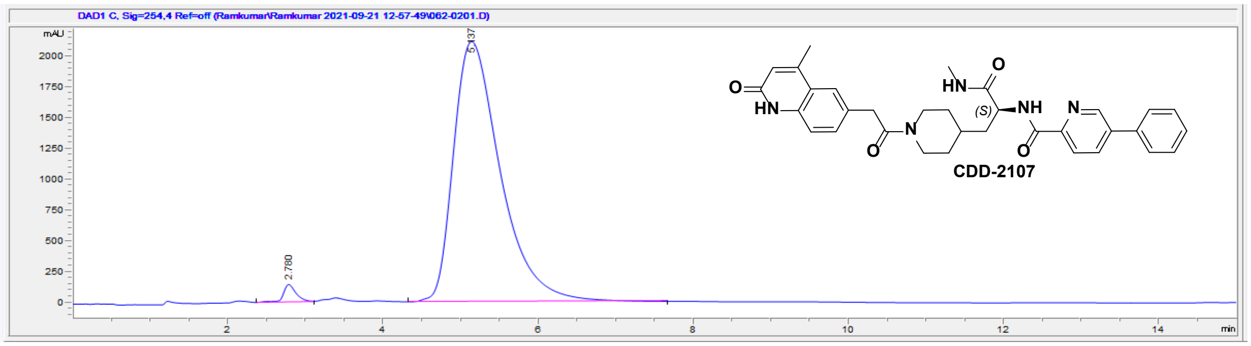

#	Time	Area	Height	Width	Area%	Symmetry
1	2.78	1732.5	144	0.1775	1.919	0.701
2	5.137	88562.4	2110.2	0.6416	98.081	0.588

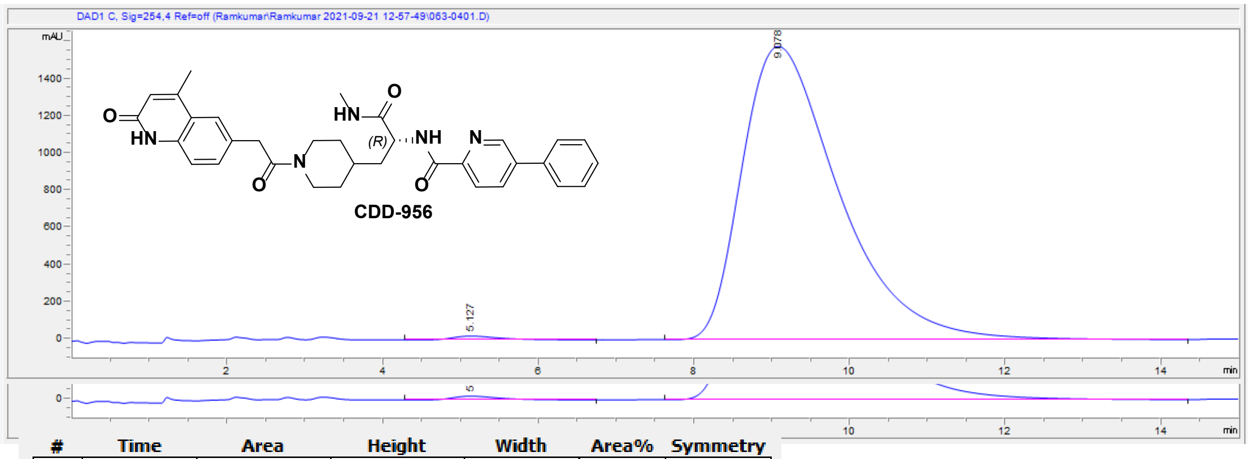

#	Time	Area	Height	Width	Area%	Symmetry
1	2.78	1732.5	144	0.1775	1.919	0.701
2	5.137	88562.4	2110.2	0.6416	98.081	0.588
1	2.78	1732.5	144	0.1775	1.919	0.701
2	5.137	88562.4	2110.2	0.6416	98.081	0.588

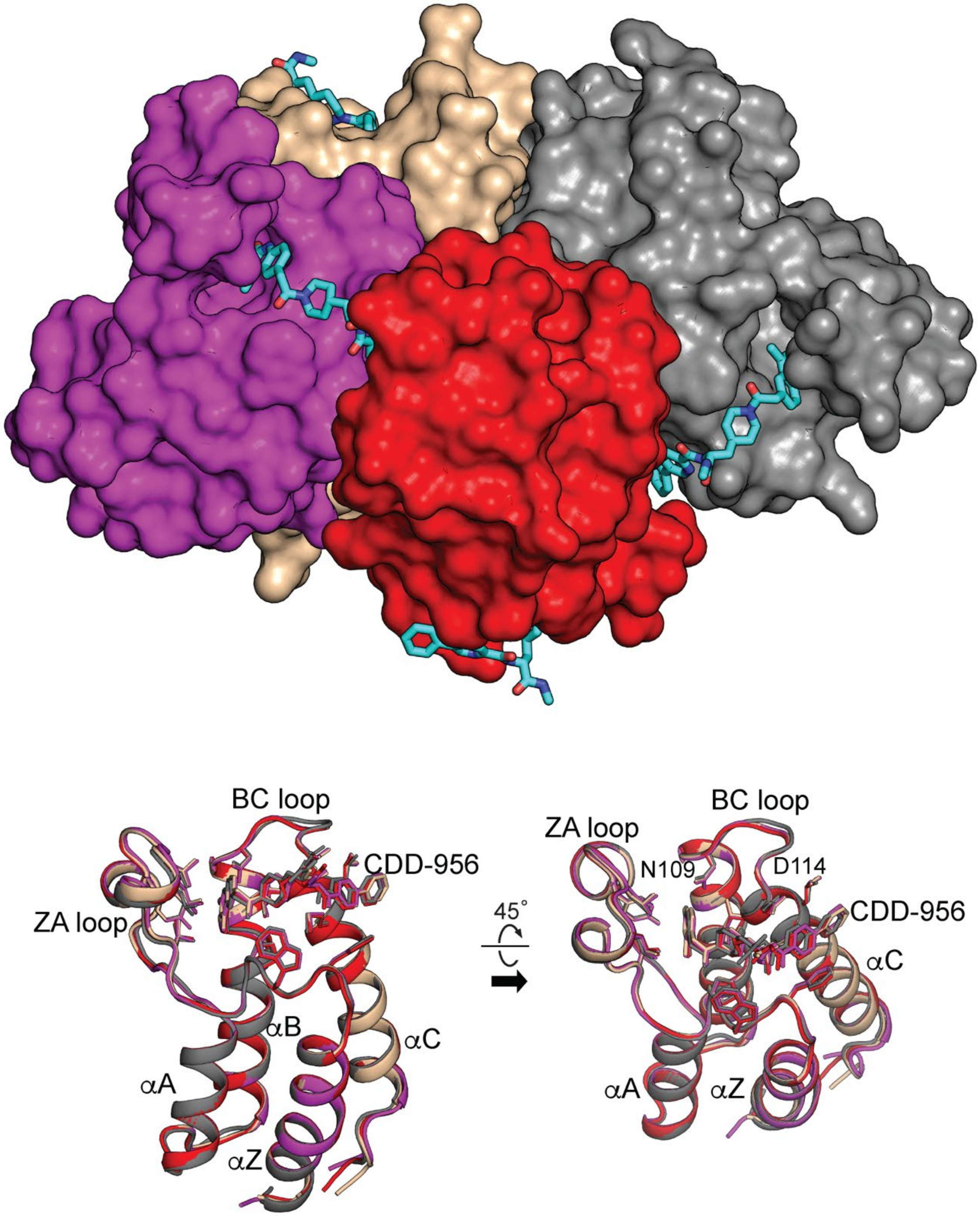

Figure S1. Four molecules captured in the BRDT-BD1/CDD-956 complex crystal.
A. Four BRDT-BD1 protein molecules are captured in the asymmetric unit with one **CDD-956** bound to each protein molecule. They are colored in tan, magenta, gray, and red. Bound **CDD-956** are shown in sticks. B. Superimposition of four BRDT-BD1/**CDD-956** complexes in the asymmetric unit. Each of the four molecular units is nearly identical (showing RMSD values less than 0.5 Å between shared ~100 CA atoms). Each molecule is shown in cartoon with side chains of key **CDD-956** interacting residues shown in sticks.

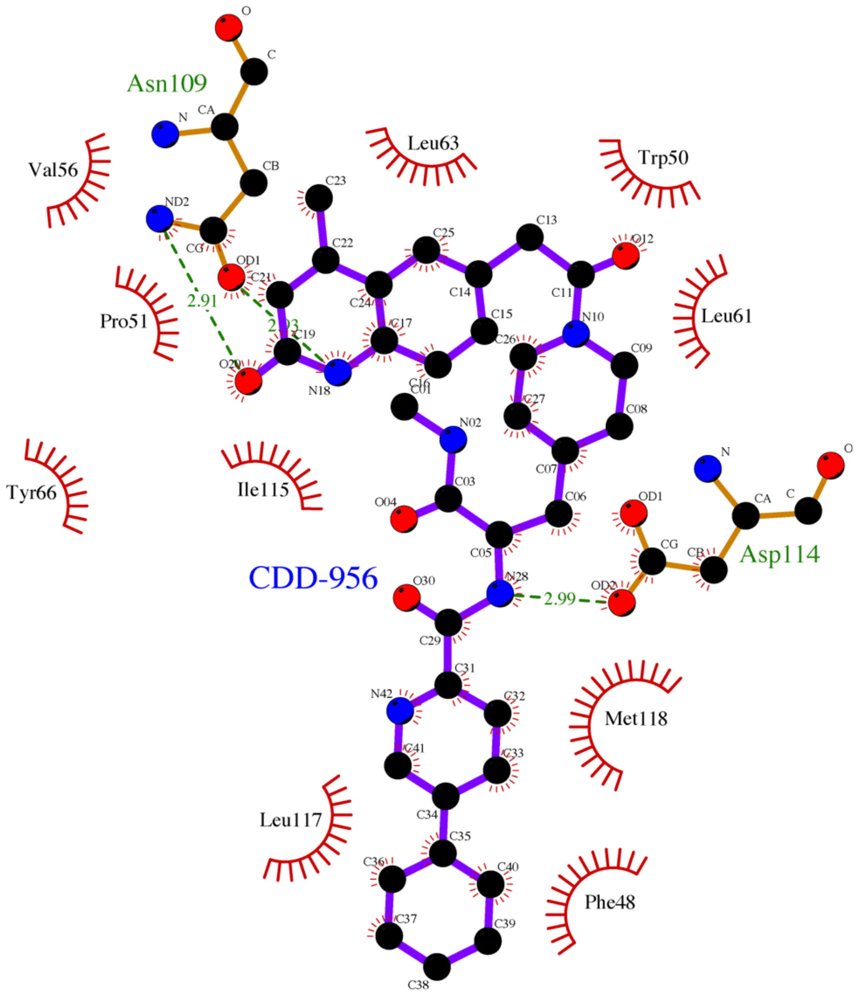

Figure S2. LIGPLOT diagram showing interactions between BRDT-BD1 and CDD-956.

6. Table S4. Data collection and refinement statistics. *Statistics for the highest-resolution shell are shown in parentheses.

Protein:ligand complex	BRDT-BD1:**CDD-956***
Wavelength	1.000
Resolution range	48.37 - 1.82 (1.885 - 1.82)
Space group	$P\ 2_1\ 2_1\ 2_1$
Unit cell	61.69, 94.38, 112.7 90 90 90
Total reflections	759141 (35916)
Unique reflections	59693 (5882)
Multiplicity	12.7 (6.1)
Completeness (%)	99.98 (100.00)
Mean I/sigma(I)	8.30 (0.72)
Wilson B-factor	25.72
R-merge	0.2041 (2.094)
R-meas	0.2122 (2.288)
R-pim	0.05729 (0.9129)
CC1/2	0.991 (0.306)
CC*	0.998 (0.684)
Reflections used in refinement	59690 (5882)
Reflections used for R-free	1909 (189)
R-work	0.1777 (0.3321)
R-free	0.1834 (0.3363)
CC(work)	0.967 (0.641)

CC(free)	0.971 (0.548)
Number of non-hydrogen atoms	4230
macromolecules	3581
ligands	178
solvent	477
Protein residues	434
RMS(bonds)	0.005
RMS(angles)	0.84
Ramachandran favored (%)	98.83
Ramachandran allowed (%)	0.94
Ramachandran outliers (%)	0.23
Rotamer outliers (%)	0.51
Clashscore	6.70
Average B-factor	33.91
macromolecules	33.15
ligands	29.71
solvent	41.15
Number of TLS groups	26

7. Table S5. Matrix of CDD-956 screen in BROMO*scan* assays. Percent Control (%Ctrl): CDD-956 was screened at 1000 nM, and results for binding interactions are reported as '% Ctrl', where lower numbers indicate stronger hits in the matrix. %Ctrl Calculation: (CDD-956 signal-positive control signal)/(negative control signal-positive control signal)*100 negative control = DMSO(100%Ctrl), positive control = control compound (0%Ctrl).

Compound Name	DiscoveRx Gene Symbol	Entrez Gene Symbol	Percent Control	Compound Concentration (nM)
CDD-956	ATAD2A	ATAD2	100	1000
CDD-956	ATAD2B	ATAD2B	97	1000
CDD-956	BAZ2A	BAZ2A	100	1000
CDD-956	BAZ2B	BAZ2B	100	1000
CDD-956	BRD1	BRD1	96	1000
**CDD-956**	**BRD2(1)**	**BRD2**	**0**	**1000**
**CDD-956**	**BRD2(2)**	**BRD2**	**2**	**1000**
**CDD-956**	**BRD3(1)**	**BRD3**	**0**	**1000**
**CDD-956**	**BRD3(2)**	**BRD3**	**26**	**1000**
**CDD-956**	**BRD4(1)**	**BRD4**	**0**	**1000**
**CDD-956**	**BRD4(2)**	**BRD4**	**78**	**1000**
CDD-956	BRD7	BRD7	95	1000
CDD-956	BRD9	BRD9	90	1000
**CDD-956**	**BRDT(1)**	**BRDT**	**0**	**1000**
**CDD-956**	**BRDT(2)**	**BRDT**	**68**	**1000**
CDD-956	BRPF1	BRPF1	66	1000
CDD-956	BRPF3	BRPF3	100	1000
CDD-956	CECR2	CECR2	100	1000
CDD-956	CREBBP	CREBBP	100	1000
CDD-956	EP300	EP300	100	1000
CDD-956	FALZ	BPTF	100	1000
CDD-956	GCN5L2	KAT2A	99	1000
CDD-956	PBRM1(2)	PBRM1	100	1000
CDD-956	PBRM1(5)	PBRM1	100	1000
CDD-956	PCAF	KAT2B	100	1000
CDD-956	SMARCA2	SMARCA2	100	1000
CDD-956	SMARCA4	SMARCA4	100	1000
CDD-956	TAF1(2)	TAF1	88	1000
CDD-956	TAF1L(2)	TAF1L	100	1000
CDD-956	TRIM24(PHD,Bromo.)	TRIM24	100	1000
CDD-956	TRIM33(PHD,Bromo.)	TRIM33	84	1000
CDD-956	WDR9(2)	BRWD1	100	1000

8. Table S6. Viability EC_{50} of selected compounds against AML cell line MV4;11. NE, Not efficient.

Compound	MV4-11 EC_{50} nM
**CDD-724**	NE
**CDD-787**	8
**CDD-956**	21
**CDD-981**	58
**CDD-982**	100
**CDD-986**	143
**CDD-1146**	NE

9. Materials and Methods.

Production of recombinant bromodomain proteins: Recombinant His6-tagged BRDT-BD1 and BD2 were produced as previously described (1)(1)(1)(1)(1)(1). In brief, cDNA encoding human BRDT and BRD4 BD1 and BD2 bromodomains were subcloned into pET15b or pET28b bacterial vectors (Addgene, USA) with an N-terminal polyhistidine tag (6-His; linear) for expression and purification. His-tagged recombinant protein was isolated and eluted by immobilized metal affinity chromatograph followed by size exclusion gel filtration chromatography. Each BD was confirmed to be properly folded and active by a fluorescence thermal shift stability assay and AlphaScreen with biotinylated JQ1 as ligand, respectively.

DEC-Tec affinity selection with bromodomain proteins. To identify BRDT-BD1 selective compounds, we screened our DEC-Tec library pool. We had five screening conditions: 1) absence of bromodomain proteins (bead binding non-target control, NTC); 2) presence of His-BRDT-BD1 at 0.3 μM; 3) presence of His-BRDT-BD2 at 0.3 μM (a counter-screen for bromodomain selective compounds); 4) presence of His-BRDT-BD1 plus JQ1 at 100 μM; and 5) presence of His-BRDT-BD2 plus JQ1 at 100 μM. After three rounds of DEC-Tec selection, the DNA barcode from the last round of selection was PCR amplified. Following cleanup by Agencourt AMPure XP beads and quantitation with Agilent high sensitivity DNA kit using a Bioanalyzer, the DNA was sequenced in a single-read 105-cycle sequencing on an Illumina NextSeq 500 instrument.

Bromodomain proximity assay. The AlphaScreenTM assay was performed following previous publication with minor modifications from the manufacturer's protocol (PerkinElmer, USA). A 20-μL reaction was set up in a PerkinElmer 384-well AlphaPlate where His-bromodomain at 10 nM was

incubated with biotinylated JQ1 at 10 nM, nickel chelate acceptor beads at 12.5 μg/mL, and tested compound at various concentrations for 15 min at room temperature, followed by the addition of streptavidin donor beads at 12.5 μg/mL and another 60-min incubation at room temperature. The plate was read on a Tecan Infinite M1000 Pro plate reader.

Thermal shift assay. The dye SYPRO Orange (ThermoFisher Scientific, USA) was used to perform the protein thermal shift assay. The assay was set up on a 384-well Roche plate where His-bromodomain at a concentration of 2 μM was incubated with the test compound at various concentrations, and SYPRO Orange dye at 5 × in a 10-μL reaction. The melting curve experiment and data analysis was run on a Roche Lightcycler 480 real-time PCR instrument.

Metabolic stability assay in liver microsomes. CDD-787 and **CDD-956** (2.0 μM) were incubated in the mouse or human liver microsomes (0.5 mg protein/mL) at 37 °C in the presence of NADPH (1.0 mM). The samples are collected at specific time-points 0, 5, 10, 20, 40 and 60 min in duplicate. The reactions are terminated by adding equivalent volume of ice-cold CH_3OH and vortexed. The reaction mixtures are centrifuged at 15,000 g for 15 min. Five μL of the supernatant was analyzed by ultra-high performance liquid chromatography (UHPLC)-MS/MS analysis (QQQ, Agilent 6490) equipped with 50 mm × 4.6 mm column (XDB C-18, Agilent Technologies, USA). LC-MS/MS (Agilent Technologies, QQQ, Santa Clara, CA) was operated in positive mode with electrospray ionization. Ultrapure nitrogen was applied as the sheath gas and the collision gas. The capillary gas temperature was set at 280 °C and the capillary voltage was set at 3.6 kV. The column temperature was maintained at 40 °C. The flow rate was at 0.3 mL/min with a 50% mobile phase (acetonitrile containing 0.1% formic acid) in a 6-min run. **CDD-787** and **CDD-956** were measured using multiple-reaction monitoring method with the mass transition *m/z* 594.3>318.1 for **CDD-787**; *m/z* 566.3>290.1 for **CDD-956**. JQ1 (mass transition:*m/z* 457.4>341.3) and alprazolam (*m/z* 309.2>281.3) were used as the short and long half-life control, respectively.

***BromoKd*ELECT assays.** Assays were performed at Eurofins DiscoverX (Fremont, CA) using the following procedures. T7 phage strains displaying bromodomains were grown in parallel in 24-well blocks in an E. coli host derived from the BL21 strain. E. coli were grown to log-phase and infected with T7 phage from a frozen stock (multiplicity of infection = 0.4) and incubated with shaking at 32°C until lysis (90-150 minutes). The lysates were centrifuged (5,000 x g) and filtered (0.2μm) to remove cell debris. Streptavidin coated magnetic beads were treated with biotinylated small molecule or acetylated peptide

ligands for 30 minutes at room temperature to generate affinity resins for bromodomain assays. The liganded beads were blocked with excess biotin and washed with blocking buffer (SeaBlock (Pierce), 1 % BSA, 0.05 % Tween 20, and 1 mM DTT) to remove unbound ligand and to reduce non specific phage binding. Binding reactions were assembled by combining bromodomains, liganded affinity beads, and test compounds in 1x binding buffer (16 % SeaBlock, 0.32x PBS, 0.02%BSA, 0.04 % Tween 20, 0.004% Sodium azide, and 7.9 mM DTT). Test compounds were prepared as 1000X stocks in 100% DMSO and subsequently diluted 1:25 in monoethylene glycol (MEG). The compounds were then diluted directly into the assays such that the final concentrations of DMSO and MEG were 0.1% and 2.4%, respectively. All reactions were performed in polypropylene 384 well plates in a final volume of 0.02 ml. The assay plates were incubated at room temperature with shaking for 1 hour and the affinity beads were washed with wash buffer (1x PBS with 0.05% Tween 20). The beads were then re-suspended in elution buffer (1x PBS with 0.05% Tween 20 and 2 μM of non-biotinylated affinity ligand) and incubated at room temperature with shaking for 30 minutes. The bromodomain concentration in the eluates was measured by qPCR.

Crystallization, data collection, and structure solution. BRDT-BD1 was co-crystallized with **CDD-956** by the hanging drop vapor diffusion method. For crystallization, purified BRDT-BD1 at 3.5 mg/ml was mixed with a 5 molar excess of **CDD-956**. The protein-inhibitor mixture was concentrated using Amicon Ultra-15 centrifugal filters (Millipore Sigma) to 24 mg/ml. Mosquito (TTP labtech) was used to dispense equal volumes of protein and reservoir (250 nl each) against 70 μl reservoir buffer in 96 wells crystallization tray (96-Well clear polystyrene microplate from SPT Labtech). Crystals of the BRDT-BD1/**CDD-956** complex were observed after 2 days in drop containing 1.26 M Ammonium Sulfate, 0.1 M Tris (pH 8.5), and 0.2 M Lithium sulfate. The crystals were cryo-cooled in the same solution with 20 % glycerol. The diffraction data were collected at Advanced Photo Source (APS) (Lemont, Illinois, USA). The data was integrated and scaled by using iMosflm and SCALA (2, 3). The crystal structure of the BRDT-BD1/**CDD-956** complex was determined by molecular replacement in PHENIX using a crystal structure of the human BRDT BD1 (PDB ID: 4FLP) as a search model (4). **CDD-956** was fitted manually into electron density by using COOT. The final models have gone through several rounds of refinement using Phenix.refine (5) followed by manual model building using COOT (6). For all structural analysis and preparation of figures, the visualization program PyMOL was used (7).

NanoBRET target engagement intracellular BET bromodomain assay. The NanoLuc-BRDT-BD1 and NanoLuc-BRDT-BD2 vectors were constructed by subcloning the same BRDT bromodomain

sequences applied for protein expression into the NanoLuc-BRD4-BD1 and NanoLuc-BRD4-BD2 vectors (Promega, USA) to replace the corresponding BRD4 bromodomain sequences. The NanoBRET tracer competition assay was performed in transiently transfected HEK293 cells expressing each NanoLuc-bromodomain vector on a 384-well plate following the manufacturer's protocol (Promega, USA). Tracer titration was performed for each NanoLuc fusion vector to determine the optimized tracer concentration. The fusion protein was allowed to express for 36 h. The cells were then preincubated with tested compounds at different concentrations for 2 h followed by 2 h of incubation with tracer. Freshly prepared NanoBRET Nano-Glo substrate plus extracellular NanoLuc inhibitor was then added to initiate the subsequent bioluminescence resonance energy transfer (BRET) measurements using a CLARIOstarPlus BMG LABTECH plate reader. Data analysis was done by measuring the ratio of acceptor emission to donor emission (BRET ratio) and normalized by subtracting no-tracer-control-background.

Cell culture. AML cell lines MV4;11, MOLM-13, THP-1, and Kasumi1 were graciously shared by Dr. Stevens at Texas Children's Hospital. Cell lines were incubated at 37°C under 5% CO_2, in RPMI-1640 plus 10% FBS and 1% Penicillin/Streptomycin. Mycoplasma testing was performed using the LookOut Mycoplasma qPCR Detection Kit (Sigma) at entry into the lab. Cell line identity was confirmed annually using STR fingerprinting at the Cytogenetics and Cell Authentication Core at MD Anderson Cancer Center. Cells used for experiments were <20 passages from thawing.

Cell viability testing. Dose-response testing of CDD hits were conducted on 40,000 cells/mL plated in 384-well plates with 4 ten-fold dilutions of tamibarotene and read out by CellTiterGlo (Promega) at 72 hours of treatment per manufacturer instructions. There were at least 3 biologic replicates for each condition, and experiments were independently repeated three times to confirm similar results. Analysis was done using GraphPad Prism normalizing to the CellTiterGlo signal of DMSO-treated samples as indicated.

Flow cytometry. Cells were harvested after 24 hours treatment for cell cycle analysis. After a PBS rinse, cells were fixed overnight in 70% methanol at -20 °C. The following day, samples were rinsed with 0.5% BSA in PBS, then incubated in 100 μg/mL RNAse/PBS at 37 °C for 15 minutes. Then cells were resuspended in 50 μg/mL PI in PBS. The samples were then analyzed on the BD canto II flow cytometer. For apoptosis detection, cells were harvested at 72 hours of treatment. Media was aspirated to leave a residual of 300-400uL. DAPI and AlexaFluor 488-conjugated Annexin V antibody (1:200 dilution,

ThermoFisher A13201) was added to the tube in equal volume, and samples were analyzed in the LSR I flow cytometer. For both experiments, three biologic replicates were averaged for each condition, and experiments were all repeated three times to confirm results. Results were analyzed using FlowJo v10 and plotted in GraphPad Prism using an unpaired t-test to compare statistical significance.

Quantitative RT-PCR analysis. Expression of MYC and GAPDH were determined using real-time quantitative reverse transcriptase-polymerase chain reaction (qPCR). Three technical replicates per sample were used. Primers for MYC, GAPDH, and housekeeping control ACTB were ordered from Sigma-Millipore. Relative expression was calculated using the comparative ΔΔCt method normalizing to ACTB and DMSO. All results were plotted in GraphPad Prism using an unpaired t-test to determine statistical significance.

Coordinates. Atomic coordinates and structure factors of the BRDT BD1/**CDD-956** complex have been deposited in the Protein Data Bank (www.pdb.org) under an identification number 7UBO.

10. Supporting References.

1. Z. Yu *et al.*, Discovery and characterization of bromodomain 2-specific inhibitors of BRDT. *Proc Natl Acad Sci U S A* **118** (2021).
2. N. Collaborative Computational Project, The CCP4 suite: programs for protein crystallography. *Acta Crystallogr D Biol Crystallogr* **50**, 760-763 (1994).
3. T. G. Battye, L. Kontogiannis, O. Johnson, H. R. Powell, A. G. Leslie, iMOSFLM: a new graphical interface for diffraction-image processing with MOSFLM. *Acta Crystallogr D Biol Crystallogr* **67**, 271-281 (2011).
4. P. D. Adams *et al.*, PHENIX: building new software for automated crystallographic structure determination. *Acta Crystallogr D Biol Crystallogr* **58**, 1948-1954 (2002).
5. P. V. Afonine *et al.*, Towards automated crystallographic structure refinement with phenix.refine. *Acta Crystallogr D Biol Crystallogr* **68**, 352-367 (2012).
6. P. Emsley, K. Cowtan, Coot: model-building tools for molecular graphics. *Acta Crystallogr D Biol Crystallogr* **60**, 2126-2132 (2004).
7. W. L. DeLano, The PyMOL Molecular Graphics System, San Carlos, CA, USA. (2002).
